# Trees, fungi and bacteria: tripartite metatranscriptomics of a root microbiome responding to soil contamination

**DOI:** 10.1186/s40168-018-0432-5

**Published:** 2018-03-21

**Authors:** E. Gonzalez, F. E. Pitre, A. P. Pagé, J. Marleau, W. Guidi Nissim, M. St-Arnaud, M. Labrecque, S. Joly, E. Yergeau, N. J. B. Brereton

**Affiliations:** 1 Canadian Center for Computational Genomics, McGill University and Genome Quebec Innovation Center, Montréal, H3A 1A4 Canada; 2 Department of Human Genetics, McGill University, Montreal, H3A 1B1 Canada; 3 Institut de recherche en biologie végétale, University of Montreal, Montreal, QC H1X 2B2 Canada; 4 Montreal Botanical Garden, Montreal, QC H1X 2B2 Canada; 5 Aquatic and Crop Resource Development (ACRD), National Research Council Canada, Montréal, QC H4P 2R2 Canada; 6 Department of Agri-food and Environmental Science, University of Florence, Viale delle Idee, Sesto Fiorentino, FI Italy; 7 Institut National de la Recherche Scientifique, Centre INRS–Institut Armand-Frappier, Laval, QC Canada

**Keywords:** Metatranscriptomics, Microbiome, Salix, Rhizosphere, Phytoremediation

## Abstract

**Background:**

One method for rejuvenating land polluted with anthropogenic contaminants is through phytoremediation, the reclamation of land through the cultivation of specific crops. The capacity for phytoremediation crops, such as *Salix* spp., to tolerate and even flourish in contaminated soils relies on a highly complex and predominantly cryptic interacting community of microbial life.

**Methods:**

Here, Illumina HiSeq 2500 sequencing and de novo transcriptome assembly were used to observe gene expression in washed *Salix purpurea* cv. ‘Fish Creek’ roots from trees pot grown in petroleum hydrocarbon-contaminated or non-contaminated soil. All 189,849 assembled contigs were annotated without a priori assumption as to sequence origin and differential expression was assessed.

**Results:**

The 839 contigs differentially expressed (DE) and annotated from *S*. *purpurea* revealed substantial increases in transcripts encoding abiotic stress response equipment, such as glutathione S-transferases, in roots of contaminated trees as well as the hallmarks of fungal interaction, such as SWEET2 (Sugars Will Eventually Be Exported Transporter). A total of 8252 DE transcripts were fungal in origin, with contamination conditions resulting in a community shift from *Ascomycota* to *Basidiomycota* genera. In response to contamination, 1745 *Basidiomycota* transcripts increased in abundance (the majority uniquely expressed in contaminated soil) including major monosaccharide transporter MST1, primary cell wall and lamella CAZy enzymes, and an ectomycorrhiza-upregulated exo-β-1,3-glucanase (GH5). Additionally, 639 DE polycistronic transcripts from an uncharacterised *Enterobacteriaceae* species were uniformly in higher abundance in contamination conditions and comprised a wide spectrum of genes cryptic under laboratory conditions but considered putatively involved in eukaryotic interaction, biofilm formation and dioxygenase hydrocarbon degradation.

**Conclusions:**

Fungal gene expression, representing the majority of contigs assembled, suggests out-competition of white rot *Ascomycota* genera (dominated by *Pyronema*), a sometimes ectomycorrhizal (ECM) *Ascomycota* (*Tuber*) and ECM *Basidiomycota* (*Hebeloma*) by a poorly characterised putative ECM *Basidiomycota* due to contamination. Root and fungal expression involved transcripts encoding carbohydrate/amino acid (C/N) dialogue whereas bacterial gene expression included the apparatus necessary for biofilm interaction and direct reduction of contamination stress, a potential bacterial currency for a role in tripartite mutualism. Unmistakable within the metatranscriptome is the degree to which the landscape of rhizospheric biology, particularly the important but predominantly uncharacterised fungal genetics, is yet to be discovered.

**Electronic supplementary material:**

The online version of this article (10.1186/s40168-018-0432-5) contains supplementary material, which is available to authorized users.

## Background

The observation of gene expression across multiple interacting organisms has the potential to better reflect the complex reality of biology than the observation of organisms in isolation [1]. By separating the assembly of RNA sequence data from annotation (identification) of assembled contigs, de novo metatranscriptome assembly allows for such observation without a prerequisite for, and therefore bias from, reference genome sequences from organisms expected to be present within any biological system [2, 3]. A metatranscriptomic approach designed without constraint to any a priori defined organism, but open to annotation from any sequenced strata of life, should be powerful in biological systems already recognised as highly complex, such as the human digestive tract or rhizosphere microbiome (although such microbiome complexity could arguably be defined by the current extent of study in a biological field). Here, the rhizospheric microbiome of *Salix purpurea* cv. ‘Fish Creek’ was challenged using hydrocarbon-contaminated soil and differential gene expression observed.

Pervasive organic pollutants, such as p*olycyclic aromatic hydrocarbons* (*PAHs*), polychlorinated biphenyls (PCBs) and C10-C50 petroleum hydrocarbons, represent serious risk to human health and the environment [4]. There are thought to be greater than 400,000 contaminated sites across North America [5, 6] and estimates are as high as 2.5 million sites across the EU [7]. Currently, rehabilitation of such sites is constrained by the high costs (> 2 M$/ha, [8]) of standard restoration strategies such as excavation and transport (dig-and-dump), with the consequence that these sites are rarely restored. In recent decades, a consensus has grown that environmentally sustainable and economically viable land restoration methods should be developed; phytoremediation is one such green technology alternative [9, 10]. Phytoremediation relies on the interaction between plants and their associated microorganisms to absorb, immobilise, volatilise, degrade, translocate or transform organic and inorganic contaminants [11]. In trials across Canada, a wide range of fast growing short rotation coppice willow cultivars (*Salix* spp.) have been shown as highly tolerant to *PAH*, PCBs and other organic petroleum hydrocarbon contaminants [12], as well as inorganic contaminants [13]. While the societal, environmental and economic benefits of rehabilitating these sites can be extensive (estimated within Canadian metropolitan areas at 4.6–7 billion dollars annually [14]), an additional benefit of the use of crops such as willow is that the biomass yielded per hectare can also be utilised for several valuable end-uses, including lignocellulosic biofuels [15], renewable electricity and heat generation [16], as well as green phytochemical production. Cultivation of biomass is often seen as the most substantial hurdle for economically feasible bioenergy production [17–19]. By aligning feedstock production strategy with land decontamination, cultivation can serve as a positive value-stream, in terms of the financial profit as well as a clear local environmental benefit [14].

To achieve effective success as a phytoremediation crop, willow is thought to maintain and exploit intimate symbiotic relationships with fungi. Roughly 6000 species of fungi from *Glomeromycota*, *Ascomycota*, and *Basidiomycota* have been categorised to date as mycorrhizal [20]. Recent research has explored the symbiotic interaction of arbuscular mycorrhizal fungi (AMF, endomycorrhizal fungi currently exclusively categorised within *Glomeromycota*) with willow [13, 21, 22] although trees and shrubs such as willow are characteristically known for interacting with ectomycorrhizal (ECM) fungi when mature [20, 23]. Such interactions are thought to be predicated on the exchange of nutrients from the fungi to the plant, in particular phosphate and nitrogen attained by the fungi from soils, and a highly controlled amount of sugars exchanged from the plant to fungi. However, the comprehensive identification of fungi from an extra-laboratory environment, let alone delineating their roles in a complex biological system, is confounded by culturing difficulty and that, of the estimated 1.5 million fungal species, less than 600 have been currently (2016) sequenced and annotated (JGI MycoCosm [24]).

The peril of confounding bacterial community assessment by culturing methodology alone is also widely acknowledged, but the progression of contemporary sequencing techniques and extensive research advancement driven by fields relating to the human microbiome has led to an understanding of the ubiquitous, exceedingly diverse, presence and involvement of bacteria in eukaryotic biology. The importance of bacteria to mycorrhizal fungi and/or plant health has been established even in conditions less challenging than anthropogenic soil contamination [25–28]. Bacteria whose presence and function is beneficial to mycorrhizal fungi and/or plants are often termed mycorrhizal helper bacteria MHB [29, 30] or plant growth-promoting bacteria (PGPB), such as the recently identified *Enterobacter* sp. 638, recognised as improving poplar growth on challenging marginal soils by up to 40% [31]. In relation to the rhizosphere, the highly complex environment has been very well reviewed by Bonfante et al. [20] as comprising ‘tripartite’ interactions between plants, mycorrhizal fungi and bacteria. The distinction between the potential roles of bacteria within the rhizosphere, in terms of the level of host interaction (rhizospheric, extracellular interacting or intracellular) and the spectrum of interaction type (pathogeny, symbiosis or commensalism), is problematic; however, metagenomics and metatranscriptomics can help to unravel this complexity by allowing gene function to be observed.

Here, all the RNA assembled de novo from roots of 12 willow trees, pot-grown in either contaminated or non-contaminated soil from a former petroleum refinery, was annotated and differential gene expression from any and all organisms identified was explored to see if the functionality of a successful phytoremediation system can be elucidated.

## Methods

### Contamination composition, experimental design and sampling

Both contaminated and non-contaminated soils were gathered from the site of a former petrochemical refinery at Varennes, Canada. Contaminated soil had an average C10-C50 petroleum hydrocarbon concentration of 912 mg kg^− 1^ (non-contaminated soil was below detection limit: < 100 mg kg^− 1^). *Salix purpurea* cv. ‘Fish Creek’ cuttings were established for 8 weeks in conventional potting media before being transferred to 20-l pots containing treatment soil within a larger experiment consisting of six blocks where randomised contamination effect was investigated (further soil and experimental information available from Yergeau et al. [27]). Growth conditions were 16 h 20 °C day and 8 h 18 °C night with excess watering and individual plant pot saucers to reduce leeching. After 6 months of growth, roots were harvested from six replicate trees per growth condition; soil was removed manually and roots samples were flash frozen in liquid nitrogen within 5 min of the initial perturbation.

### RNA extraction and Illumina sequencing

A modified CTAB protocol [32, 33] was used to extract RNA from roots with quality and quantity assessed using a BioAnalyser (Agilent, Mississauga, ON, Canada). Roots from 12 trees were sequenced: 6 from trees cultivated in contaminated soil and 6 from non-contaminated soil. Polyadenylated mRNA was amplified using Ambion’s MessageAmp™ II aRNA Amplification Kit. Amplified RNA was tested for genomic DNA content by PCR, using 18S rRNA gene primers and conditions described in Stewart et al. [34]. Indexing of cDNA samples for sequencing was performed in accordance with Meyer and Kircher [35]. The samples were sequenced (four separate runs) using an Illumina HiSeq 2500 platform.

### De novo metatranscriptome assembly and differential expression

Trimmomatic [36] was used to trim nucleotides of poor quality and reads < 40 bp were removed. Reads from all 12 biological samples were assembled into a de novo transcriptome using Trinity software set to default parameters [3]. Assembled contigs shorter than 200 bp were discarded. Bowtie2 [37, 38] was used to align the RNA-seq reads back to the de novo transcriptome with -a -X 600 parameters on top of default parameters. Low count contigs, below 29 total counts across all libraries, were filtered out (corresponding to those contigs below the 80th percentile [39]). Read back-mapping rates were an average of 64.91% across all 12 root samples. Raw and normalised transcript abundance (tpm) was calculated using eXpress [40] with default parameters. Differential expression (DE) was estimated using EBSeq based on median normalisation [41] and on an empirical Bayes model framework [42, 43], keeping contigs with a posterior probability of differential expression (PPDE) ≥ 0.95 (with target false discovery rate controlled at 5%). EdgeR [44] was used to generate MA plots based on trimmed mean of M-values normalisation method [45]. Extended quality control, assembly and normalisation information is provided in Additional file 1, and scripts are provided in Additional file 2.

### Annotation

The metatranscriptomic (*unconstrained*) annotation strategy, which queries a broad range of protein sequence repositories, was performed as outlined by Gonzalez et al. [46]. Briefly, the de novo assembled contigs were annotated using three major protein databases (nr NCBI non-redundant protein database, SwissProt and TrEMBL) as well as the *S*. *purpurea* 94006 reference genome. During the informed annotation step, *Populus trichocarpa* reference genome was also added. UniProt Archive (UniParc) database was used to protein blast differentially expressed (DE) contigs that did not have a hit in any of the databases. NCBI nucleotide database was used to nucleotide blast all DE contigs. *E* values < 10^−4^ (protein blast) and < 10^−6^ (nucleotide blast) were used as cutoffs. A previously reported method for selecting annotation (based on the percentage of maximum potential bitscore) from blastx returns was used to help select the primary annotation given multiple high scoring alignments for a single sequence, all statistically characterised as non-random [2]. BLAST hits that were not selected but have a high comparable percentage of maximum potential bitscore (within 10%) were retained for each contig as (alternative) secondary annotation. Custom scripting (in Python, R, Shell, Javascript) and Krona [47] were used to generate images and figures (Additional file 2).

### Transdecoder

To find coding regions within bacterial polycistronic sequences, we used TransDecoder software (https://transdecoder.github.io/). Protocol was followed according to the manual with default parameters. Precedence of transcriptional unit structure (putative operons) was verified in all cases against the database of prokaryotic operons (DOOR [48]) unless otherwise stated. A final hand curation step was included.

### Interrogation of unknown contigs

Further annotation was undergone for contigs with no hit on any database. Nucleotide blast was carried out using non-coding RNA database (NONCODE2016 from www.noncode.org), *Salix purpurea* 94006 genome, and NCBI ESTs database.

## Results and discussion

### Assembly, mapping and annotation

**Fig. 1:**
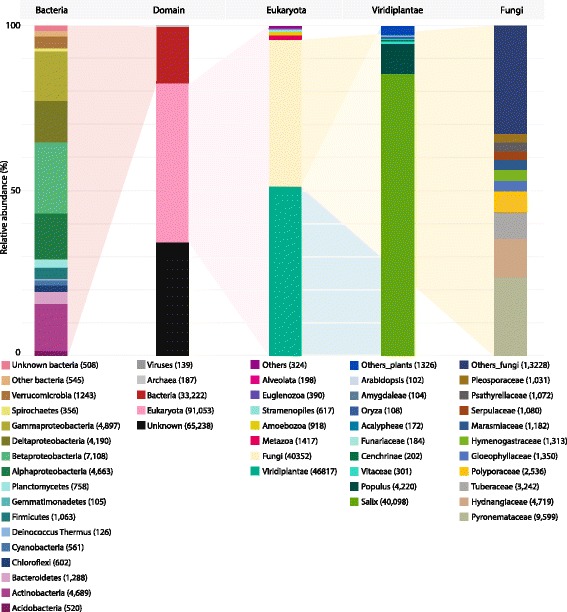
Total annotation. Annotation of the entire transcriptome assembly (including non-differentially expressed contigs). Bars representing Bacteria, Domain, Eukaryota, Viridiplantae and Fungi are selected as a useful overview of the diversity within the transcriptome. While bars represent data normalised to 100%, only ~ 65% of the sequenced reads were successfully mapped to the assembled transcriptome (so are overlooked here) and 34% of assembled contigs had no similarity to known sequences (so are again overlooked). Full annotation is provided in Additional file 4 and an interactive Krona of total annotation is available at: https://github.com/gonzalezem/Tripartite_Metatranscriptomics_article

A total of 189,849 confident contigs were confidently assembled de novo from 456,182,049 sequence reads. Overall, 125,151 contigs (66%) were confidently annotated as potential protein encoding transcripts, but 64,698 contigs (34%) had no protein homologue in SwissProt, Trembl or nr databases and are referred to as unknown henceforth (a substantial number of these were DE and investigated in greater detail in Additional files 1 and 3). The annotated transcripts included 91,053 Eukaryota (48%), 33,222 Bacteria (18%), 187 Archaea (0.01%) and 139 viral sequences (0.01%; including the common Illumina spike) (Fig. 1). Transcripts best annotated from eukaryotes were, as could be expected, mostly derived from Viridiplantae (46,817), although a close second was Fungi (40,352), with Metazoa (1417), Amoebozoa (918), Stramenopiles (617), Euglenozoa (390), Alveolata (198) and others (such as metagenomic or environmental samples) comprising only minor proportions of the transcript diversity (324).

### Total community makeup

Comparative multi-omics analysis by Hultman [49] and Tveit et al. [50] has revealed some shortfalls in community assessment using internal transcribed spacer (ITS) and 16S ribosomal RNA (16S rRNA), even at the phylum level (discussed with additional *Basidiomycota* identification investigations in Additional file 1). Others have expressed even stronger scepticism, such as Bent et al. [51], who discussed the difficulties in providing reliable diversity indices from microbial fingerprinting methodology [52–57]. An alternative, top-down approach is employed here which accepts uncertainty of the microbiome community, assembling as many contigs as possible within the system and performing differential expression analysis on all retained contigs without any necessity for a priori sequence information before *independent* annotation using the world’s major protein repositories.

Transcripts clearly identified as deriving from *Salix* (the mass majority from the *Salix purpurea* 94006 genome) made up 85.6% (40,098) of the contigs from plant species. While this was expected, it is perhaps more informative to recognise that 14.4% of plant transcripts were best annotated outside of *Salix*, mostly from the very close relative *Populus* (9.0%) but also from other genera (*Vitaceae* 0.6%; *Cenchrinae* 0.4%; *Funariaceae* 0.4; *Acalypheae* 0.4%; *Oryza* 0.2%; *Amygdaleae* 0.2%; *Arabidopsis* 0.2% and others 2.8%). This is roughly in line with previous research suggesting between 6 and 10% of *Salix purpurea* genes can be better identified by querying the broader protein repositories [2, 46] and that, importantly, over 10% of likely *Salix* gene expression can be obscured by only mapping to a reference genome, even when species specific.

Extraordinary fungal diversity was found within the transcriptome. Although RNA was extracted from washed roots, this diversity is not surprising as the majority of plant species are thought to employ (widely diverse) below-ground fungal association in order to survive and compete in the biosphere [12, 58]. De novo sequencing provides a unique opportunity to observe such opaque below-ground genetics (Fig. 1, only fungal genera with over 1000 hits are presented, comprehensive annotation is provided in Additional file 4 and interactive Krona graphs). The fungal community comprised 40,352 distinct contigs by primary annotation, the majority were *Pyronemataceae* (23.8%), then *Hydnangiaceae* (11.7%), *Tuberaceae* (8.0%), *Polyporaceae* (6.3%), *Gloeophyllaceae* (3.3%), *Hymenogastraceae* (3.3%), *Marasmiaceae* (2.9%), *Serpulaceae* (2.7%), *Psathyrellaceae* (2.7%) and *Pleosporaceae* (2.6%) contigs as well as a very extensive quantity of others (13,228 contigs or 32.8%).

Bacterial diversity was extremely high, even at class level, with the 33,222 transcripts originating from *Betaproteobacteria* (21.4%), *Gammaproteobacteria* (14.7%), *Actinobacteria* (14.1%), *Alphaproteobacteria* (14.0%), *Deltaproteobacteria* (12.6%), *Bacteroidetes* (3.9%), *Verrucomicrobia* (3.7%) and *Firmicutes* (3.2%) as well as from other genera (*Planctomycetes* 2.3%; *Chloroflexi* 1.8%; *Cyanobacteria* 1.7%; *Acidobacteria* 1.6%; uncharacterised bacteria 1.5%; *Spirochaetes* 1.1%; *Deinococcus-Thermus* 0.4%; *Gemmatimonadetes* 0.3%; others 1.6%). These bacterial contigs assembled from RNA extracted from roots very closely reflected the community reported in a previous experiment performed by Yergeau et al. [59] using 16S rRNA sequencing, where rhizospheric bacteria (from soil attached to roots), in association with the same willow cultivar grown in the same contamination, was also dominated by *Betaproteobacteria* (~ 39%) and *Gammaproteobacteria* (~ 38%). The authors used 16S rRNA sequencing to positively identify a wide spectrum of bacteria at the class level; while the methodology is not quantitatively comparable to that used here (Additional file 1), the proportions of the community makeup were roughly similar at a phylum and class level.

### Differentially expressed contigs

#### Metaorganism patterning in gene expression

**Fig. 2:**
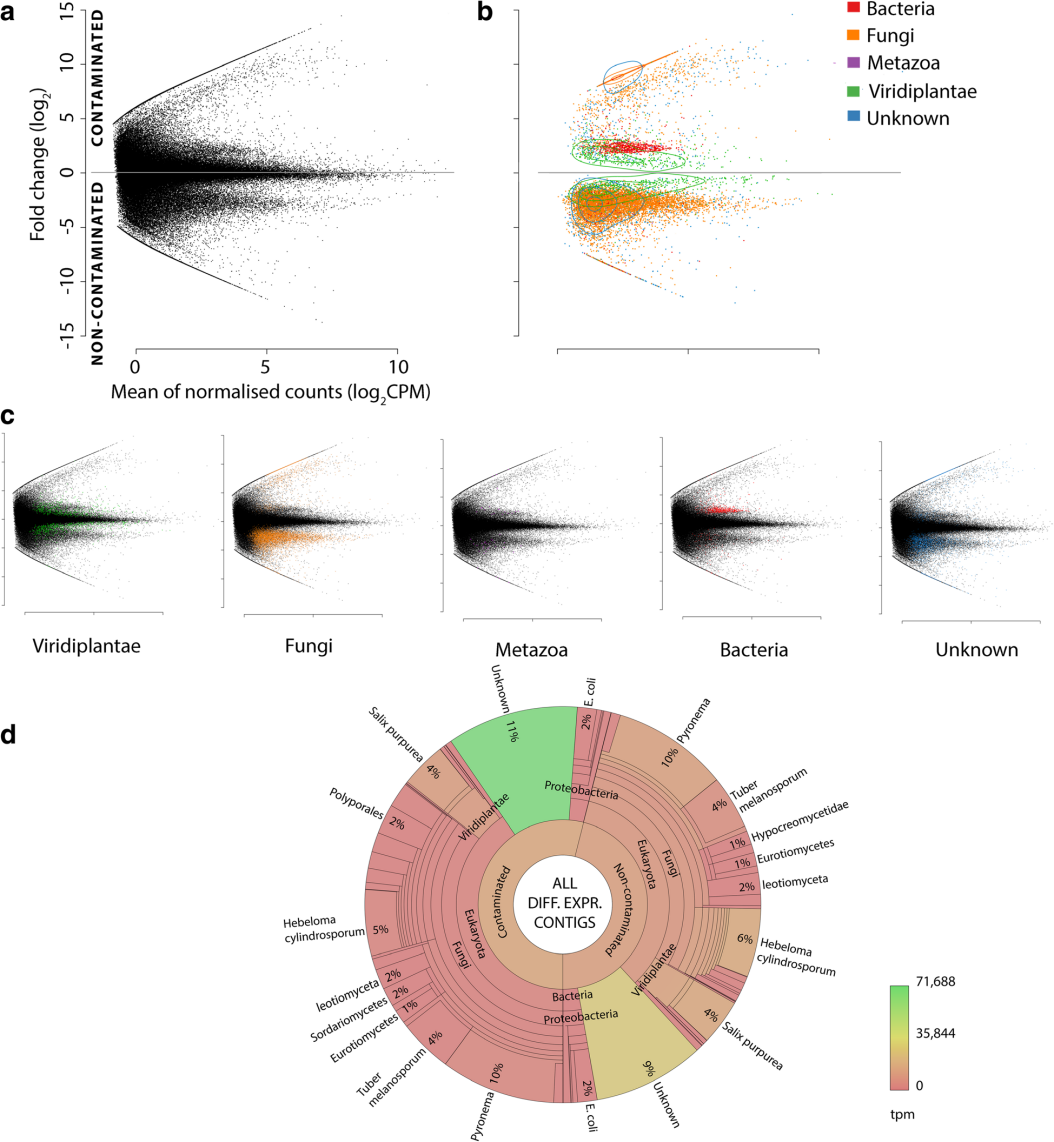
Origin of differentially expressed contigs. MA plots (**a**–**c**) of de novo assembled transcriptome; *y*-axis represents fold change (FC, log_2_) between contaminated (+ive) to non-contaminated conditions (−ive), and the *x*-axis represents mean normalised (EdgeR) counts per million (log_2_ CPM). Plot **a** all contigs (including non-DE) and **b** DE contigs only; coloured by annotation including contours to represent contig density relative within each group. **c** Individual MA plots of differentially expressed (DE) contigs annotated from Viridiplantae, Fungi, Metazoa, Bacteria and Unknown (no known similar sequences) are included for clarity. Data patterning from contamination-driven shifts in the community are observable (**a**) prior to any annotation. An epsilon factor is added in place of zero abundance where contigs are present in only one condition to allow visualisation and abundance comparison (as fold change would be infinite); the presence or absence of contigs (due to contamination) is biologically informative. **d** All DE contigs represented within a Krona graph [47]; the proportion of each taxonomic grouping is defined by the number of distinct contigs, whereas the colour represents the relative abundance (transcripts per million, tpm) of transcripts in each taxon. An interactive Krona graph to assist navigation of DE contig annotation origin is available at: https://github.com/gonzalezem/Tripartite_Metatranscriptomics_article. A full contig list including expression information, annotation (1° and 2°) and gene ontology is provided in Additional file 4 whereas DE only contigs are provided in Additional file 9

A total of 12,576 contigs were identified as DE between non-contaminated and contaminated soil conditions (Fig. 2). DE contigs (putative transcripts when annotated as protein encoding) may be responding directly to hydrocarbon toxicity or to environmental alterations due to the high soil concentrations of petroleum hydrocarbons, such as nutrient or water availability. Beyond this, differential expression could also be due to secondary responses representing the shift in the microbiome community or, indeed, expression of other organisms independent of the shift in community within the metaorganism. No sequences were discarded based on annotation, especially since previous studies have demonstrated the potential for the removal of sequences (unexpected a priori) to biologically [60] and technically [2] confound data integrity and interpretation. The necessity for altering the paradigm of experimental approaches in light of a modern understanding of metaorganismal complexity has recently been recognised as essential in mammals with respect to phenotypic assessment incorporating the microbiome [1]. In particular, bacterial DE sequences were retained for analysis (despite polyA enrichment prior to RNA-seq).

A substantial proportion of all the contigs assembled (including non-DE) were unknown (34%), bearing no confident homologous sequence in the major protein repositories. Although unknown sequences are often discarded, the strategy reported here very deliberately maintains data where possible due to the potential for unknown DE sequences to represent novel organisms and functionality. Over 20% of the total DE sequences were indeed unknown (Additional file 3); extended investigation into these sequences is discussed in Additional file 1.

Pooling of highly complex gene expression data from non-model plants into ontology groups can give the impression of an overview of the biological system (GO terms are provided for query Additional file 4); however, an attempt to explore DE function at a transcript level across all the organisms within the samples is presented here.

#### *Salix purpurea*

**Fig. 3:**
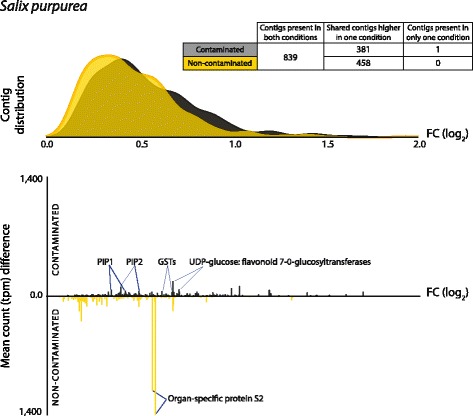
*Salix purpurea* differential expression (DE) transcript distribution and abundance (transcripts per million, tpm) weighted fold change (log_2_). Top: fold change (FC log_2_) distribution of DE genes contaminated (black) and non-contaminated (gold). Bottom: mean transcript counts (tpm) difference between conditions against fold change per DE contig. The highly abundant transcripts discussed within the text are labelled. A full DE transcript list including expression data, functional description (if available), gene ontology terms (if available) and secondary annotation (if available) is provided in Additional file 5. PIP plasma membrane intrinsic protein (aquaporin), GST glutathione S-transferase

A total of 839 DE contigs were annotated as *Salix purpurea*, representing 6.7% of all DE contigs from roots. The number of contigs up (45%) or down-regulated (55%) was similar in response to contamination (Figs. 2 and 3). Despite the strain/cultivar studied here (*S*. *purpurea* ‘Fish Creek’) being the same species as that of the sequenced reference genome, *S*. *purpurea* 94006, 131 contigs (which we presume originate from *Salix*) were better annotated from other plant species. This is consistent with another study suggesting (top-down) de novo strategies can capture more sequence variation than mapping to a single non-clonal reference genome alone [2]. Although the diversity of *Salix* DE contigs was relatively low within the system, with the exception of the unknown, they had the highest relative abundance (analysed as transcripts per million, TPM); perhaps unsurprisingly, given RNA was sampled from washed *Salix* roots.

##### Direct detoxification/stress responses

The expectations for plant gene expression within a high hydrocarbon-contaminated environment would include an increase in cytochrome P450 monooxygenases. Out of 26 DE transcripts identified as encoding cytochrome P450 family proteins, 18 were in higher abundance within control roots (cultivated in non-contaminated soil) (Additional file 5). Only two were annotated as putative monooxygenases (c585325_g1_i1 and c601406_g5_i2), with one upregulated and one downregulated with respect to contamination. Another expected set of stress-induced detoxification equipment, known to be transcript abundance dependent, are glutathione S-transferases (GSTs), which catalyse glutathione conjugation to a broad range of xenobiotics in order to facilitate vacuolar loading (compartmentalisation of cellular pollutants). Only a single GST was downregulated in roots from contaminated soil, with 13 upregulated (8 *tau* class, including GSTU19, 37, 48 and 50) at consistently high relative abundance levels (tpm) (Fig. 2 and Additional file 5).

UDP-glucose:flavonoid 7-O-glucosyltransferase was the most abundant upregulated *Salix* gene in contaminated roots (c600230_g5_i1; 216.94 tpm), and two other putative isoforms were also highly upregulated (c600230_g5_i2, 90.71 tpm; c600230_g6_i1, 52.53 tpm; Fig. 2).

Such increases in flavonoid glucosyltransferase expression represent a common conjugation mechanism in plant detoxification metabolism [61]. Alternatively, UDP-glucose:flavonoid 7-O-glucosyltransferase, which allows symbiotic interaction with microorganisms in *Glycine max* [62], can be limiting for vacuolar loading of the isoflavone conjugates, and the corresponding conjugate-hydrolysing β-glucosidase is localised to the plant root apoplast, intriguing in terms of the extensive interaction with microbes evidently ongoing within the roots.

Apart from the UDP-glucose:flavonoid 7-O-glucosyltransferase and glutathione transferases, the most highly expressed DE transcripts in contaminated roots were aquaporins and dehydrin. This could be expected given petroleum hydrocarbon contamination is likely to reduce access to water and thus induce osmotic stress in roots. Three PIP1.1 transcripts (c483320_g1_i2, 125.11 tpm; c589371_g5_i4, 88.10 tpm; c589371_g5_i8, 30.63 tpm), three PIP2.1 (c599604_g6_i1, 79.73 tpm; c576656_g7_i3, 70.73 tpm; c599604_g8_i6, 4.23) and PIP2.7 (c602203_g2_i9, 21.70 tpm) were all highly abundant in contaminated conditions. Beyond a direct plant response to soil conditions, it has long been known that root-associated fungi, particularly ECM fungi, can improve not only nutrient uptake in trees, but also water [63–65]. It is interesting to note, in light of subsequent fungal gene expression, that extreme PIP upregulation has previously been observed as induced by the ECM fungi *Amanita muscaria* in fine ectomycorrhized roots of poplar (a close relative of *Salix*, sharing macrosynteny) [66] as well as in *Salix* by the AMF *Rhizophagus irregularis* [22].

##### Nitrogen and carbohydrate transport

Five amino acid transporters were DE, two in higher abundance in non-contaminated trees and three in contaminated trees. Two were distinct cationic amino acid transporters, one in higher abundance in non-contaminated conditions (c598534_g2_i3, 6.65 tpm) and one higher in contaminated conditions (c584679_g1, 18.25 tpm). Another of the transcripts higher in contaminated conditions (c594518_g1_i1) was most similar to AAP6 in poplar (87% identity, blastn *e*-value = 7e^-29^), and the two remaining transporters (one in higher abundance in each condition) were both similar to vacuolar amino acid transporter 1. AAP6, an acidic and neutral amino acid transporter known to be expressed in roots, is distinctive in having high substrate affinity (so could potentially be relevant to low amino acid concentration uptake) as well as having an affinity for aspartate [67]. Seven nitrate/peptide transporters where DE with six in higher abundance in contaminated conditions, sharing close homology with poplar NPF (NTR/PTR family) 5.4, 3.1 and 5.2 proteins. Alongside PIP expression, these are hallmarks of root symbiosis [68] in terms of differential expression and are thought to represent oligopeptide import of AM- and ECM-packaged nitrogen, the principal fungi to plant facet of resource exchange.

In terms of potential concomitant plant to fungi exchange of resources, differential expression of a putative sucrose transporter (c582330_g6_i4) with high abundance in roots from contaminated soil was identified, which would be expected if increased ECM interaction were underway in these conditions. Three distinct trehalose-6-phosphate synthase transcripts were DE (c598745_g5_i1, c536396_g3_i1, c599008_g3_i2), all in higher abundance in roots cultivated in contaminate soil. While the role of trehalose synthesis in plants is somewhat obscure [69], trehalose is the dominant storage carbohydrate in AMF hyphae and implicated in ECM abiotic stress tolerance [70, 71], so expression here is intriguing in light of concomitant fungal DE. Additionally, the sugar transporter SWEET2 (c555872_g1_i5) was in higher abundance in roots from contaminated soil. SWEETs (Sugars Will Eventually Be Exported Transporter) have only been identified relatively recently [72] but have already been strongly implicated in plant-fungal interactions [73], including *Solanum tuberosum* roots colonised by AMF [74]. In *Arabidopsis*, SWEET2 has been shown to accumulate in root hairs, cap and epidermis, and in cells in close contact with the rhizosphere [75]. Interestingly, the authors suggest that SWEET2 functions as a bidirectional glucose transporter that could control glucose secretion through limitation/vacuolar loading as part of highly complex and co-ordinated interactions with rhizospheric microbes.

##### Community association

The most abundant downregulated contigs in *Salix* roots from non-contaminated soil encoded two distinct organ-specific S2 proteins (Fig. 2).

While these proteins are of unknown function, they have been found to be downregulated in a *Medicago truncatula* 1-deoxy-d-xylulose 5-phosphate synthase 2 (catalysing the beginning of the MEP pathway) knockdown, which also reduced AMF colonisation of roots [76]. An acidic endochitinase (EC 3.2.1.14, c583738_g7_i2) was upregulated in contamination-treated roots. While this would intuitively be associated with plant defence against pathogenic fungi, a number of studies have found that this endochitinase can encourage symbiotic association with ECM fungi, such as *Hebeloma*, *Suillus*, *Wilcoxina*, *Pisolithus*, *Paxillus* and *Amanita* with spruce, birch and *Eucalyptus*; the suggested mode of action being that non-fungi-damaging apoplastic chitinase modifies ectomycorrhizal elicitors to facilitate symbiotic interaction [77–80]. When considered alongside the preceding data, *Salix* root DE reflects plant recognition of foreign molecular patterns [81], the non-recognition impact of effectors [82] as well as alterations in nutrient exchange and water availability known to be drastically altered by fungal interactions [65].

#### Fungi

##### Division by taxonomy

**Fig. 4:**
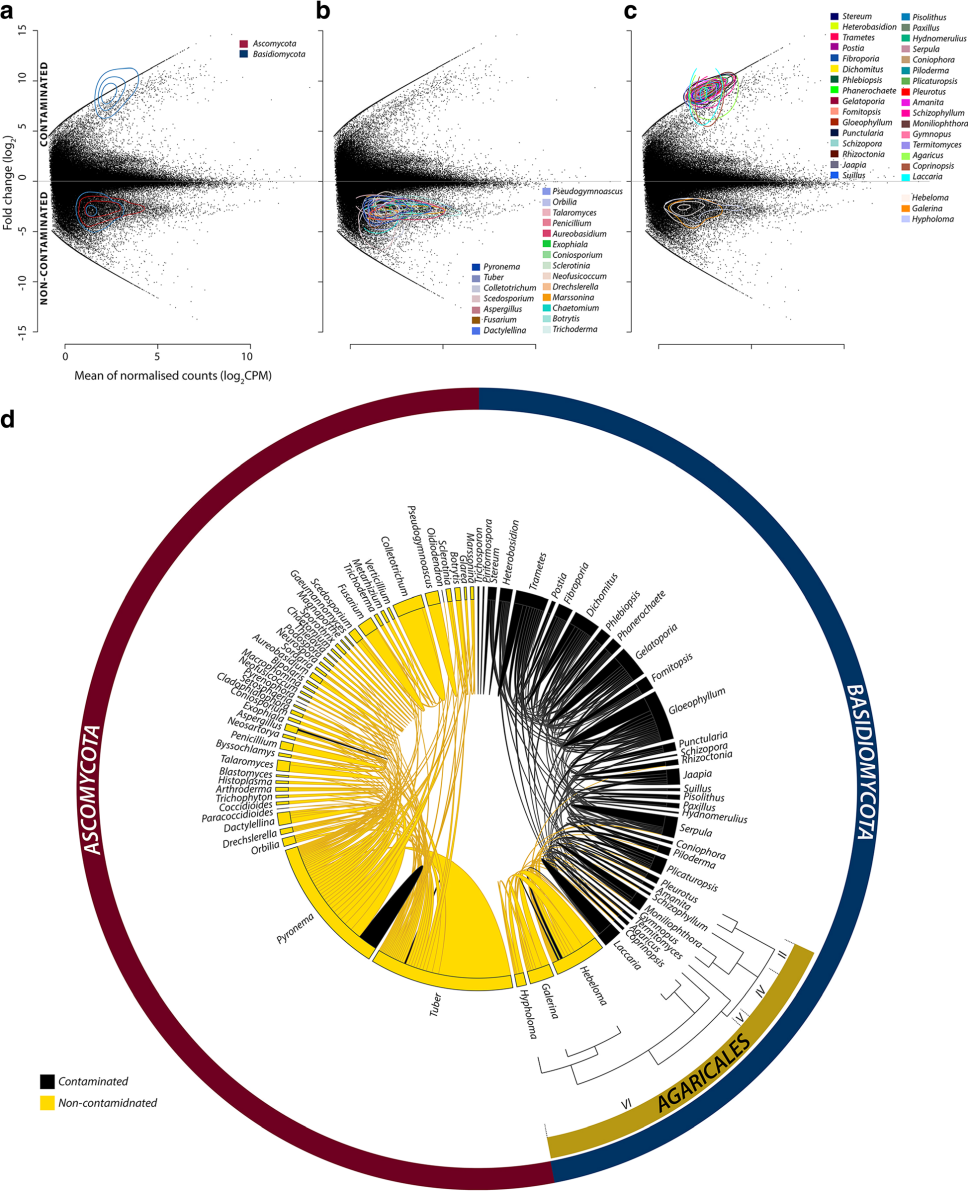
Taxonomy of fungal differential expression and secondary annotation. MA plots of de novo assembled transcriptome; *y*-axis represents fold change (FC, log_2_) between contaminated (+ive) to non-contaminated conditions (−ive), and the *x*-axis represents mean normalised (EdgeR) counts per million (log_2_ CPM). Contours representing relative DE contig density. **a** DE contigs annotated from fungi with *Ascomycota* (red) and *Basidiomycota* (blue), **b** DE *Ascomycota* contigs with genera annotating > 20 contigs highlighted and **c** DE *Basidiomycota* contigs with genera annotating > 20 contigs highlighted. **d** Secondary annotation of each DE fungal contig illustrating alternative, equally valid annotation [2] from other species (presented as genera for clarity). Genera with correspondences > 20 are presented and coloured by DE direction (more abundant in contaminated roots = black; more abundant in non-contaminated roots = gold). *Agaricles* phylogeny (an order of *Agaricomycetes*) is provided to visualise expression profiles against relatedness, with clade II (*Pluteoid*), IV (*Marasmoid*), V (*Tricholomatoid*) and VI (*Agaricoid*) structure (taken from Matheny et al. [86]). An interactive chord diagram and Krona graph to assist more comprehensive navigation of taxonomy and fungal secondary annotation are available at: https://github.com/gonzalezem/Tripartite_Metatranscriptomics_article. A full fungal DE contig list including expression information, annotation (1° and 2°) and gene ontology is provided in Additional file 6 whereas a full list of *Basidiomycota* DE contigs upregulated in roots of contaminated trees is provided in Additional file 7

**Fig. 5:**
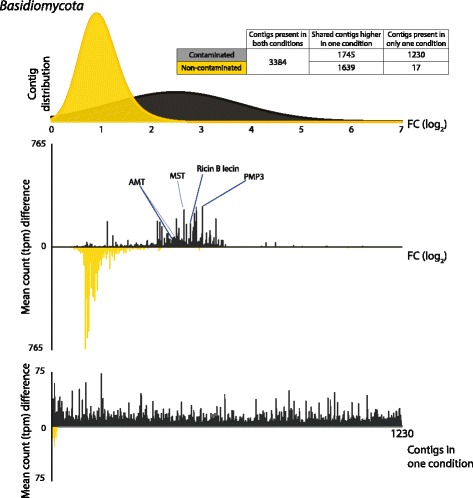
*Basidiomycota* differential expression (DE) transcript distribution, abundance (transcripts per million, tpm) weighted fold change (log_2_) and contigs present in only one condition. Top: fold change (FC log_2_) distribution of DE genes contaminated (black) and non-contaminated (gold). Middle: mean counts (tpm) difference between conditions against fold change per DE contig. The highly abundant transcripts discussed within the text are labelled. A full DE transcript list including expression data, functional description (if available), gene ontology terms (if available) and secondary annotation (if available) is provided in Additional file 7. MST monosaccharide transporter, AMT ammonium transporter, PMP3 plasma membrane proteolipid 3. Bottom: contigs present in only one condition (termed infinity genes in Additional files)

Fungi represented the kingdom with the most diverse genetic response to contamination within the root samples, being the annotation source of the 8252 distinct DE contigs. The paradigm of direct up vs down-regulation collapses when multiple organisms and natural biological complexity are acknowledged in sequencing studies (when the dynamic nature of the metagenome is considered [2]), as the presence of an organism can vary as well as gene expression within organisms. Therefore, DE contigs can represent direct or indirect responses to treatment within a given organism of stable presence (with respect to treatment), including responses to highly diverse, changeable and potentially hostile biological environment, but also represent baseline transcription and metabolism of newly present, absent, growing or diminishing organisms within the system. The contigs annotated as fungal had very distinct patterning in relation to contamination; of the contigs *present* in both conditions, 88.8% (6184 contigs) had higher abundance in non-contaminated trees while, in contrast to this, of the contigs *only present* in trees of one condition, 96.3% (1239 contigs) were in contaminated conditions (Figs. 2b, c; 4a and 5). These expression patterns, even before investigating gene function, can be interpreted as a general downregulation of constitutive fungal expression due to contamination and the arrival of a distinct, contamination specific, set of expressing genes potentially representing a shift in community makeup. By comparison, only seven *Salix* DE contigs were expressed in only trees of a single condition.

The two *Ascomycota* species most represented within DE transcripts were the closely related *Pyronema omphalodes* (a saprophyte) and *Tuber melanosporum* (an ECM fungi) (Fig. 4b and Additional file 6). These species both dominated fungal differential expression and had high enough levels of primary annotated contigs (2303 contigs and 1077 contigs) to merit very confident identification of these species, or of close relatives, within the system. Transcript levels were almost all more abundant within roots of non-contaminated trees (94.1 and 98.4% for *P*. *omphalodes* and *T*. *melanosporum*, respectively). While an alteration in gene expression in response to hydrocarbon conditions is possible, the extremity of polar expression might also suggest a lower abundance for these organisms due to the impact of contamination stress and/or increased competition from more contamination-tolerant life forms. *Tuber* has been previously identified as associated with *Salix* [83, 84]; non-constitutively expressed ECM high-abundance markers were DE in *Tuber* and were indeed in higher abundance in non-contaminated conditions, such as RAS protein (c594475_g3_i1) [85], implying successful *Tuber* ECM interaction was underway in non-contaminated conditions but was suppressed by hydrocarbon contamination.

Unlike *Ascomycota*, *Basidiomycota* expression was dynamic with respect to contamination, as 1639 contigs annotated from three closely related species (from the *Agaricoid* family’s *Hymenogastraceae* and *Strophariaceae* [86]) *Heboloma cylindrosporum* (an ECM fungi), *Galerina marginata* (predominantly white rot) and *Hypholoma sublateritium* (white rot) were downregulated, while 1745 contigs, with a highly distinctive expression pattern, were upregulated but broadly annotated from 61 genera (Fig. 4c and Additional files 6 and 7). These upregulated contigs were distinctive in the extreme diversity of annotation origin and as the majority (71%) were present *only* in roots from contaminated soil. To investigate the origin of these phytoremediation responsive contigs, and provide additional evidence towards their species of origin, we explored the species diversity of equally good hits using ‘secondary annotation’ [2] (Fig. 4d; Additional file 1) as well as using nucleotide blastn (a strict blastn reduces observation of fungal data by 99.5%, Additional file 8).

##### Secondary annotation

By utilising secondary annotation, the retention of equally (statistically) ‘good’ annotation hits, all 12,576 DE contigs can be more confidently assigned to an organism of origin by elucidating any ambiguity in annotation which is often overlooked. The set of 1745 *Basidiomycota* genes most often had secondary annotation from known ECM (such as *Scleroderma citrinum* and *Paxillus involutus*) and saprophytes (such as *Pleurotus ostreatus* and *Trametes versicolor*), in agreement with the current awareness that the continuum of mutualism is complex [87]. Very little crossover was observed between *Basidomycota* and *Ascomycota* in terms of differential expression due to contamination response, as *Basidomycota* extensively dominated the increased expression in response to contamination conditions (Fig. 4a–d). Secondary annotation provides an additional benefit in terms of mining useful functional description from across the world’s major data repositories (Additional files 2, 3, 4, 5, 6, 7, 8 and 9), but it is also interesting to note that retention of secondary annotation independently reflects classical *Basidiomycota* phylogeny ([86]; Fig. 4d) (albeit with a crude thresholds limiting data retention to only the very closest homologues), particularly interesting is if this idea is considered in the context of the 189,839 total annotated contigs that include 491,505 secondary annotation (Additional file 4).

An important take home message here, when querying unknown sequences using BLAST, is the benefit of not taking the single top returns as fact. Pertsemlidis and Fondon [88] have detailed how differentiating proteins of high homology from BLAST scoring system should be performed with caution and others have outlined the extensive risk of confounding biological interpretation by not acknowledging the uncertainty of annotation [2]. Secondary annotation of *Basidiomycota* was spread widely across *Agaricomycotina* for upregulated contigs whereas downregulated contigs (> 95%) derived from a specific *Agaricoid* clade comprising the three closely related genera (*Hypholoma*, *Galerina* and *Hebeloma*) (Fig. 4c) which, understandably, shared the majority of secondary annotation with each other (Fig. 4d). For the distinct, upregulated *Basidiomycota*, secondary annotation revealed no dominant source of annotation (homology) to our contigs within *Agaricomycotina* (more detailed sequence origin investigation is presented in Additional files 1 and 8).

##### Upregulated *Basidiomycota* function

Given that the intricacy, or perhaps futility, in differentiating between saprophytes and ECM fungi has been well discussed [89, 90], determining the fungal mode of action from expression study is problematic [91] and is further confounded by the generally accepted belief of multiple ECM evolutionary events in *Basidiomycota* ([92]). The scale of mycorrhizospheric complexity is very high; once DE genes have been sub-selected as best annotated from fungi (keeping in mind that 2575 DE contigs were unknown, having no known sequence similarity), further sub-selected based on *Basidiomycota* annotation and even further sub-selected based on response to contamination (just those contigs in higher abundance in contaminated conditions), 1745 DE-annotated transcripts remain to describe potential functionality (driven by positive expression) within the system. Of these 1745 DE transcripts, 70% (1227 contigs) had unknown function and were annotated as hypothetical, predicted, putative or uncharacterised proteins from *Basidiomycota* species. These poor functional description terms are selected against during annotation if an equally good hit is available in secondary annotation, in practice re-mining any confident functional descriptions available within the major protein data repositories.

In terms of recognisable gene function, the most abundant fungal contig expressed in contaminated conditions was the relatively cryptic plasma membrane proteolipid 3 (Pmp3), best annotated from *Moniliophthora*, with 301.42 tpm (c553133_g5_i1, 2128.20 FC) (Additional file 7). The small hydrophobic Pmp3 (dissimilar to recognised hydrophobin structure) has been characterised as highly conserved in fungi, is environmental stress induced (cryptic in yeast under standard laboratory conditions), involved in cytotoxic cation tolerance [93] and sphingolipid synthesis [94] so could be related to membrane integrity maintenance given the challenge of contamination conditions. Generally, the most highly expressed contigs within the group of distinctive *Basidiomycota* (Figs. 4c and 5) were not putatively related to hydrocarbon degradation but, instead, were related to classical association with plant roots and/or the highly upregulated bacteria (*Enterobacteriaceae* sp.), namely carbohydrate import, nitrogen (nitrate, ammonia and amino acid) metabolism and transport, and bacterial interaction. This could be expected in light of the hydrocarbon degradation mechanisms DE in the *Enterobacteriaceae* sp. (outlined below) if the interaction did indeed involve some degree of tripartite mutualism.

*Putative hydrocarbon degradation*

Surprisingly none of the expected hydrocarbon degrading monooxygenases were identified within the 1745 DE *Basidiomycota* contigs which increased in abundance due to contamination (Fig. 5, Additional file 7). Only three dioxygenase encoding contigs were identified as DE, all of which had relatively low abundance levels. Two were poorly characterised dioxygenase family proteins (c596412_g1_i1, 8.78 tpm and c593762_g1_i1, 5.93 tpm), but one was a putative extradiol aromatic ring-opening dioxygenase (c598058_g2_i2, 4.23 tpm), functionality well recognised in bacterial PAH degradation studies [95–97] but less familiar in fungi. No lignin peroxidases, manganase-dependent peroxidases (class II peroxidases) or laccases (with potential degradation functionality [26]) were identified as DE, expected if a ligninolytic (white rot) mode of action was underway, particularly as the associated enzyme secretion is highly dependent on transcript levels [91]. Two DE contigs, however, did encode glutathione peroxidase-like proteins (c591991_g1_i5, 8.78 tpm; c591991_g1_i10, 2.14 tpm), and one encoded a thioredoxin-dependent peroxidase (c565655_g1_i1, 48.94 tpm) were upregulated, although this is a common response to contamination-induced oxidative stress [98].

*Carbohydrate transport and CAZy*

One of most intuitive approaches to distinguish between saprophitic and ECM ecological strategies (the most likely in *Basidiomycota* here) would be to compare the expression of genes for plant cell wall degradation and carbohydrate import mechanisms, although this is a non-trivial task [91]. For instance, the molecular mechanisms underpinning much of the carbon transfer to ECM fungi from plants is unclear; while there is strong evidence that up to 30% of the plant’s total photoassimilates can be transferred to ECM fungi [99], few hexose transporters have been experimentally validated. The monosaccharide transporter MST1 in both *Amanita muscaria* (fourfold upregulation during symbiosis [100]) and *Laccaria bicolor* [101] being the exception to this.

The third most abundant (275.43 tpm, Table 1) *Basidiomycota* DE contig positively responding to contamination was a probable monosaccharide transporter (c601571_g1_i1; equally well annotated from either *Serendipita vermifera* or *Piriformospora indica*, Additional file 7), as well as two similarly annotated putative isoforms (25.71 tpm and 18.19 tpm) (Fig. 5 and Table 1). This is especially noteworthy as Hynson et al. [102] detected potential ECM lineages within *Serendipitaceae* and because *P*. *indica* has been shown to stimulate plant growth, but the mechanics of the symbiosis is somewhat cryptic (and can even cause plant cell death [103]). Interestingly, Zuccaro et al. [104] also found that this monosaccharide transporter was highly upregulated in *Piriformospora indica* and identified expression as clearly associated with barley root biotrophism (as opposed to saprotropism). The *Saccharomyces cerevisiae* homologue (*e*-value = 5e−17, 67% identity) of this MST, the extracellular glucose sensor rgt2, is well studied as also having sensor functionality [99, 105]; however, given the extremely high relative abundance, we would speculate high glucose import function as more likely to drive differential expression (due to increased transcript dependency of function). This probable monosaccharide transporter (c601571_g1_i1) is also similar to the abovementioned *Laccaria bicolor* MST1.3 (monosaccharide importer CAQ53118.1; lacbi1:301992, blastx *e*-value = 2e^−21^, 48% identity), whose crucial role in ECM glucose import is further supported by ^14^C-labelled glucose trials which demonstrated not only strongly upregulation during ECM formation when compared to extraradical mycelium expression but also strong evidence of substantial glucose import functionality [101]. Interestingly, in *Laccaria bicolor*, glucose uptake by MST1.3 was only very slightly affected by the presence of fructose (whereas uptake was inhibited by fructose in others, allowing for the possibility of sucrose hydrolysis at the rhizospheric interface). Alongside potential plant cell wall binding machinery, glucose import represents a highly expressed, recognisable function within the *Basidomycota* transcriptionally responding to contamination conditions and seems a credible candidate describing the currency of plant to fungi symbiosis with *Salix* roots.

**Table 1:** Fungal carbohydrate metabolism and CAZy. EBSeq [42, 43] was used to estimate posterior probability of differential expression (PPDE) ≥ 0.95. A full DE transcript list including expression data, functional description (if available), gene ontology terms (if available) and secondary annotation (if available) is provided in Additional file 6

Monosaccharide transporters					
Query id	Cont mean tpm	Treat mean tpm	FC	1° annotation id	Subject description
c601571_g1_i1	0.33	275.43	830.24	gi|751683823|gb|KIM33975.1|	Monosaccharide transporter (MST)
c601571_g1_i3	0.00	25.71	High	gi|751683823|gb|KIM33975.1|	Monosaccharide transporter (MST)
c601571_g1_i7	0.00	18.19	inf	gi|749760949|gb|KII85851.1|	Monosaccharide transporter (MST)
CAZy					
c601768_g1_i4	0.01	12.09	891.69	tr|A8N526|A8N526_COPC7	Exo-beta-1,3-glucanase (GH5)
c601246_g1_i2	0.00	9.29	inf	tr|S7QIL0|S7QIL0_GLOTA	Glycoside hydrolase (GH13)
c601246_g1_i3	0.00	8.75	inf	tr|S7QIL0|S7QIL0_GLOTA	Glycoside hydrolase (GH13)
c601246_g1_i1	0.00	3.40	inf	tr|S7QIL0|S7QIL0_GLOTA	Glycoside hydrolase (GH13)
c598537_g2_i2	0.00	20.09	inf	gi|695542974|ref.|XP_009543687.1|	Glycoside hydrolase family 13 protein
c600093_g2_i1	0.00	2.50	inf	gi|646308860|gb|KDQ30003.1|	Glycoside hydrolase family 13 protein
c598962_g2_i1	0.00	4.02	inf	gi|691791220|emb|CDO73299.1|	Glycoside Hydrolase Family 13 protein
c546152_g1_i1	0.00	7.98	inf	tr|F8PDS7|F8PDS7_SERL9	Glycoside hydrolase family 13/GT5 protein
c594633_g1_i2	0.11	50.90	449.23	gi|751693072|gb|KIM43056.1|	Glycoside hydrolase family 131 protein
c569099_g1_i1	0.00	6.22	inf	gi|595767974|ref.|XP_007262530.1|	Glycoside hydrolase family 16 protein
c601155_g2_i1	0.00	3.25	inf	gi|751697951|gb|KIM47928.1|	Glycoside hydrolase family 16 protein
c601155_g2_i2	0.00	9.44	inf	gi|751718111|gb|KIM67108.1|	Glycoside hydrolase family 16 protein
c601339_g1_i1	0.00	8.66	inf	tr|M2QB49|M2QB49_CERS8	Glycoside hydrolase family 3 protein
c594608_g1_i1	0.00	4.13	inf	tr|S7Q7X9|S7Q7X9_GLOTA	Glycoside hydrolase family 38 protein
c601768_g1_i2	0.00	4.18	High	gi|749771114|gb|KII95444.1|	Glycoside hydrolase family 5 protein
c601768_g1_i3	0.22	2.91	13.51	gi|749771114|gb|KII95444.1|	Glycoside hydrolase family 5 protein
c598880_g1_i5	0.04	104.28	2617.19	gi|660966484|gb|KEP50967.1|	Barwin-like endoglucanase GH45
c600699_g2_i2	0.00	5.19	inf	gi|761954826|gb|KIY74457.1|	Glycoside hydrolase family 45 protein
c600240_g5_i1	0.04	37.05	862.09	gi|597903192|ref|XP_007298895.1|	Glycoside hydrolase family 72 protein
c597573_g1_i2	0.00	4.93	inf	tr|M2RHY3|M2RHY3_CERS8	Glycosyltransferase family 1 protein
c601034_g1_i3	0.00	5.05	inf	gi|751699172|gb|KIM49104.1|	Glycosyltransferase family 15 protein
c601818_g1_i1	0.00	6.08	inf	tr|F8NSE4|F8NSE4_SERL9	Glycosyltransferase family 2 protein
c601727_g2_i2	0.00	4.46	inf	gi|646307867|gb|KDQ29011.1|	Glycosyltransferase family 2 protein
c602102_g1_i1	0.00	1.47	inf	gi|749763786|gb|KII88520.1|	Glycosyltransferase family 2 protein
c602018_g4_i1	0.00	21.01	inf	gi|761950386|gb|KIY70060.1|	Glycosyltransferase family 20 protein
c593342_g2_i1	0.00	33.59	inf	gi|754372083|gb|KIP04804.1|	Glycosyltransferase family 4 protein
c586703_g1_i3	0.00	7.27	inf	gi|751716241|gb|KIM65247.1|	Glycosyltransferase family 48 protein
c593828_g1_i1	0.00	4.05	inf	tr|G4T6Z6|G4T6Z6_PIRID	Pectate lyase 1
c576776_g1_i2	0.05	2.53	50.56	tr|R7SST1|R7SST1_DICSQ	Galactose oxidase (AA5)
c594647_g2_i1	0.20	25.38	127.24	gi|752370879|gb|KIO28638.1|	Carbohydrate esterase family 12 protein
c592601_g1_i1	0.03	13.15	414.76	gi|749760957|gb|KII85859.1|	Carbohydrate esterase family 4 protein
c600395_g1_i1	0.00	3.82	inf	gi|691792079|emb|CDO72533.1|	Carbohydrate-binding module family 1/GH18
c600797_g1_i1	0.00	21.07	inf	tr|V2WEI0|V2WEI0_MONRO	Carbohydrate-binding module family 12 protein
c600797_g1_i4	0.00	6.68	inf	gi|695558353|ref.|XP_009547634.1|	Carbohydrate-binding module family 12 protein
c590812_g2_i2	0.00	7.48	inf	gi|751000067|gb|KIK42253.1|	Carbohydrate-binding module family 50 protein
EM pathway					
c598496_g1_i1	0.00	15.04	inf	tr|E2M098|E2M098_MONPE	Glucose-6-phosphate isomerase
c596025_g1_i2	0.00	7.96	inf	tr|S7Q9Q0|S7Q9Q0_GLOTA	Glucokinase regulator
c594601_g1_i2	0.00	13.43	inf	gi|751180168|gb|KIL66119.1|	Hexokinase
c585806_g1_i1	0.02	18.89	1107.17	gi|646397316|gb|KDQ61522.1|	Phosphoglucomutase
c602057_g2_i2	0.00	11.14	High	gi|646396300|gb|KDQ60552.1|	Phosphoglycerate kinase
c602057_g2_i3	0.07	11.10	151.91	tr|V2XST0|V2XST0_MONRO	Phosphoglycerate kinase
c598088_g2_i2	0.00	5.55	inf	gi|749895786|gb|KIJ63485.1|	Phosphoglycerate mutase-like protein
c600469_g2_i1	0.00	10.71	inf	gi|749764415|gb|KII89090.1|	Phosphoglycerate mutase-like protein
c572773_g2_i1	0.00	51.66	inf	gi|695531349|ref.|XP_009540596.1|	Enolase
c596540_g1_i2	0.00	4.59	inf	gi|646309757|gb|KDQ30900.1|	Pyruvate kinase
c600228_g1_i1	0.00	15.88	inf	A0A0C9X171|A0A0C9X171_9AGAR	Fructose-1,6-bisphosphatase
c600228_g2_i1	0.00	3.03	inf	gi|807746795|ref|XP_012178192.1|	Fructose-1,6-bisphosphatase
Trehalose and glycogen metabolism					
c593922_g1_i3	0.01	2.87	238.65	tr|A8NBB7|A8NBB7_COPC7	Trehalose-6-phosphate phosphatase
c593922_g1_i2	0.01	1.75	233.45	tr|A8NBB7|A8NBB7_COPC7	Trehalose-6-phosphate phosphatase
c590104_g1_i2	0.00	5.27	inf	gi|576992104|gb|EUC64784.1|	Alpha,alpha-trehalose-phosphate synthase
c601514_g2_i2	0.00	8.92	inf	gi|636619097|ref|XP_008040683.1|	Glycogen phosphorylase
c601447_g1_i3	0.00	24.27	High	tr|S7PZV3|S7PZV3_GLOTA	Glycogen synthase
c601447_g1_i4	0.02	5.34	267.52	tr|R7S2M9|R7S2M9_PUNST	Glycogen synthase
Pentose phosphate pathway					
c599283_g2_i1	0.06	23.65	403.23	tr|S7QHH6|S7QHH6_GLOTA	Glucose-6-phosphate 1-dehydrogenase
c593556_g1_i1	0.00	4.43	inf	tr|F8PCX3|F8PCX3_SERL9	6-Phosphogluconolactonase
c579299_g1_i2	0.00	4.57	inf	gi|695543481|ref|XP_009543815.1|	6-Phosphogluconate dehydrogenase
c596260_g2_i1	0.00	6.07	inf	tr|S7QN86|S7QN86_GLOTA	6-Phosphogluconate dehydrogenase, decarboxylating
c599261_g6_i1	0.40	29.50	73.50	laccaria|B0DN26|B0DN26_LACBS	Transketolase
c586684_g1_i2	0.00	9.94	inf	gi|807755903|ref|XP_012182746.1|	Transaldolase
Ethanol					
c595474_g1_i1	0.00	16.64	inf	gi|754377558|gb|KIP10174.1|	Pyruvate decarboxylase
TCA cycle					
c578376_g1_i1	0.00	20.05	inf	tr|A8N9T7|A8N9T7_COPC7	Pyruvate carboxylase
c590892_g1_i1	0.00	16.91	inf	gi|924125151|emb|CUA68456.1|	Pyruvate dehydrogenase E1 component subunit alpha
c602021_g6_i1	0.00	20.10	inf	tr|D8PKQ0|D8PKQ0_SCHCM	Acetyltransferase component of pyruvate dehydrogenase
c569980_g1_i1	0.00	12.30	High	tr|S8EDA9|S8EDA9_FOMPI	ATP-utilising phosphoenolpyruvate carboxykinase
c601865_g3_i1	0.00	21.12	inf	tr|R7STC2|R7STC2_DICSQ	ATP-utilising phosphoenolpyruvate carboxykinase
c601865_g3_i2	0.00	9.71	inf	tr|R7STC2|R7STC2_DICSQ	ATP-utilising phosphoenolpyruvate carboxykinase
c569980_g1_i2	0.09	16.00	168.90	gi|749767591|gb|KII92023.1|	ATP-utilising phosphoenolpyruvate carboxykinase
c596905_g1_i4	0.00	12.95	inf	tr|F8ND69|F8ND69_SERL9	Malate dehydrogenase
c596978_g1_i2	0.01	18.99	1489.83	gi|599119246|ref.|XP_007387111.1|	NAD-malate dehydrogenase
c596978_g1_i1	0.01	13.56	1360.27	gi|599119246|ref.|XP_007387111.1|	NAD-malate dehydrogenase
c601738_g1_i3	0.00	9.89	inf	gi|751717279|gb|KIM66280.1|	Succinyl-coa synthetase alpha chain, gdp-forming
c600881_g1_i4	0.01	1.61	299.16	tr|M2RB43|M2RB43_CERS8	Succinyl-CoA synthetase beta chain SSC-beta
c600756_g1_i1	0.00	11.35	inf	gi|646392723|gb|KDQ57237.1|	Homocitrate synthase
c601939_g2_i2	0.05	13.42	261.20	gi|754372092|gb|KIP04812.1|	Isocitrate dehydrogenase
c583773_g1_i1	0.00	5.65	inf	gi|636616019|ref|XP_008039144.1|	Peroxysomal citrate synthase
c585571_g1_i2	0.03	2.64	81.61	gi|751696020|gb|KIM45999.1|	Aconitase
c591611_g1_i1	0.00	10.96	inf	gi|636618909|ref|XP_008040589.1|	Aconitate hydratase
c575147_g1_i1	0.00	5.73	inf	gi|597981973|ref|XP_007363646.1|	2-Oxoglutarate dehydrogenase mitochondrial precursor
c597062_g1_i2	0.00	16.03	inf	gi|749834860|gb|KIJ12495.1|	Succinate dehydrogenase
c602048_g2_i2	0.00	5.52	inf	gi|749830076|gb|KIJ08477.1|	Succinate dehydrogenase
c600915_g2_i1	0.07	13.16	190.79	tr|M2PCH1|M2PCH1_CERS8	Succinate dehydrogenase
c600002_g1_i4	0.00	4.55	inf	tr|D8Q9I2|D8Q9I2_SCHCM	Fumarate reductase
c600002_g1_i1	0.00	17.05	inf	gi|752345450|gb|KIO04521.1|	Fumarate reductase
Leading to AAs					
c601513_g2_i5	0.00	6.72	inf	tr|M2PP80|M2PP80_CERS8	Glutamine sythetase
c600930_g1_i1	0.00	9.88	inf	tr|V2X687|V2X687_MONRO	Glutamate synthase

*Basidomycota* carbohydrate active enzymes were identified as upregulated in response to contamination conditions, with the majority only expressed in contaminated trees (only 9 contigs were also expressed in control trees). Of these, 20 contigs belonged to one of nine glycosyl hydrolase (GH) families, eight contigs to one of six glycosyl transferase (GT) families and one contig was a pectin/pectate lyase (PL; pectate lyase 1). The most highly represented group was GH13, with seven contigs (5 distinct enzymes including 3 likely isoforms) including a secreted alpha-amylase. It is possible that these may represent extracellular invertase activity (sucrose hydrolysis) as opposed to native fungal α-glucan metabolism.

The most abundant CAZy contig (104.28 tpm) was a GH45 Barwin-like endoglucanase (expansin family protein) (Table 1). Expansin domain containing proteins have previously been recognised in *Laccaria bicolor* as expressed only in ECM tissue [106], which is fascinating in light of the functional similarity to cell wall loosening in plants [107] and if considered in the context of non-necrosis inducing *Salix* root tissue remodelling and potential interaction with middle lamella and primary cell walls. In agreement with this, a carbohydrate esterase family 12 protein (putative rhamnogalacturonan acetylesterase, pectin-related) [108] annotated only from the orchid symbiote *Tulasnella calospora* was DE. Further to this, a pectin/pectate lyase was also DE, recently identified as one of the few cell wall-degrading enzymes retained by the ECM fungus *Tuber melanosporum* for cell wall remodelling of *Corylus avellana* as part of their symbiotic interaction [109]. The second most abundant CAZy was GH131 (best annotated from *Hebeloma* but with equally good hits in *Plicaturopsis*, *Laccaria*, *Jaapia*, *Tulasnella* and *Gelatoporia*), including a cellulose binding module (CBM1) with broad β-glucanase activity. While this activity allows for the potential for saprophytic action, remodelling of the plant cell wall and progression through lamella is in agreement with ECM colonisation of plant roots towards the effective formation of the hartig net [110] and, interestingly, GH131 has previously been identified as expressed in *Jaapia argillacea* as a potential adaption from saprotrophic ancestors [92]. Three GH5 contigs (exo-beta-1,3-glucanase) were also highly expressed including an ectomycorrhiza-upregulated exo-beta-1,3-glucanase [106, 111]. In disagreement with this is the lack of identifiable lignases or maganase-dependent peroxidases necessary for deconstruction of heavily lignified middle lamella as well as the differential expression of galactose oxidase (AA5), a secreted extracellular catalyse which has twice as much activity on galactomannan (mannan backbone with galactose sidechains) compared to galactose [112], important given this very common hemicellulose component is more indicative of plant secondary cell walls [113].

In terms of the potential direct interface between the plant and putative ECM *Basidiomycota*, two *Stereum hirsutum* ricin B-like lectins of the carbohydrate-binding module family 13 (CMB13) contigs were very highly abundant in treated conditions at 144.48 tpm (14th most abundant, 1350 FC) and 98.05 tpm (22nd most abundant, 350 FC). Interchangeable homologous hits were also found using secondary annotation within *Heterobasidion*, *Fomitiporia* and *Jaapia*. Lectin carbohydrate-binding is well documented as one of the means of intense binding at the interface between the ECM hartig net and the plant host cell wall [114, 115]. CMB13 has been shown to have specificity for backbone xylan (such as the majority of hemicellulose within willow root 2° root cell wall), although is also found in a diverse array of non-xylanase glycosyl hydrolases [116].

The metabolic machinery necessary as a downstream consequence of monosaccharide import (in light of very highly expressed MST) includes the Embden-Meyerhof (EM) pathway, pentose phosphate pathway and tricarboxylic acid cycle (Additional file 7). Trehalose and glycogen metabolism within the EM pathway, clearly represented within DE contigs, are glycolytic pathways expected in ECM fungi [117]. Alpha,alpha-trehalose-phosphate synthase (TPS1), two trehalose-6-phosphate phosphatases (TPS2), two glycogen synthases, glycogen phosphorylase, glucokinase regulator, hexokinase, phosphoglucomutase, glucose-6-phosphate isomerase, two phosphoglycerate kinases, two phosphoglycerate mutase-like proteins, enolase, pyruvate kinase and two fructose-1,6-bisphosphatases were identified as DE and in higher abundance in root from contaminated soil. Within the pentose phosphate pathway: glucose-6-phosphate 1-dehydrogenase, 6-phosphogluconolactonase, two 6-phosphogluconate dehydrogenases, transketolase and transaldolase were also DE. While carbon was fated towards ethanol fermentation (pyruvate carboxylase was DE), the citric acid cycle was also comprehensively represented as upregulated: pyruvate dehydrogenase (E1 component subunit alpha; acetyltransferase component), four phosphoenolpyruvate carboxykinases, pyruvate carboxylase, three succinate dehydrogenases, two fumarate reductases, succinyl-coa synthetase (alpha chain and beta chain), three malate dehydrogenases, 2-oxoglutarate dehydrogenase, two homocitrate synthases, two aconitate hydratases and isocitrate dehydrogenase, supporting carbon scaffold assembly towards amino acid production.

*Nitrogen management*

This extensive representation of the citric acid cycle upregulated in *Basidiomycota* from contaminated conditions is relevant in the context of potential amino acid export to *Salix* roots, given very similar ECM expression profiles in ECM *Laccaria* and *Tuber* [118, 119], and considering the differential expression of contigs encoding glutamate synthase and glutamine synthetase (Table 1). Contigs encoding N-related transport machinery were highly expressed (Table 2), including two ammonium transporters, which were some of the most highly abundant *Basidiomycota* contigs present in the samples (AmtB, tpm 66.39; Amt1, tpm 64.18) as well as a very highly expressed contig encoding a putative FUN34-transmembrane protein involved in ammonia production (tpm 47.03). Additionally, a peptide/nitrate transporter, five amino acid transporters, a purine transporter, a glutathione transporter, two oligopeptide transporters (OPTs), two plasma membrane h+ symports, two mitochondrial carriers as well as urease, amine oxidase, D-aspartate oxidase and two carbon-nitrogen hydrolases were upregulated, all enzymes involved in the reduction of organic nitrogen compounds and ammonia production (Additional file 7). This wide ranging selection of N-related compound transporters identified as DE very closely matched those previously reported in the ECM fungi *Paxillus involutus* [120] and *Laccaria bicolor* [121].

**Table 2:** Fungal nitrogen-related DE genes from upregulated *Basidiomycota*. EBSeq [42, 43] was used to estimate posterior probability of differential expression (PPDE) ≥ 0.95. A full DE transcript list including expression data, functional description (if available), gene ontology terms (if available) and secondary annotation (if available) is provided in Additional file 6

Query id	Cont mean tpm	Treat mean tpm	FC	1° annotation id	Subject description
c596378_g2_i3	0.14	66.39	477.06	gi|749759516|gb|KII84515.1|	Ammonium transporter
c596378_g2_i2	0.14	64.18	458.91	tr|Q8NKD5|Q8NKD5_HEBCY	Ammonium transporter
c599446_g2_i1	0.14	57.28	405.50	gi|660970957|gb|KEP54657.1|	Rab GTPase family protein
c555426_g1_i1	0.04	47.83	1162.00	gi|646294952|gb|KDQ16118.1|	Small GTPase-binding protein
c590384_g1_i3	0.36	47.03	130.17	tr|R7RZR8|R7RZR8_PUNST	fun34-transmembrane protein of ammonia production
c597663_g1_i3	0.02	31.86	1277.49	tr|R7S8J0|R7S8J0_TRAVS	Small GTPase-binding protein
c576075_g1_i1	0.02	22.26	1356.92	gi|691790442|emb|CDO74199.1|	Amino acid transporter
c597663_g1_i1	0.01	20.67	2581.27	tr|V2X8E2|V2X8E2_MONRO	Small GTPase-binding protein
c596122_g2_i1	0.00	18.08	4389.38	tr|V2WTU3|V2WTU3_MONRO	Plasma membrane h(+)-atpase 1
c592122_g2_i1	0.00	18.00	inf	tr|S7PWC9|S7PWC9_GLOTA	Mitochondrial carrier
c598258_g3_i1	0.00	16.68	inf	gi|597977025|ref.|XP_007362384.1|	Urease
c597755_g1_i1	0.00	16.37	inf	gi|754376368|gb|KIP08998.1|	Subtilisin-like serine protease pepC
c601027_g2_i1	0.00	15.18	inf	gi|660966237|gb|KEP50782.1|	Serine carboxypeptidase
c590409_g1_i1	0.00	13.93	inf	tr|E2LF88|E2LF88_MONPE	Oligopeptide transporter
c602018_g3_i1	0.01	12.29	949.61	tr|R7S2N4|R7S2N4_PUNST	Endopeptidase
c601958_g7_i1	0.00	11.68	inf	tr|M2R776|M2R776_CERS8	Aminopeptidase 2
c597846_g2_i1	0.01	11.17	1682.64	gi|695572973|ref|XP_009551472.1|	Purine transporter
c598346_g1_i1	0.00	10.15	inf	gi|807753175|ref.|XP_012181382.1|	ATP-dependent metallopeptidase Hfl
c596122_g2_i3	0.05	9.94	216.32	laccaria|A0A0C9XV60_9AGAR	Plasma membrane ATPase
c600930_g1_i1	0.00	9.88	inf	tr|V2X687|V2X687_MONRO	Glutamate synthase
c575280_g1_i3	0.00	9.72	inf	tr|F8NRP9|F8NRP9_SERL9	Oligopeptide transporter
c586208_g2_i4	0.00	9.42	inf	gi|595766652|ref|XP_007261869.1|	Small GTPase
c599446_g3_i1	0.00	8.68	inf	tr|B0CRR7|B0CRR7_LACBS	Ras-related protein Rab-5B
c597351_g5_i3	0.05	8.59	185.55	tr|D8PW94|D8PW94_SCHCM	Carbon-nitrogen hydrolase
c600850_g1_i2	0.00	8.38	inf	gi|751184086|gb|KIL70023.1|	Aspartic peptidase A1
c591514_g1_i1	0.00	8.25	inf	gi|597967903|ref.|XP_007360041.1|	Ras protein
c600898_g1_i3	0.02	7.43	316.59	tr|M2RCY8|M2RCY8_CERS8	Zinc carboxypeptidase
c590849_g1_i2	0.00	7.23	inf	gi|751185175|gb|KIL71111.1|	Rab-type small GTP-binding protein
c594481_g1_i1	0.00	7.10	inf	uniparc|UPI000444A56C	Amino acid transporter
c595033_g1_i1	0.00	7.06	inf	tr|V2WPM3|V2WPM3_MONRO	Ras GTPase-activating protein
c596094_g1_i2	0.00	6.76	inf	gi|695535578|ref|XP_009541732.1|	Metallo peptidase M16B
c601513_g2_i5	0.00	6.72	inf	tr|M2PP80|M2PP80_CERS8	Glutamine synthetase
c587799_g1_i1	0.00	5.74	inf	laccaria|B0CVJ7|B0CVJ7_LACBS	Aspartic peptidase A1
c583462_g2_i3	0.00	5.67	inf	tr|A8N171|A8N171_COPC7	Glutathione transporter
c570564_g1_i1	0.00	5.19	inf	gi|827762056|gb|KLO16666.1|	Zn-dependent exopeptidase
c594381_g2_i2	0.00	5.13	inf	gi|599097713|ref.|XP_007380030.1|	Zn-dependent exopeptidase
c600158_g1_i5	0.03	5.12	203.31	tr|F8NFV4|F8NFV4_SERL9	rab GDP-dissociation inhibitor
c583177_g1_i2	0.00	4.84	inf	gi|751693001|gb|KIM42985.1|	Transmembrane GTPase fzo1
c600177_g1_i1	0.00	4.71	inf	gi|914260206|gb|KNZ75358.1|	Peptide/nitrate transporter
c601958_g3_i1	0.00	4.68	inf	tr|M2R776|M2R776_CERS8	Aminopeptidase 2
c594481_g1_i2	0.00	4.50	inf	gi|618814194|ref.|XP_007309736.1|	Amino acid transporter
c567674_g1_i1	0.00	4.48	inf	gi|599098740|ref.|XP_007380368.1|	Carbon-nitrogen hydrolase
c597783_g1_i3	0.00	4.45	inf	tr|F8QHR7|F8QHR7_SERL3	Glutamate carboxypeptidase
c594170_g1_i2	0.00	3.78	inf	gi|751737188|gb|KIM85475.1|	Mitochondrial carrier
c601872_g2_i1	0.00	3.76	inf	gi|628824529|ref|XP_007762568.1|	ATP-dependent metallopeptidase Hfl
c599577_g1_i2	0.00	3.73	inf	gi|597902114|ref.|XP_007298356.1|	Peptidase M24A methionine aminopeptidase
c602154_g2_i2	0.01	3.51	328.31	gi|761927343|gb|KIY48481.1|	D-aspartate oxidase
c600522_g1_i1	0.00	3.41	inf	gi|695555686|ref.|XP_009546958.1|	Bleomycin hydrolases/aminopeptidases (cys family)
c598679_g1_i2	0.00	3.40	852.70	tr|M2QPL6|M2QPL6_CERS8	Aspartic-type endopeptidase
c601070_g1_i3	0.00	3.26	inf	gi|599098141|ref|XP_007380172.1|	Amine oxidase
c595365_g1_i2	0.00	2.95	inf	tr|E2LXQ1|E2LXQ1_MONPE	Amino acid transporter
c601230_g1_i3	0.01	2.92	561.26	gi|695571495|ref|XP_009551148.1|	Metallo peptidase M24B
c600602_g1_i1	0.00	2.84	inf	gi|695544112|ref.|XP_009543978.1|	Small monomeric GTPase
c601825_g4_i5	0.00	2.32	inf	gi|754370636|gb|KIP03412.1|	Vacuolar amino acid transporter

A broad suite of proteases was also DE including four endopeptidases (including subtilisin and bleomycin), two Zn-dependent exopeptidases, three carboxypeptidases (glutamate, serine and zinc), four metallopeptidases, a methionine aminopeptidase, two putative aminopeptidase isoforms and two distinct aspartic peptidase A1 (Table 2). Secreted suites of proteases are thought to be similar between *Basidiomycota* saprotrophs and EMC [90]. However, the extracellular protein degradation pathways identified here distinctly match those expressed in the EMC *P*. *involutus* [120]. One of the markers identified from *Laccaria biocolor* but recognised across ECM are Ras/Ras-like proteins [85, 122, 123], small GTPases involved in cargo sorting in coated vesicules [124] (vesicular turnover is thought to be high in ECM due nutrient and signal exchange with hosts [125]) that have been shown to increase in transcript abundance after successful establishment of symbiosis [126]. Eleven Ras-like proteins were DE and in high abundance in the phytoremediation responsive *Basidiomycota*, importantly including likely Ras, or Ras interacting, proteins known as host interaction-specific ECM markers identified in *Laccaria* [85]. These comprised three highly abundant uncharacterised small GTPase-binding proteins (47.83 tpm, 31.86 tpm, 9.42 tpm), Rho1 (20.67 tpm), Ras-related protein Rab-5B, Ras protein, Rab-type small GTPase, Ras GTPase-activating protein, Rab GDP-dissociation inhibitor, GTPase foz1, Sar1-like protein member of Ras-family as well as an extremely highly expressed Rab5 (ypt5) GTPase family protein (57.28 tpm), involved in endocytotic vascular trafficking [127].

While a clear depth of functional detail can be revealed by metatranscriptomics, the majority of putative fungal proteins were uncharacterised (having no functionally characterised homologue), a useful reminder that the vast majority of the natural world remains to be explored.

#### Bacteria

##### Polyadenylation in bacteria

Polyadenylation of RNA has long been known to occur in bacteria [128, 129]. Some studies have established the potential involvement of polyadenylation in mRNA degradation [130, 131] and RNA quality control [132, 133] in a limited number of bacterial species, although the contemporary picture of polyadenylation in bacteria suggests complexity beyond this, as recently described by Kushner [134]. Using pulse-labelling, levels of polyadenylated RNA have been measured at up to 15% of RNA in *Escherichia coli* [135] (in contrast to those of up to 25% in gram-positive *Bacillus brevis* [136]) and more recent research using microarray analysis revealed, in an *E*. *coli* K-12 wild-type transcriptome, that 90% of transcribed ORFs underwent some degree of polyadenlyation [137].

Within the plant transcriptomic research community, bacterial mRNA is routinely discarded during early quality control of common bioinformatics pipelines as distinct from the target organism of study or discarded intrinsically when mapping to a reference genome. Gonzalez et al. [46] recently reported how the sub-selection of only transcriptomic sequences expected a priori can confound biological results, the example leading to *S*. *purpurea* biotic stress genes being misidentified as abiotic stress responsive genes due to a strong treatment-specific interaction of a plant herbivore (*Tetranycus urticae*). Similarly, Brereton et al. [2] demonstrated the potential for mistaken mapping (mis-mapping) RNA-seq reads from unexpected foreign organisms to technically confound results by mapping known foreign sequences to the *S*. *purpurea* genome. Given these potential pitfalls, it would seem prudent to acknowledge the complexity of extra-laboratory biological systems involving higher eukaryotes by investigating all mRNA molecules present within any sample even when strong expectations exist within the experimental design. The approach is limited here as an unknown absolute proportion (and community) of bacterial mRNA could be lost during polyA enrichment.

**Fig. 6:**
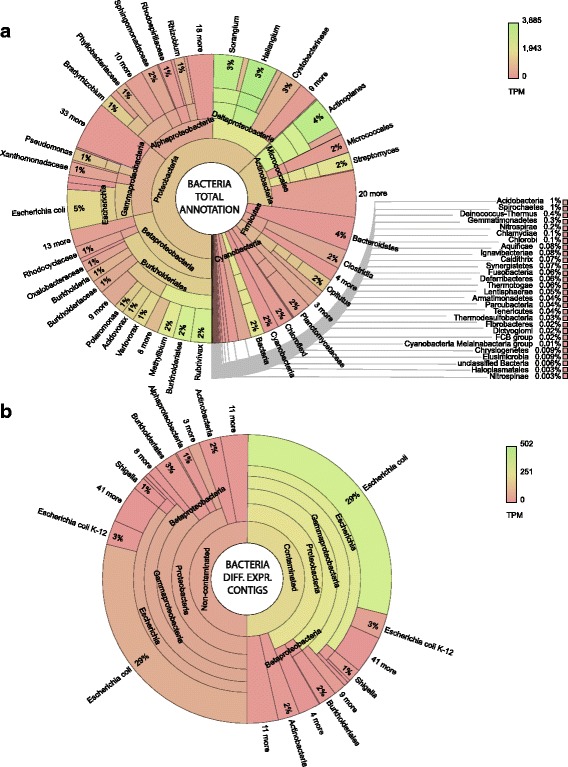
Bacterial contigs, total and differentially expressed (DE) transcript origin. Krona graphs [47] represent **a** total annotation of bacterial transcripts (including non-DE) and **b** annotation of DE bacterial transcripts. The proportion of each taxonomic grouping is defined by the number of unique transcripts, whereas the colour represents the relative abundance (transcripts per million tpm) of transcripts in each taxon. A full contig list including expression data, functional description (if available), gene ontology terms (if available) and secondary annotation (if available) is provided in Additional file 4. A list of bacterial DE transcripts (including protein coding sequences within polycistronic contigs annotated with transdecoder is provided in Additional file 10. Interactive versions of these Krona graphs available at: https://github.com/gonzalezem/Tripartite_Metatranscriptomics_article

Despite polyA enrichment, an extremely broad diversity of bacteria was observed within the system (including contigs that were not DE; Fig. 6a)). The majority of these contigs derived from *Proteobacteria*, including *Alphaproteobacteria* (14%), *Betaproteobacteria* (34%), *Deltaproteobacteria* (13%) and *Gammaproteobacteria* (23%). *Actinobacteria* (14%), *Bacteroidetes* (4%) and *Firmicutes* (4%) were also highly represented. This community makeup is similar to that previously reported in metagenomic studies of contaminated soils [25].

A different picture was discerned in DE contigs (Fig. 6b); of 638 DE contigs, 86% were in higher abundance in contaminated trees. *Enterobacteriaceae* species were the most represented, annotating 72% of all the bacteria contigs. Importantly, 100% of these *Enterobacteriaceae* contigs were of higher abundance in contaminated trees (Fig. 2b, c), suggesting strong biological association with contamination and/or the other organisms. Therefore, their functionality was explored, albeit with caution. Of potential relevance to this, growth under more challenging conditions than standard rich media, or repression of growth using transformation, can increase the level of polyadenylated transcripts within *E*. *coli* [138–140].

##### Decoding polycistronic transcriptional units

Determining transcriptional units (putative operons) in bacteria is computationally problematic in terms of prediction from genomic sequence [141, 142]. While prediction of *E*. *coli* K12 operon structure is as advanced as in any bacteria, little confidence can be established in the transferability of such predictions given operon variation across organisms and very high responsiveness to environmental change. The unique nature of any extra-laboratory environment, such as that explored here, as well as the high likelihood of numerous novel organisms present (not the least being the bacteria themselves present within the system) further complicates transcriptional unit prediction. That being said, top-down de novo assembly provides very high confidence in the presence of observed sequences within the system, of particular technical value is the high requirement for equal mapping coverage applied within Trinity’s contig assembly [3]. Of the 639 DE and potentially polycistronic contigs annotated as bacterial in origin, 3134 protein coding regions were identified using Trinity’s transdecoder. In total, 489 of these contigs were identified as deriving from species within the family *Enterobacteriaceae* (100% in higher abundance in contaminated trees) comprised 2834 proteins, although a substantial proportion were duplicates with only minor sequence variation (Additional file 10). These could represent common expression by distinct organisms or multiple operons from a single organism (being somewhat reminiscent of eukaryotic splice variants at a sequence level). The majority of the 2834 *Enterobacteriaceae* putative proteins were annotated from *E*. *coli* (68%, across a broad spectrum of strains, Additional file 10) or the genus *Shigella* (29%). A recent identification and genome sequence of a poplar growth promoting endophyte (*Enterobacter* sp. 638) by Taghavi et al. [31] shared similar levels of homology as those found here but at a genome level, with 69% of predicted CDS being similar to *E*. *coli*.

##### Upregulated bacterial gene function

A broad spectrum of contigs encoding proteins putatively involved in survival within a rhizospheric environment were identified as DE due to contamination conditions (Additional file 10), including those putatively interacting extracellularly and/or intracellularly with ECM hyphae and/or plant roots. As the vast majority of DE bacterial genes were best annotated as *Enterobacteriaceae* (either *E*. *coli* or *Shilgella* strains), *E*. *coli* K-12 MG1655 gene nomenclature was used where possible.

##### Hydrocarbon degradation and biosurfactant production

**Fig. 7:**
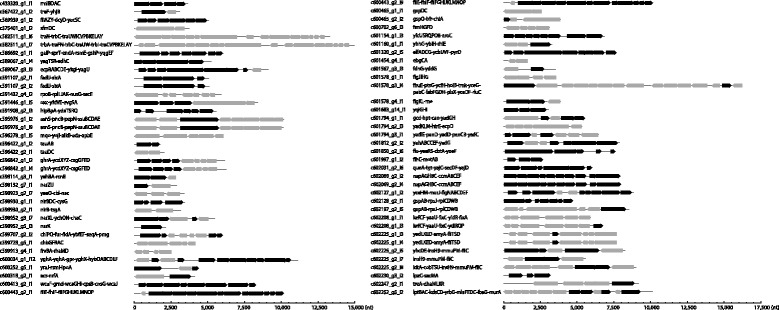
A selection of differentially expressed bacterial putative operons in higher abundance in roots of contaminated trees (and discussed in the text). Bacterial contigs were first identified within the assembly as best annotated with a single bacterial protein. To find multiple potential coding regions within bacterial polycistronic sequences, we used TransDecoder software (https://transdecoder.github.io/) [3] with default parameters. A final hand annotation step was included to remove a minor number of overlapping uncharacterised ORFs. Precedence of transcriptional unit structure (putative operons) was verified in all cases against the database of prokaryotic operons (DOOR [48]) unless otherwise stated. The in-house contig label is presented with the structure of the putative operon annotated using *E*. *coli* nomenclature. The three putative operons c60225_g2_i5, c60225_g2_i7 and c60225_g2_i8 all include the transposable element insH9, similar read coverage may falsely conjoin up- and downstream DE sequence combinations around the common insert. A full list of bacterial DE putative operons (transcriptional units) including expression data, functional description (if available), gene ontology terms (if available) and secondary annotation (if available) is provided in Additional file 10

For PAH degradation, bacterial dioxygenase enzymes are considered the principal means of ring fission [26, 143] (although cytochrome p450 monooxygenase are common), whereas alpha-ketoglutarate-dependent dioxygenases (*alkB*) are one of the most recognised enzymes driving degradation of aliphatic hydrocarbons [25] (not to be confused with alkane monooxygenase, *alkB*). The contig c596278_g1_i5 was upregulated here (containing *yojIalkBadaapbEmqo*), functionally comprising an ABC transporter ATP-binding protein (*yojI*), alpha-ketoglutarate-dependent dioxygenase (*alkB*), regulatory protein (*ada*), thiamine biosynthesis lipoprotein (*apbE*) and malate:quinone oxidoreductase (*mqo*) (Fig. 7 and Additional file 10). Additionally to this, a number of genes well characterised as accompanying toluene tolerance [144, 145] were DE, including a putative operon which contained the toluene transporter subunits *mlaCDEF* and *kdsCDlptACkdsCD* encoding a lipopolysaccharide export system. Degradation of alkanesulphonates, previously identified as involved in crude oil degradation by metagenomic study [25], occurs through desulfonation and relies on expression of the operon *ssuABCDE* [146, 147] comprising an alkanesulfonate transporter subunit, a putative alkanesulfonate transporter subunit, an FMNH(2)-dependent alkanesulfonate monooxygenase, putative aliphatic sulfonate binding protein and NAD(P)H-dependent FMN reductase. Here, the entirety of the *ssuABCDE* operon was assembled and DE in higher abundance in contaminated conditions (two similar contigs c595976_g1_i2 and c595976_g1_i9). As would be expected alongside the *ssu* system, the associated *tau* system (comprising *tauABCD* genes) was also upregulated within two separate putative operons containing *tauAB* proteins (taurine transporter subunits A and B; c596422_g1_i2) and *tauCD* (taurine transporter subunit C and alpha-ketoglutarate-dependent taurine dioxygenase; c596422_g2_i1). These oxidising mechanisms, such as the well-known alkanesulfonate monooxygenase *ssuD* [148], suggest the bacterial species present in association with willow roots is actively expressing an enzyme suite capable of degrading the hydrocarbons present within contaminated soil and so may play an important role within any tripartite interaction of mutual benefit.

Genes necessary for three biosurfactants were found to be most represented in metagenomic study of crude oil contaminated soil [25], (in descending order of abundance): trehalose lipids [149], polyol lipids and mono/di-rhamnolipids [150]. Interestingly, the principal enzymes involved in the trehalose degradation pathway were DE, with two putative operons, the first containing *treAdhaLKMR* (c602247_g2_i1), from *E*. *coli* and the second (c567422_g1_i2) containing *treF* (from *shigella*) alongside a transcriptional regulator *yhjB* (from *E*. *coli*)*.* These potential operons comprise the periplasmic trehalase (*treA*) and PTS proteins (*dhaLKM*, although no transport activity is expected [151]) as well as the cytoplasmic trehalase (*treF)* expected for trehalase utilisation [152]. While this suggests trehalose may not be being employed as a biosurfactant, it is compelling in terms of the potential interaction with ECM *Basidiomycota* as the specific contamination-responsive DE contigs include trehalose biosynthesis genes. The entire *rmlABCD* (often termed *rfb*) operon (c433320_g1_i1) was in higher abundance in contaminated conditions, comprising the l-rhamnose synthesis genes essential for rhamnolipid production in *E*. *coli* [150, 153]. Additionally to this, rhamnulose-1-phosphate aldolase (*rhaD*), rhamnulokinase l (*rhaB*) and rhamnose-isomerase (*rhaA*) were all upregulated allowing provision of l-rhamnose from dihydroxyacetone-phosphate [154], present within two operons comprising *rhaDyiiL* with genes for PTS system IIA and IIB components (c599913_g4_i1, operon structure common in *Firmicutes*) and *rhaABSRyifCLMaidB* (c433320_g1_i1). While the l-rhamnose synthesis pathway is considered well characterised, the exact 3-hydroxy fatty acid precursors of rhamnolipids are less confidently known, although it has been hypothesised that both the type II fatty acid synthesis pathway [155] and β-oxidation [156] can provide lipid precursors. An operon was upregulated (c601578_g3_i4) containing numerous members of the type II fatty acid synthesis pathway (FAS-II): *fabG* (syn: beta-ketoacyl-ACP reductase, similar to *rhlG* of rhamnolipid synthesis in *Pseudomonas aeruginosa* [157]) *fabDFHychHyceDFGthiK*, the operon structure being unsurprising in *E*. *coli*. *rhlAB*, recognisable as driving rhamnolipid production in *Pseudomonas aeruginosa* [155], was not detected however, and an operon containing *fabA*, potentially a competitor for β-hydroxydecanoyl-ACP [158], was DE. Of β-oxidation, two *fadIJsixA* upregulated operons (c591107_g2_i1 and c591107_g2_i2), a common operon if *E*. *coli* K-12, were in higher abundance under contaminated conditions. *fadIJ* are recently discovered homologues of β-oxidation *fadAB* in *E*. *coli* with a suggested preference for short and medium chain fatty acid degradation [159].

##### Community interaction

These DE potential operons encompass a wide array of genes considered cryptic in *E*. *coli* (silent under standard laboratory conditions), for example, cryptic genes in *E*. *coli* K-12 substr. MG1655 [160] that were expressed here included *ecpABCD* (c589067_g3_i3), *ebgAC* (c601454_g4_i1), *gspAB* c602128_g2_i1), *gspCD* (c600465_g1_i1) and *chiAgspO* (c600465_g2_i2) (Fig. 7 and Additional file 10). The expression of genes which are silent in many *E*. *coli* species cultured under laboratory conditions is perhaps unsurprising given the mycorrhizal environment; this is further supported by DE of genes representing fungiphile metabolism [161] in the *Enterobacteriaceae* species responding to contamination conditions. The most convincing in this regard were genes involved in interaction with chitin (fungal cell wall) and the well-known fungal exudate oxalate, used for habitat manipulation through pH lowering and increasing availability of nutrients [162] as well as for lignocellulosic degradation in many *Basidiomycota*. The putative operon c591446_g1_i5 comprised the well-studied two-component (stimulus-response) regulatory system *evgSA* [163] with an Oxalyl-CoA decarboxylase, acetyl-CoA:oxalate CoA-transferase and transporter similar to the metabolic machinery essential for *E*. *coli* tolerance of oxalate [164]. The putative operon c599707_g2_i2 comprised 8 proteins including *chiPQ* (*oprD* family, a chitoporin for uptake of chitosugars and chitosugar-induced lipoprotein which is not constitutively expressed [165, 166]) and a phosphoglymutase, suggesting tight interaction with the chitin phosphotransferase system (PTS) system [167]. In line with this is the upregulation of a potential operon containing four proteins including *chbBC* (N,N′-diacetylchitobiose-specific enzyme IIB component and permease IIC component of the PTS system) alongside *osmE* and *nadE* (an uncharacterised osmotically induced lipoprotein and NH(3)-dependent NAD(+) synthase). Further supporting this, the operon *chbACFGR* (c599728_g5_i1) comprising the more classically recognisable chitobiose operon [168], with the exception that *chbB* is absent (expressed in the previous operon alongside a distinct *chbC*). The cryptic chitinase *chiA* (c600465_g2_i2) was also expressed in an operon including *bfr* and *gspO*, pertinent given the ECM fungal interaction suggested by gene expression (potentially explaining cryptic expression as the fungal environment is difficult to replicate in culture) and logical in light of associated [169] DE of (non-constitutively expressed) type II general secretion systems (*gspAB*, c602128_g2_i1 and *gspCD*, c600465_g1_i1 functionality is discussed below).

While the clear expression of chitin degrading mechanisms could suggest the interaction with ECM fungi is endocellular biotrophy or necrotrophy, the possibility of extracellular biotrophy cannot be discounted as any complex interaction could involve modification of, or macro-(biofilm)-adhesion with, the Hartig net involving chitinases. In line with this, Chittero et al. [170] report biotrophic interaction of *Pseudomonas fluorescens* and *Bacillaceae* through the use of chitinases with fruit bodies of the ECM *Tuber borchii*; while others found bacterial chitinase production did not inhibit ECM fungi [171, 172] and that when comparing rhizospheric and ectomycorrhizosphere bacteria, those capable of hydrolysing chitin were present only within the ectomycorrhizosphere [173].

*Biofilm formation*

There are five stages to biofilm formation which are generally accepted: (i) surface contact and reversible attachment, (ii) irreversible attachment, (iii) microcolony formation and early development of architecture, (iv) maturation and (v) dispersal. The accomplished mini-review by Van Houdt and Michiels [174] outlines the proposed surface determinants associated with each stage in *E*. *coli*. Flagella have been associated with the initial surface contact and reversible attachment as well as final dispersal and motility (although development and maturation stages are not dependent on flagella). Irreversible attachment coincides with production of type I fimbriae, extracellular polysaccharides and curli fimbriae. Development of biofilm architecture also involves curli and extracellular polysaccharides as well as colonic acid production and the self-recognising adhesion antigen 43 (also called *flu*). Maturation of the biofilm architecture also involves colonic acid production and curli as well as conjugative pili. Dispersal of bacterial cells back into the environment again involves flagella. Operons encoding proteins from each of these processes were DE, all being in higher abundance in contaminated conditions. The formation and adhesion of bacterial biofilms to fungal hyphae has been well characterised as facilitating the exchange of nutrients [28, 29] but has also been shown to allow the mobilisation of pollutant degrading bacteria through the environment for mutual benefit [175, 176].

As substantial numbers of genes involved in biofilm formation were DE, we compared gene expression here to the list generated by Tenorio et al. [177], who systematically investigated genes affecting biofilm formation in *E*. *coli* through overexpression. While the study was performed using LB media, we expect extensive overlap in machinery involved in potential biofilm formation within an ECM environment. Ten genes were identified as fundamentally altering biofilm abundance: *ccmf*; *fdrA* (syn *yahF*); *flgFGIL*, *fliH* (c601578_g1_i1); *gspA*; *secY* and *wcaK* (Fig. 7 and Additional file 10). All were DE here with the exception of *secY* and *wcaK*; however, other colonic acid production genes were upregulated (*wbz wcaJLF*) as well as extensive DE of the *secYEG* translocon (peptide export complex proteins) including, *secA*, *secE* and *SecDFyajC*. A total of 11 of the 35 genes whose overexpression altered biofilm architecture in LB media were DE with increased abundance in roots of contaminated soil, while 11 of the 27 genes causing filmentous morphology of the biofilm were also upregulated. The flagellin synthesis genes *FliA* and *FliC*, and flagellar biosynthesis master regulator *FlhC*, were identified as in high abundance in treated samples as well as *fliEFGHIJKLMNOP*, *fliDST*, *flgKL* and *flgGHIL*. Flagella can allow increased fitness when growing/forming biofilm on fungal hyphae [178], although *E*. *coli* are thought to require flagella only for the initial stages of biofilm formation [177]. Expression of these genes has been shown to be positively regulated by polyadenylation [139]; this is particularly interesting given the suggestion that this mechanism could be specifically in place to allow the bacteria to adapt and survive in challenging environmental conditions (through PAPI regulation).

The chaperone-usher pathway is a delivery system for type I pili through the outer membrane. The periplasmic transport of proteins is essential for successful pili biogenesis and the chaperone-usher pathway utilises the SecYEG translocon to achieve this, also upregulated here in contaminated conditions. Concordantly, the *fimDFGH* operon (c600702_g6_i3, structurally common in *E*. *coli* K-12 strains), encoding the outer membrane usher protein for type 1 fimbrial synthesis and three minor component of F-type fimbriae, was upregulated in contaminated conditions, as was *sfmCD* (c575401_g1_i2; *fimACI* was not identified as DE). Interestingly, given the likely interaction with fungal cells, *sfm* is a chaperone-usher operon that is silent under laboratory conditions but was shown to express when the type 1 fimbriae complex is knocked out and could promote adhesion to eukaryotic cells [160]. Exploring the idea that a substantial amount of biofilm-related expression within this microbiome environment could indeed be cryptic within laboratory environments, Korea et al. [160] identified a wide range of these chaperone-usher fimbrae as associated with distinct surface specialties in *E*. *coli* K-12.

The contigs assembled here from root samples and upregulated in contaminated conditions did indeed represent the majority of these previously identified cryptic chaperone-usher fimbriae, including *yfc* (c601154_g1_i3; *yfcRQOU*(usher papC)*SP*(chaperone papD)B(prmB)aroC), *sfm* (c575401_g1_i2; *sfmCD*); *fim* (c600702_g6_i3; *fimDFGH*), *yad* (c601794_g1_i1; *yadHG,* c601794_g2_i3; *yadCKLMhtrEecpD* and c601794_g3_i1; *yadECDI*), *yde* (c591908_g2_i3; *ydeTQSR*), *yqi* (c601683_g14_i1; *yqiGHI*), *yeh* (c598114_g3_i1; *yehABC*) and *yra* (c600252_g5_i1; *yraJ*) (Fig. 7 and Additional file 10)*.* These represent putative operons, predicted in *E*. *coli* K-12, which do not contribute to adhesion of *E*. *coli* under normal laboratory conditions but do promote biofilm formation on uncommon abiotic surfaces (‘unknown environmental niches’ [160]) and do contribute to adhesion to eukaryotic surfaces. The ELF operon (*elfADCG*) [179] was observed within a single contig as the seven member *elfADCG-ycbUVF* operon (c601320_g2_i5) predicted in *E*. *coli* K-12 with an additional protein, *pyrD* dihydroorotate dehydrogenase gene (quinone). Interestingly, a number of proteins expressed within these contigs are suggestive of potential functionality of association with a host: *ydeTQSR* included a serine/threonine-protein kinase HipA and the toxin component of a HipA family toxin/antitoxin system, and pantothenate synthetase, 3-methyl-2-oxobutanoate hydroxymethyltransferase and aspartate 1-decarboxylase (both of which participate in pantothenate and coA biosynthesis) were expressed along with *yadECDI*.

In addition to these extra-laboratory expressed operons associated with biofilm formation, a number of DE genes were associated with swarming in *Pseudomonas aeruginosa*, a process strongly related to biofilm formation. Four of the six genes explored as swarming negative mutants by Overhage et al. [180] were necessary for good biofilm formation; all were DE here and in higher abundance in contaminated conditions: *rhlE* (c601160_g1_i1, ATP-dependent RNA helicase), *lptA* (c602252_g5_i2, lipopolysaccharide transport periplasmic protein), *gshB* (c586692_g1_i1, glutathione synthetase) and *acsA* (c600318_g2_i1, acetyl-CoA synthetase). Additionally, two variants of *traABCEKLNPUVWYtrbCL* (c582511_g1_i6 and c582511_g1_i7) are comprised of conjugative apparatus (pili construction) common to F-like plasmids and necessary for mobility of genetic elements between bacteria (Fig. 7 and Additional file 10). None of the bacterial secretion systems are thought to be constitutively expressed but are instead triggered by recognition of host molecular pattern through adhesins [181, 182]. As discussed with relation to chitinase secretion, *gspAB* and *gspCDO* (with *chiA*) of the type II secretion system were upregulated in contaminated conditions. Given this pattern, it would be expected that *xcpU* (the general secretion pathway protein H, *gspH* in *E*. *coli*) would be DE; however, it was not identified as such.

The common pilus (ECP) operon is widely conserved throughout *E*. *coli* and required for early-stage biofilm development and host cell recognition [183], although usually silent in *E*. *coli* K-12 MG1655 under laboratory conditions [184]. As discussed above, an operon containing the regular common pilus machinery, *ecpRABCDEykgJyagU* (c589067_g3_i3; synonyms include but are not limited to *matABCDEFykgJyagU*) was in higher abundance under contamination conditions. Interestingly, the *yagTSRQ* operon (c589067_g1_i4; synonym *paoABCD*), common in *E*. *coli* K-12 strains, was also DE, encoding periplasmic detoxification enzymes (xanthine oxidase family [185]) including yagT, a twin arginine translocator (tat) pathway signal sequence. While the secYEG translocon can transport unfolded proteins, the tat can transport folded proteins to the periplasm (around 6% of all secreted proteins are thought to be tat dependent [186]). These secreted proteins include hydrogenases, dehydrogenase and nitrate reductases. The tat-dependent hydrogenase-2 operon (redox) (*hybGFEDCBAO*; c600034_g1_i12) was identified as DE, including the Tat pathway signal sequence domain protein *hybA* [187].

Curli fimbriae are outer membrane adhesins employed by *E*. *coli* to facilitate biofilm adhesion [177, 188, 189]. A master regulator of biofilm formation, *csgD* [189], is at the centre of the curli fimbriae regulatory network. Two variants of the *csgD* containing operon *csgDEFGycdXYZ* (c596842_g1_i2 and c596842_g1_i4) were DE and in higher abundance in contaminated roots (Fig. 7 and Additional file 10). *csgDEFG* encode four proteins necessary for curli assembly: *csgG* is involved in pore formation in the outer membrane, whereas *csgE* and *csgF* are periplasmic proteins which physically interact with *csgG* [188]. *csgD* is a positive regulator of *csgAB* (classically expressed on a distinct operon), the major curli extracellular structural subunit (*csgA*) and its nucleator (*csgB*). While *csgAB* was not identified as DE here, it has been recognised that this is often the case (*csgAB* not being *expressed* in cells forming curli) and that assembly can occur via interbacterial complementation, it may also be true here that subunit production is not transcript dependent or is constitutive and constant (and so not DE). Additionally to curli, the prominent surface protein *flu* was also upregulated (c601850_g2_i6), a self-recognising adhesin known to induce autoaggregation to promote biofilm formation [190].

*Nitrogen management*

Similar to the recently sequenced plant growth-promoting endophyte *Enterobacter* sp. 638, which was isolated from poplar stems, it seems likely that no nitrogen fixation was occurring within the *Enterobacteriaceae* species responding to contamination as no *nif* genes were identified as DE, with the exception of the pyruvate-flavodoxin oxidoreductase (*nifJ/ydbK*) (Fig. 7 and Additional file 10). Two variants of the fixABCX operon were also upregulated (c602208_g1_i1 and c602208_g1_i3; intricately linked with N fixation in diazotrophs [191, 192]), but in *E*. *coli*, these genes form an important reducing role in carnitine metabolism [193, 194].

Differential expression of well-characterised respiratory nitrate reduction (nar) operons were identified including *narK* (c598952_g5_i3) used for nitrate/nitrite extrusion in *E*. *coli* [195]; *narUZ* (c598132_g7_i1) where *narU* is also used for nitrate/nitrite extrusion while narZ is part of nitrate reduction, as well as *narXLychONChaC* (c598952_g3_i7; similar to 282397 (TU0650)), nitrate/nitrite sensor and regulator proteins. Two slightly divergent versions of the periplasmic nitrate reduction (nap) operon was also DE, *napABCGHccmABCFE* (c602069_g2_i2 and c602069_g2_i4; similar to 425132 (TU1418)) [196]. The important nitric oxide reduction (dissimilatory reduction) equipment in *E*. *coli*, via flavorubredoxin expression encoded by the *NorVW* operon [197], was also DE. The nitrogen assimilation control regulator *nac* was also present within an upregulated operon ((c598923_g2_i7; common to in structure to 580938 in *E*. *coli* BL21(DE3)) containing the *cbl* regulator, involved in aliphatic sulfonate utilisation, and *yeeO* MATE efflux transporter (involved in flavin secretion). In terms of ammonia generation, two versions of anaerobically expressed nitrite transport and reduction *nir* operons [31, 198] were DE: *nirBtsgA* (c598930_g2_i1) and *nirBCDcysG* (c598930_g1_i1). Amino acid (glutamine) synthesis under nitrogen demand has also been shown to be fine-tuned (ammonia/ammonium) by the nitrogen assimilatory protein *glnK* regulation of *amtB* (ammonium transporter) [199], present here within an upregulated operon containing the *amtB* and two multidrug efflux proteins, *mdlAB*, (c602197_g2_i5; all commonly expressed together). Additionally to this, *nrfA*, a cytochrome c-552 which catalyses nitrite to ammonia in a formate-dependent manner, was upregulated (c600318_g2_i1) as well as an operon containing *yddG*, an aromatic amino acid extrusion protein, in a operon (c601567_g3_i3) containing the nitrate-inducible formate dehydrogenase major subunit *fdnG*.

The conversion of inorganic nitrogen, through nitrate assimilation and reduction, to the biologically more useful nitrite and ammonia (for example, to construct amino acids) provides bacteria with nutrition for survival within the environment but also a potential currency for mutualism within the microbiome, or mycorrhizosphere, system. *E*. *coli* only assimilates nitrate under anaerobic conditions [200]; this broad spectrum of assimilatory and dissimilatory nitrate reduction equipment with higher abundance under contaminated conditions is perhaps unsurprising given that facultative anaerobic *Enterobacteriaceae* can outcompete obligate anaerobes in anoxic, organic compound-rich forest soils [201].

*Bacterial functional role*

So-called mycorrhizal helper bacteria MHB [30] are thought to promote the symbiotic association between plant roots and mycorrhizae, creating a tripartite relationship [29], such as the *Bacillus sphaericus* isolate *EJP109* promoting *Suillus luteus* (ECM) growth in association with *Pinus sylvestris* as well as the *Streptomyces* sp*.* nov. 505 and *S*. *anulatus* (Beijerinck) Waksman 1003 promoting *Amanita muscaria* (ECM) growth in association with *Picea abies* [172, 202]. MHB and PGPR (plant growth-promoting rhizobacteria) strains of *Pseudomonas* have been visualised attaching to ECM hyphae (suggesting extracellular biotrophic mycophagy) of the genus *Laccaria*, in a bacterial and fungal strain-specific manner [203]. Differentiating between the potential roles of interacting bacterial species within the biological system is difficult; Leveau and Preston [204] suggested three potential modes of interaction, necrotrophy, extracellular biotrophy and endocellular biotrophy, as well as a spectrum of expected functionality associated to each mode. In general here, expression suggested extracellular biotrophy, however, not only are the other two modes of interaction possible but a more complex continuum of interaction would seem likely given the diverse environment. Before such functionality can be confidently elucidated, in addition to the necessary promotion of cross-disciplinary microbiome research, observation of gene expression without constraint to an organism/s expected within a biological system needs to become a standard requirement for transcriptomic investigations.

## Conclusions

From gene expression alone, the major responses of *Salix purpurea* cv. ‘Fish Creek’ to soil contamination do not seem to be direct degradation, immobilisation or exclusion of contaminants but rather widespread, extraordinarily complex, alterations to interactions with microbiota. The extremely diverse fungal community, revealed using a bioinformatics approach unconstrained by the requirement of *a priori* nucleotide sequence, responds dynamically to contamination both with changes to gene expression but also with substantial shifts in the community makeup. Much like the host plant, fungal gene expression was dominated by alterations in pathways associated to microbiome interactions as opposed to direct hydrocarbon degradation. Surprisingly, gene expression representing increased petroleum hydrocarbon metabolism equipment came from an *Enterobacteriaceae* species whose total expression was uniformly more abundant in roots of contaminated trees. It seems possible that, given the successful contamination tolerance, willows may depend on symbiosis to tolerate stressful environmental conditions instead of relying solely on their own metabolism.

While polyadenylated bacterial sequences are often not quantified across experimental systems investigated with eukaryote expression in mind, the bacterial functionality observed here is thought-provoking, revealing a plausible set of expressed genes indicating tripartite mutualism. A finding which is useful in providing a convincing reminder that observing the entirety of generated data, even that which is commonly discarded, is always of potential value for exposing the unexpected. Most importantly, with regard to the attempted all-inclusive strategy of observing all expressed sequences within a biological sample, regardless of origin, is how extensively interpretation of expression could be confounded if plant, fungal or bacterial expression were investigated as responsive to treatment (here being contamination) in isolation, and thus how crucial it is to attempt observation of the entire microbiome and wider metaorganism. This metatranscriptomic approach, which marries an understanding of the uncertainty of microbiome biology with the strengths and limitations of current bioinformatics, should be broadly transferable through utility for biological samples containing expressed sequences from well-characterised, poorly characterised or entirely unknown organisms at a diversity of low or high complexity.

## Additional files

Additional file 1: Unknown sequence challenge, upregulated DE *Basidiomycota* blastn and additional transcriptomic methodology [2, 36, 46, 52–57, 205–224]. (DOCX 331 kb)

Additional file 2: Custom scripts. (DOCX 133 kb)

Additional file 3: Unknown DE spreadsheet. (XLSX 2375 kb)

Additional file 4: Total annotation spreadsheet. (XLSX 65570 kb)

Additional file 5: Salix DE spreadsheet. (XLSX 2169 kb)

Additional file 6: Fungi DE spreadsheet. (XLSX 6048 kb)

Additional file 7: Upregulated DE *Basidiomycota* spreadsheet. (XLSX 1898 kb)

Additional file 8: Upregulated DE *Basidiomycota* blastn spreadsheet. (XLSX 958 kb)

Additional file 9: Total DE spreadsheet. (XLSX 7230 kb)

Additional file 10: Bacteria DE transdecoded (annotation of polycistronic contigs) spreadsheet. (XLSX 2104 kb)
