# Supplementary material for: Trees, fungi and bacteria: tripartite metatranscriptomics of a root microbiome responding to soil contamination
Source: Microbiome. 2018 Mar 21;6:53. doi: 10.1186/s40168-018-0432-5 (PMC5863371; doi:10.1186/s40168-018-0432-5)
Supplement: Supplementary file 1 — Unknown sequence challenge, upregulated DE Basidiomycota blastn and additional transcriptomic methodology [2, 36, 46, 52–57, 205–224]. (DOCX 331 kb) [file 40168_2018_432_MOESM1_ESM.docx]

## Supplementary file 1

**Contents**

## Unknown DE contigs

## Upregulated differentially expressed (DE) *Basidiomycota* blastn

## Additional transcriptomic methodology

## Alternative MA plot

## Unknown DE contigs

The separation of contig assembly from contig annotation in *de novo* strategies (when annotated without constraint to a single organism alone) allows for strict assembly criteria but more inclusive annotation. This, vitally, provides confidence in both contig presence within the system and DE with relevance to the biological treatment while, independently, allowing for increased identification of the unexpected through the forgiving blastx (it being essential to provide as much biological information as possible within the highly uncertain environments of eukaryotic tissue). Even using this strategy, a fifth of the treatment responsive transcripts within the RNA samples (DE contigs) were unknown, bearing no resemblance to known proteins. These unknown contigs are routinely discarded or unreported in many bioinformatics strategies and pipelines.

To further investigate these highly abundant unknown contigs within the system, we first extended analysis to include blastx against UniParc, the largest non-redundant protein sequence database (but with no annotation). An additional 160 sequences, previously unknown, returned hits from uniparc and were integrated with the rest of the annotated DE contigs for contribution to a wider picture of species composition and functional understanding of the biological system. The remaining 2575 contigs are retained as unknown (Figure 2 and Supplementary file 9) but were compared against NCBI expressed sequence tag (EST) database, ncRNA (NONCODE database) and the *Salix purpurea* 94006 genome sequence (non-coding scaffold). Querying of the EST database revealed 762 additional hits (e-value < 1e^-4^; parameters : gapopen 5, gapextend 2, word_size 7), ncRNA revealed 15 hits (e-value 1e-10 ; parameters: gapopen 5, gapextend 2, word_size 7) and the *Salix purpurea* 94006 genome sequences (criteria: e-value 1e-10 ; parameters: gapopen 5, gapextend 2, word_size 7) revealed 225 hits (supplementary file 9).

The most abundant contig of all DE contigs was unknown (c568138_g3_i1) and was in higher abundance in contaminated roots (35,275 tpm in treatment, 56 tpm in control). A very highly abundant sequence, such as this, with no similar annotated homolog within the major sequence repositories could be considered an assembly error, although the treatment specific expression makes this unlikely. c568138_g3_i1did have an unannotated EST hit (identity 87.86%, e-value 5.00e-61, coverage 94.81%); interestingly the hit derived from metatranscriptomic study sampling heavy metal contaminated soil from a *Pinus* forest stand in Belgium [205]. Given the similarity of conditions (albeit from studies on different continents) and eighty five other unknown contigs which also had high confidence unannotated EST hits specifically from this Belgian study (including the second most abundant contig, 8502 tpm in treatment, 32 tpm in control), it seems highly likely that these contigs represent important and novel transcripts common to both studies which are unknown to science, and thus merit further study. Out of the 762 EST hits, over 350 were unannotated sequences sampled from recognisable plant root studies (many derived from unpublished/unknown sources and a minor number derived from other tissues). Most of these studies involved stressful conditions in: poplar (saline-arid [206], unstressed [207, 208], drought-stressed [209], biotic interaction [210]) and pine (calcium, metal, nutrient, chemical, saline and drought-stressed) but also from oak (no information), chickpea (drought and saline-stressed [211]), sugarcane (drought and fungal interaction [212], *Kashgar tamarisk* (saline-stressed [213]) and wheat (boron-stressed). The entirety of NCBI EST database was queried with the unknown DE contigs, so the prevalence of plant root experiments dominating returned hits is informative in rendering technical assembly error highly unlikely. Additionally, EST sequences were not identified as plant but rather originate from a similar environment of plant tissue (predominantly tree roots), and so could derive from other organisms. As well as unknown DE contigs being similar to unknown ESTs from root samples, over 50 contigs had hits recognisable as EST deriving from mycorrhizal fungi, including from *Hebeloma* (plant-fugal interaction study [214]), *Gibberella* (plant-fugal interaction study [215]), *Fomitopsis* (plant-fugal interaction study [216]), *Tuber, Laccaria* and *Gloeophyllum*. As the unknown DE contigs (representing a fifth of all DE contigs) closely matched the expression pattern of recognised fungal DE transcripts (figure 2) the evidence would suggest that a substantial proportion of these sequences represent uncharacterised fungal sequence and therefore we would caution prudence when describing ESTs as originating directly from the (plant) tissue sampled.

Non-coding RNA (ncRNA, potentially functional RNA) is overlooked in this annotation strategy, so could represent the substantial shortfall in sequence recognition. Research into ncRNA is rapidly progressing [217, 218] yet is still somewhat in its infancy in terms of utility for prediction in non-model organisms. Here, only 15 DE contigs had high confidence hits in ncRNA database (supplementary file 9). The NONCODE database is limited to 16 species including only one plant species (*Arabidopsis*). The hits here originated from yeast, gorilla, human, mouse and *Drosophila*. To further query the unknown sequences they were also compared directly to the *S. purpurea* 94006 genome (blastn against those regions not predicted as protein coding). A total of 225 DE contigs had high confidence hits within the *S. purpurea* 94006 genome. These hits could potentially represent as-yet uncharacterised ncRNA or protein coding regions not successfully predicted by annotation software (excluding intron containing genes), although this is unlikely as the sequences have no similarity to any protein sequences available in the major protein repositories. Although current consensus considers the majority of higher eukaryotic genomes may constitute ncRNA, due to the high level of uncertainty when exploring non-genic transcription [219] (particularly in non-model organisms), polarised opinions exist in the field. These include those strongly championing the overwhelming prevalence of “junk” RNA [219, 220]; however, in place of a direct proof of functionality (as is currently the standard in metatranscriptomic studies), it would seem logical to at least allow for the possibility that differentially expressed ncRNA *could* be functional.

Current estimates suggest that there are approximately 11 million distinct species that exist globally [221]; of these, only around the order of 0.001% have been genome sequenced and annotated as of April 2013 [222] (NCBI has currently has 17,104 genomes sequences representing distinct species or strains as of Oct 2016). The estimated proportion of plant species having been sequenced and annotated is of the order of 0.01%. It would therefore seem prudent to consider RNA-seq data interpretation methodologies that are designed to derive useful information from the 99.99% of non-model organisms containing the majority of (unknown) genetic information in the biosphere.

DE contigs which couldn’t be annotated were explored here through comparison against potential non-coding RNA, non-coding genomic sequence/scaffold and by searching for similarity to other unknown sequences. We hope that the results of this type of relentless pursuit of any potentially relevant sequence information, in contrast to discarding the unknown, can reveal a powerful roadmap for future research; here the most highly expressed contigs were unknown but were recognised as having previously been identified in the mycorrhized roots of similar experiments conducted in Belgium.

## Upregulated differentially expressed (DE) *Basidiomycota* blastn

Gene sequencing technologies have revealed microbiome community complexity which was previously obscure to culturing techniques; however, positively identifying organisms present in complex microbiome systems is still non-trivial. An important hurdle to this positive identification of distinct life within the standard menagerie of a microbiome is that 99.99% of species (estimated [221, 222]) have yet to have had even a single genome from the species sequenced and annotated as a representative. In short, the vast majority of life has yet to be characterised in genetic terms. Methods using 18S/ITS and 16S ribosomal RNA (rRNA) barcoding are most commonly used for species identification where the power of the technique comes from the recognition of known sequences from known strains/accessions expected within a biological sample. However, quantitative community assessment can be problematic for samples containing an unknown mix of organisms [52-57], as is potentially the case with those taken from any biological environment without strong artificial selection. This is due to a number of fundamental factors which can potentially confound 18S/ITS and 16S rRNA barcoding methodology and limit accurate quantitative community assessment, including: 1) amplicon length restrictions is common to a number of barcoding pipelines predicated on amplified hyper-diverse rRNA regions not containing inserts extending the amplicon beyond an assumed length or deletions beyond applied gap penalties (the degree to which community assessment is confounded by this can be assessed using amplicon length analysis alongside quantification of read mapping rates, unfortunately, these are rarely reported). 2) The current limitation of database scale and accuracy (although barcode databases are increasing in scale at a rapid rate). 3) Amplicon design (primer selection) beyond read length is common when using shorter read length sequencing technologies, the resulting commonality of short overlapping regions could substantially increase individual read mapping opportunities to artificial contigs and therefore dilute accurate abundance assessment. 4) Most importantly, the requirement for primer binding; no universal primers exist and ultra-conserved regions are not always conserved (unamplified regions cannot be quantified and organisms represented by amplified regions are normalised to 100% in a given sample, potentially confounding quantitative assessment and, importantly, proportional shifts in community make-up if treatments are being compared).

When targeting gene differential expression in response to biological factors, much like traditional culturing methodology, this inability to quantify the potentially substantial proportion of unamplified and poorly mapped organisms (read mapping rates of sequenced amplicons are often <50%) confounds comparisons between samples of complex and dynamic systems as relative relationships within different treatment communities are lost [223]. Instead of fingerprinting/barcoding, a top-down approach was employed which is not predicated on *a priori* certainty of nucleotide sequence present in extra-laboratory samples containing an unknown community make-up. The cost here is the loss of positive identification of the known, highly characterised organisms within the community based on barcode sequencing, with the gain of capturing a greater proportion of functional complexity of the biological system which allows for the unexpected [2, 46].

This was most problematic in *Basidiomycota* where only a very small proportion of transcripts could be annotated from a single organism, indicative of either widespread conservation across whole genomes of *Basidiomycota* (which is unlikely) or that the organism or the few organisms dominating the response to contamination are included in the 99.99% [222] of unsequenced fungi without a distinctively close relative at family level. Out of the 1745 *Basidiomycota* contigs upregulated in contamination treatment, 40 contigs could be annotated from a single organism only, comprising 23 species: 4 brown rot saprotrophs (*Coniophora puteana* RWD-64-598; 2x *Fibroporia radiculosa*; 2x *Fistulina hepatica* ATCC 64428; *Gloeophyllum trabeum*), 10 white rot saprotrophs (*Galerina marginata* CBS 339.88; *Heterobasidion irregulare* TC 32-1; *Jaapia argillacea* MUCL 33604; 3x *Phlebiopsis gigantea* 11061_1 CR5-6; *Pleurotus ostreatus* PC15; 3x *Punctularia strigosozonata* HHB-11173 SS5; *Pycnoporus cinnabarinus*; *Schizopora paradoxa*; 5x *Stereum hirsutum* FP-91666; *Trametes versicolor* FP-101664), one litter decaying saprotroph (*Gymnopus luxurians* FD-317 M1), six ECM biotrophs (4x *Laccaria amethystina*; 2x *Laccaria bicolor*; 3x *Piloderma croceum* F 1598; *Pisolithus tinctorius* Marx 270; *Scleroderma citrinum* Foug A; *Suillus luteus* UH-Slu-Lm8-n1) and the orchid symbiote *Tulasnella calospora* (MUT 4182).

The broad pattern of annotation for these upregulated *Basidomycota* transcripts, highly distinct from the rest of the fungal, plant or bacterial contigs where organisms were more confidently identified due to high (independent) representation (over 500 hits, supplementary file 3). This could potentially be explained by the presence of an uncharacterised species or even a species within an uncharacterised *Basidiomycota* family (as was the scenario in the recently identified *Basidiomycota* symbiotes of Lichen [224]). To further query this supposition, we also annotated all DE contigs using BLASTn. Nucleotide comparison is inherently stricter, so while it is less useful for revealing functionality within extra-laboratory data including uncharacterised genetics, it could provide greater certainty for identification of well characterised species. We imagined that diverse fungal species all differentially expressing a limited number transcripts would be represented by a (reduced) number of high confidence nucleotide hits across the spectrum of the species whereas an unknown fungi would result in a severely reduced number of high confidence nucleotide hits in the closest related fungal relative. Of the total 1745 upregulated *Basidiomycota* contigs of interest successfully annotated using blastx, only 11 (0.6%) could be annotated by nucleotide BLAST against NCBI nt database (wordsize 50), identified as: uncultured eukaryote clone, *Acrasis rosea* (calmodulin gene), *Stereum hirsutum* (histone-fold-containing protein), *Antrodia xantha*, Uncultured *Basidiomycota* clone/ECM fungus (*Thelephoraceae*), *Pulcherricium caeruleum* (rRNA), *Penicillium expansum* (ATPase), *Peniophora* sp. (rRNA), *Homo sapiens* BAC clone, Uncultured bacterium clone (but with low coverage of 15%), *Tricholoma matsutake* (mitochondrial genomic). The three putative protein functions corresponded returned by blastn here matched those identified in the translated annotation performed throughout this research, the other eight hits were highly ambiguous sequences.

Seemingly, the origin of the upregulated *Basidiomycota* sequences, identified here as essential for understanding the metatranscriptomic response to contamination, could not have been discerned using strict constriction to known nucleotide sequence, a realisation which is also of potential value to study of extra-laboratory gene expression in other biological contexts. Most of the BLASTn annotation of DE contigs were comprised of *Salicaceae* (642 contigs or 46.4%, although the vast majority of these were identified as from poplar instead of *Salix*) and various *E. coli* species (488 contigs or 35.3%). While organism identification is important (sequences are deposited in the ENA for use as a more substantial number of fungal genomes are sequenced), perhaps equally important, is transcript function and whether this treatment specific group of transcripts can inform us as to the fungal ecological role and therefore the biology behind phytoremediation.

## Additional transcriptomic methodology

### RNA Extraction and Illumina Sequencing

Species: *Salix purpurea* 94006

Organ: Roots

Samples: 12 per seq run (6 non-contaminated and 6 contaminated), 48 in total (4 runs).

Non-contaminated: 122, 124, 128, 149, 154, 155

Contaminated: 27, 29, 33, 38, 42, 78

Library source: RNA

Library type: mRNASeq

Type of sequencing: Illumina HiSeq 2500

Adaptor: TruSeq RNA

Library Layout: PAIRED (150 bp)

Total number of reads: 527,029,606

Total number of base pairs: 158,108,881,800

Compared raw library sizes (variance represented around 1):

| 122 | 124 | 128 | 149 | 154 | 155 | 27 | 29 | 33 | 38 | 42 | 78 |
| --- | --- | --- | --- | --- | --- | --- | --- | --- | --- | --- | --- |
| 0.74 | 0.84 | 0.71 | 0.53 | 1.05 | 1.22 | 0.76 | 0.94 | 0.49 | 1.51 | 1.49 | 1.73 |

### Quality control

Data were filtered using Trimmomatic [36] to trim poor quality nucleotides at the beginning and the end of each sequence. Reads shorter than 40 bp and orphaned reads after quality control were removed from the pool.

Parameters: LEADING:15 TRAILING:15 SLIDINGWINDOW:5:15 MINLEN:40

Post QC statistics:

Total number of reads: 456,182,049

Samples: 12 (6 non-contaminated and 6 contaminated).

Compared raw library sizes (after quality control):

| 122 | 124 | 128 | 149 | 154 | 155 | 27 | 29 | 33 | 38 | 42 | 78 |
| --- | --- | --- | --- | --- | --- | --- | --- | --- | --- | --- | --- |
| 0.74 | 0.85 | 0.66 | 0.52 | 1.04 | 1.24 | 0.76 | 0.91 | 0.48 | 1.57 | 1.50 | 1.72 |

### *De novo* Assembly

| Total length of contigs(bp) | 103,255,770 |
| --- | --- |
| Total number of contigs | 189,849 |
| N50 (bp) | 714 |
| GC% | 49.2 |

### Abundance

| Tree Number | Overall alignment rate (%) | Mapped raw counts | Compared count sizes |
| --- | --- | --- | --- |
| 122 NC^1^ | 66.43 | 3,324,466 | 0.54 |
| 124 NC | 69.93 | 6,112,399 | 0.99 |
| 128 NC | 64.02 | 2,958,805 | 0.48 |
| 149 NC | 67.25 | 1,989,874 | 0.32 |
| 154 NC | 64.85 | 7,358,421 | 1.19 |
| 155 NC | 63.47 | 8,880,524 | 1.44 |
| 27 C | 65.36 | 3,156,236 | 0.51 |
| 29 C | 61.73 | 7,812,236 | 1.27 |
| 33 C | 63.58 | 1,889,375 | 0.31 |
| 38 C | 64.68 | 11,948,513 | 1.94 |
| 42 C | 66.24 | 9,967,495 | 1.61 |
| 78 C | 61.33 | 8,674,221 | 1.41 |

1. NC: non-contaminated soil; C: contaminated soil.

### 3.5 Differential Expression and MA-plot generation.

We used EBSeq, an empirical Bayes model to identify differentially expressed genes and isoforms. Prior to using EBSeq functions, raw counts were normalized based on between sample normalization Median Normalization (see main text for references). The main EBSeq script faithfully follows the EBSeq Bioconductor manual and is reproduced in supplementary file 10 (see: EBSeq script to calculate differentially expressed contigs).

MA-plots were generated using edgeR based on between sample normalization TMM (see main text for references). The script is reproduced in supplementary file 10 (see: MA-plot script used for figure2). It should be noted that at no point were any statistics derived from edgeR program.

##### Typical commands used

**Trimmomatic**: java -classpath trimmomatic-0.33. org.usadellab.trimmomatic.TrimmomaticPE -phred33 readFile_R1.fastq readFile_R2.fastq readFile_R1_paired.fastq readFile_R1_unpaired.fastq readFile_R2_paired.fastq readFile_R2_unpaired.fastq LEADING:15 TRAILING:15 SLIDINGWINDOW:5:15 MINLEN:40

**Trinity normalization**: normalize_by_kmer_coverage.pl --seqType fq --JM 120G --max_cov 30 --left readFile_R1.fastq –right readFile_R2.fastq --pairs_together --JELLY_CPU 24

**Trinity assembly**: Trinity.pl --seqType fq --JM 120G –left concatenatedNormalizedReads_R1.fastq.normalized_K25_C30_pctSD100.fq --right concatenatedNormalizedReads_R2.fastq.normalized_K25_C30_pctSD100.fq --CPU 24 --bflyCPU 24

**Bowtie2**: bowtie2 -a -X 600 -x bowtie2_Index -1 readFile_R1.fastq -2 readFile_R2.fastq | samtools view -Sb - > readFile.bam

**eXpress**: express -o xprs_readFile_out transcriptome.fasta readFile.bam

**EBSeq**: (See supplementary file 10 for EBSeq.R script)

Rscript EBSeq.R rawCounts.txt Genes_Isoform_mapping_file.txt outputFolder

**edgeR**: : (See supplementary file 10 for MAPlot.R script)

Rscript MAPlot.R outputFolder rawCounts.txt Bacteria_DEIsoList.txt Fungi_DEIsoList.txt Metazoa_DEIsoList.txt Viridiplantae_DEIsoList. Unknown_DEIsoList.

Input files available at: <https://github.com/gonzalezem/Tripartite_Metatranscriptomics_article/tree/master/supplementary_Files/EBSEQ_input_files>

## Alternative MA plots


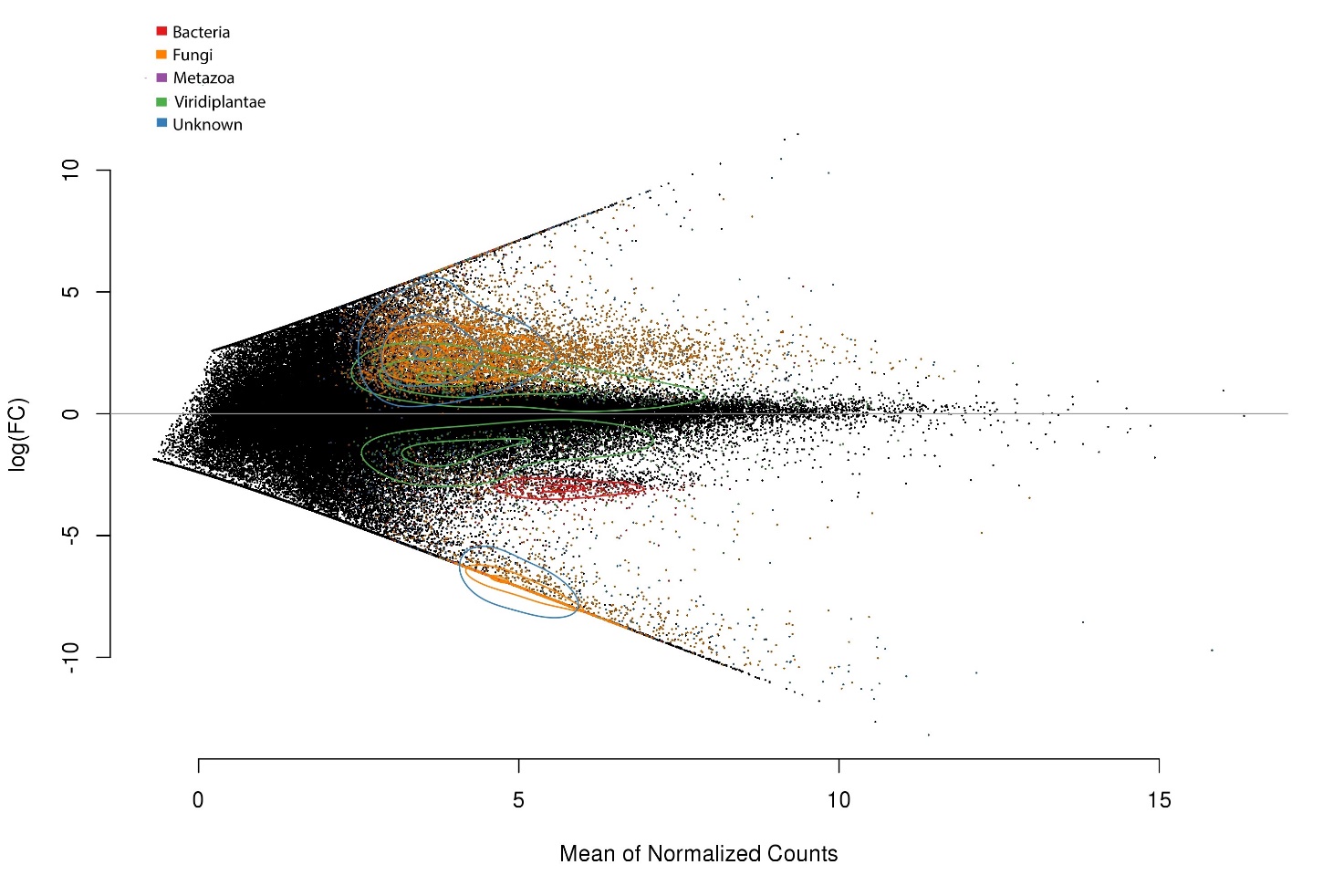


**Origin of differentially expressed contigs using EBSeq.** MA plot of *de novo* assembled transcriptome; y-axis represents fold change (FC, log_2_) between contaminated (-ive) and non-contaminated conditions (+ive) [this is the reverse of the figure used in the manuscript], and the x-axis represents the mean of normalised counts. Only annotated DE contigs are coloured by annotation, including contours to represent contig density relative within each group. EBSeq’s default Median Normalisation was used.
