# Supplementary material for: Trees, fungi and bacteria: tripartite metatranscriptomics of a root microbiome responding to soil contamination
Source: Microbiome. 2018 Mar 21;6:53. doi: 10.1186/s40168-018-0432-5 (PMC5863371; doi:10.1186/s40168-018-0432-5)
Supplement: Supplementary file 2 — Custom scripts. (DOCX 133 kb) [file 40168_2018_432_MOESM2_ESM.docx]

# EBSeq script to calculate differentially expressed contigs

Note1: this script also outputs DE contigs raw mean counts, output table, an MA-plot and a raw vs. posterior fold change plot.

Note2: EBSeq uses DESeq between sample normalization (or median ratio normalization).

---- R SCRIPT ---

library(blockmodeling)

library(ggplot2)

library(EBSeq)

library(GGally)

args = commandArgs(trailingOnly = TRUE)

#arg1: count matrix

#arg2: Isoform map file in the following format

#arg3 : full directory tree (as to where the output files should be saved) (ex : /data/Myfolder )

CountTable <- data.matrix(read.table(file =sprintf("%s",args[1]), header=T, row.names=1, com=''))

colnames(CountTable) <- c('NC1','NC2','NC3','NC4','NC5', 'NC6','C1','C2','C3','C4','C5','C6')

Treatment = CountTable[,c(7:12)]

T_MeanCounts = rowMeans(Treatment)

Control = CountTable[,c(1:6)]

C_MeanCounts = rowMeans(Control)

TCMean=cbind(T_MeanCounts,C_MeanCounts)

IsoList <- read.table(file =sprintf("%s",args[2]))

IsoNames=IsoList[,c(2)]

IsosGeneNames=IsoList[,c(1)]

IsoSizes=MedianNorm(CountTable)

NgList=GetNg(IsoNames, IsosGeneNames, TrunThre=3)

IsoNgTrun=NgList$IsoformNgTrun

Conditions = as.factor(c(rep("NC", 6), rep("C", 6)))

Conditions

IsoEBOut=EBTest(Data=CountTable, NgVector=IsoNgTrun, Conditions=Conditions,sizeFactors=IsoSizes, maxround=5)

IsoPP=GetPPMat(IsoEBOut)

IsoPP_mean_cont = cbind(IsoPP, TCMean[match(rownames(IsoPP), rownames(TCMean)), ])

#Differentially expressed isoforms

DEfound=rownames(IsoPP)[which(IsoPP[,"PPDE"]>=.95)]

DEIsoPP= IsoPP[match(DEfound, rownames(IsoPP)), ]

DEIsoPP_mean_count = cbind(DEIsoPP, TCMean[match(rownames(DEIsoPP), rownames(TCMean)), ])

DEIsoCounts= CountTable[match(DEfound, rownames(CountTable)), ]

write.table(DEIsoCounts,file =sprintf("%s/DEIso_Counts.txt",args[3]),sep = "\t")

IsoFC=PostFC(IsoEBOut)

IsoFC$Regulation[IsoFC$RealFC < 1 ] <- "Control"

IsoFC$Regulation[IsoFC$RealFC > 1 ] <- "Treatment"

write.table(IsoFC,file =sprintf("%s/Iso_FC.txt",args[3]),sep = "\t")

IsoFCData <- read.table(file =sprintf("%s/Iso_FC.txt",args[3]))

DEIsoFC = IsoFCData[match(DEfound, rownames(IsoFCData)), ]

Iso2=as.data.frame(DEIsoFC[c(2,4)])

Iso3 = cbind(Iso2, TCMean[match(rownames(DEIsoPP), rownames(TCMean)), ])

Iso4=cbind(Iso3, IsoPP[match(rownames(DEIsoPP), rownames(IsoPP)), ])

Iso5=as.data.frame(Iso4[c(1:4,6)])

Iso5[,3] = round(Iso5[,3])

Iso5[,4] = round(Iso5[,4])

write.table(Iso5,file =sprintf("%s/DeIso_data.txt",args[3]),sep = "\t")

onlyDEIso_counts <- CountTable[DEfound,]

basemean=as.matrix(log(rowMeans(IsoEBOut$DataNorm),2))

log2FC=as.matrix(log(IsoFC$PostFC,2))

jpeg(sprintf("%s/EBSeqMAPlot.jpg",outputFolder),

width = 3000, height = 2000, pointsize = 12, bg = "white", quality = 100, res=300)

plot(basemean, log2FC, cex=0.2, pch=16,

xlab="Mean of Normalized Counts", ylab="log(FC)",

frame.plot=FALSE)

abline(h = 0, col = "gray60")

logFC.tag=log2FC[rownames(DEIsoFC),]

basemean.tag=basemean[rownames(DEIsoFC),]

points(x=basemean.tag,

y=logFC.tag, type="p",

cex=0.2, pch=16, col="red")

dev.off()

IsoFC=PostFC(IsoEBOut)

jpeg(filename=sprintf("%s/FC_vs_PosteriorFC.jpg",args[3]),

width = 3000, height = 2000, pointsize = 12, bg = "white", quality = 100, res=300)

PlotPostVsRawFC(IsoEBOut,IsoFC)

dev.off()

a=IsoEBOut$Alpha

b=IsoEBOut$Beta

c=IsoEBOut$P

d=cbind(a,b,c)

colnames(d)<- c("Alpha","Beta.1","Beta.2","Beta.3","P")

write.table(d,file =sprintf("%s/check-iterations.txt",args[3]),sep = "\t")

# MA-plot script used for figure2

Note1: This script uses EdgeR to generate MA-Plot

Note2: edgeR uses TMM (trimmed mean of M- values between each pair of samples) between sample normalization.

Note3: edgeR was used for visual purposes only, no statistics were derived from edgeR’s plot.

--- SCRIPT ---

library(blockmodeling)

library(ggplot2)

library(edgeR)

library(EBSeq)

library(GGally)

library(RColorBrewer)

library(scales)

library(MASS)

args = commandArgs(trailingOnly = TRUE)

#arg1 : output full directory tree

#arg2: raw count Matrix

#arg3-7: DE contig list, for Bacteria, Fungi, Metazoa, Viridiplantae, Unknown respectively

CountTable = data.matrix(read.table(file =sprintf("%s",args[2]), header=T, row.names=1, com=''))

Conditions = as.factor(c(rep("C", 6), rep("T", 6)))

#edgeR

y <- DGEList(counts=CountTable,group=Conditions)

y<- estimateCommonDisp(y)

de <- exactTest(y)

deduped.data <- unique( de$table[ , 1:2 ] )

maxlogFC=max(deduped.data$logFC)

minlogFC=min(deduped.data$logFC)

deduped.data$Colour="white"

pal= brewer.pal(8,"Set1")

pdf(file=sprintf("%s/MA_plot_5species.pdf",args[1]), width=10, height=9)

plot(deduped.data$logCPM, deduped.data$logFC, cex=0.2, pch=16,

xlab="Mean of Normalized Counts", ylab="log(FC)", col=deduped.data$Colour,

frame.plot=FALSE)

abline(h = 0, col = "gray60")

UnknownData = data.matrix(read.table(file =sprintf("%s",args[7]), header=T, row.names=1, com=''))

nrow(UnknownData)

de$genes <- rownames(UnknownData)

gene.labels <- de$table[de$genes,]

points(x=gene.labels$logCPM,

y=gene.labels$logFC, type="p",

cex=0.2, pch=16, col=alpha(pal[2],0.6))

FungiData = data.matrix(read.table(file =sprintf("%s",args[4]), header=T, row.names=1, com=''))

nrow(FungiData)

de$genes <- rownames(FungiData)

gene.labels <- de$table[de$genes,]

points(x=gene.labels$logCPM,

y=gene.labels$logFC, type="p",

cex=0.2, pch=16, col=alpha(pal[5],0.6))

MetazoaData = data.matrix(read.table(file =sprintf("%s",args[5]), header=T, row.names=1, com=''))

nrow(MetazoaData)

de$genes <- rownames(MetazoaData)

gene.labels <- de$table[de$genes,]

points(x=gene.labels$logCPM,

y=gene.labels$logFC, type="p",

cex=0.2, pch=16, col=alpha(pal[4],0.6))

PlantaeData = data.matrix(read.table(file =sprintf("%s",args[6]), header=T, row.names=1, com=''))

nrow(PlantaeData)

de$genes <- rownames(PlantaeData)

gene.labels <- de$table[de$genes,]

points(x=gene.labels$logCPM,

y=gene.labels$logFC, type="p",

cex=0.2, pch=16, col=alpha(pal[3],0.6))

BacteriaData = data.matrix(read.table(file =sprintf("%s",args[3]), header=T, row.names=1, com=''))

nrow(BacteriaData)

de$genes <- rownames(BacteriaData)

gene.labels <- de$table[de$genes,]

points(x=gene.labels$logCPM,

y=gene.labels$logFC, type="p",

cex=0.2, pch=16, col=alpha(pal[1],0.6))

legend(-1, 16, legend = expression(italic("Bacteria"),italic("Fungi"),italic("Metazoa"),italic("Viridiplantae"),italic("Unknown")),

cex=0.7, fill = c(pal[1], pal[5], pal[4], pal[3], pal[2]),

border=c(pal[1], pal[5], pal[4], pal[3], pal[2]),

box.lwd = 0, bty = "n")

#CONTOURS

de$genes <- rownames(FungiData)

gene.labels <- de$table[de$genes,]

z <- kde2d(gene.labels$logCPM, gene.labels$logFC, n=250)

contour(z, drawlabels=FALSE, nlevels=3, col=pal[5], add=TRUE, lwd=1)

de$genes <- rownames(PlantaeData)

gene.labels <- de$table[de$genes,]

z <- kde2d(gene.labels$logCPM, gene.labels$logFC, n=250)

contour(z, drawlabels=FALSE, nlevels=4, col=pal[3], add=TRUE, lwd=1)

de$genes <- rownames(BacteriaData)

gene.labels <- de$table[de$genes,]

z <- kde2d(gene.labels$logCPM, gene.labels$logFC, n=250)

contour(z, drawlabels=FALSE, nlevels=2, col=pal[1], add=TRUE, lwd=1)

de$genes <- rownames(UnknownData)

gene.labels <- de$table[de$genes,]

z <- kde2d(gene.labels$logCPM, gene.labels$logFC, n=250)

contour(z, drawlabels=FALSE, nlevels=3, col=pal[2], add=TRUE, lwd=1)

dev.off()

# Fasta parser

Returns a tab separated table with sequences identification, length, and description of a fasta file

#!/usr/bin/python

from Bio import SeqIO

from Bio.SeqUtils import GC

import os

from datetime import datetime

import argparse

import pandas as pd

from pandas import *

startTime = datetime.now()

parser = argparse.ArgumentParser(description="Returns a tab separated table with sequences identification, length, and description of a fasta file : prefix_ParsedFasta.txt. If Trinity input 2 files will be generated, see below. Note : don't use this script to parse nr proteine database fasta file. Use the script fastaparser_for-nr-fasta-file.py instead.")

parser.add_argument('-i', '--ifile', help='[REQUIRED] Fasta file', dest='inputfile', action='store', required=True)

parser.add_argument('-p', '--prefix', help='[REQUIRED] Prefix is a string that will inserted at the beginning of the outputfile name. Input file example : -p Swissprot',dest='prefix', action='store', required=True)

parser.add_argument('-t', '--trinity', help='[OPTIONAL] Subject sequences are Trinity subject sequences (e.g comp1_c0_seq1). If -t is applied, two files will be generated: one descriptive (prefix_ContigDescrp.txt) with 4 columns (contigid, ContigLength, description, and GCContent) and another one that will map isoforms to compounds/genes (prefix_Contig_Coumpound_List.txt).', dest='trinity', default=False, action='store_true')

args = parser.parse_args()

inputfile = args.inputfile

prefix = args.prefix

Trinity = args.trinity

def SimpleFastaParser(handle):

#Skip any text before the first record (e.g. blank lines, comments)

while True:

line = handle.readline()

if line == "":

return

if line[0] == ">":

break

while True:

if line[0] != ">":

raise ValueError(

"Records in Fasta files should start with '>' character")

title = line[1:].rstrip()

lines = []

line = handle.readline()

while True:

if not line:

break

if line[0] == ">":

break

lines.append(line.rstrip())

line = handle.readline()

yield title, "".join(lines).replace(" ", "").replace("\r", "")

if not line:

return

assert False, "Should not reach this line"

def batch_iterator(iterator, batch_size) :

entry = True

while entry :

batch = []

while len(batch) < batch_size :

try :

entry = iterator.next()

except StopIteration :

entry = None

if entry is None :

break

batch.append(entry)

if batch :

yield batch

if Trinity == True:

outputfile1 = ( prefix + '_ContigDescrp_with_GC.txt')

outputfile2 = ( prefix + '_Contig_Coumpound_List.txt')

outputfile3 = ( prefix + '_ContigDescrp.txt')

with open(inputfile) as fasta_file:

identifier = []

length = []

description = []

gccontent = []

for title, sequence in SimpleFastaParser(fasta_file):

identifier.append(title.split(None, 1)[0])

length.append(len(sequence))

gccontent.append(GC(sequence))

description.append("No Description")

ContigDescrp = DataFrame(dict(Contigid = Series(identifier, name = 'Contigid'), ContigLength = Series(length, name = 'ContigLength'), GCContent = Series(gccontent, name = 'GCContent'), Description =Series(description, name = 'Description') )).set_index(['Contigid'])

ContigDescrp=ContigDescrp[["ContigLength", "Description", "GCContent"]]

ContigDescrp.to_csv(outputfile1, sep='\t', index=True)

ContigDescrp = ContigDescrp.drop('GCContent',1)

ContigDescrp.to_csv(outputfile3, sep='\t', index=True)

ContigDescrp['compoundid'] = [x.split("_i")[0] for x in ContigDescrp.index]

Contig_to_Compounds = ContigDescrp.compoundid

Contig_to_Compounds.to_csv("temp.txt", sep='\t', index=True)

os.system('awk -F"\t" \'{print $2 "\t" $1}\' temp.txt > '+ outputfile2)

os.system('rm temp.txt')

print "\nOUTPUT INFORMATION"

print "---------------------"

print ('Output description file (with GC content): ' + str(outputfile1))

print ('Output description file : ' + str(outputfile3))

print ('Output isoform/compound maping file : ' + str(outputfile2))

print('\nProcess took : ' + str(datetime.now()-startTime) + ' h:min:ss')

else:

outputfile = ( prefix + '_ParsedFasta.txt')

record_iter = SeqIO.parse(open(inputfile),"fasta")

print record_iter

for i, batch in enumerate(batch_iterator(record_iter, 500000)) :

filename = "group_%i.fasta" % (i+1)

handle = open(filename, "w")

count = SeqIO.write(batch, handle, "fasta")

itercount =+ i

handle.close()

print "Wrote %i records to %s" % (count, filename)

print "Total number of new fasta files : %i" % (itercount+1)

for i in range(1,itercount+2):

inputfile = './group_%i.fasta' % (i)

with open(inputfile) as fasta_file:

identifier = []

length = []

description = []

for title, sequence in SimpleFastaParser(fasta_file):

identifier.append(title.split(None, 1)[0])

length.append(len(sequence))

test = title.split(None, 1)[-1] == title.split(None, 1)[0]

#print test

if test is True:

description.append("No Description")

else:

description.append(title.split(None, 1)[1])

Parsedfasta = DataFrame(dict(subjectid = Series(identifier, name = 'subjectid'), subjectlength = Series(length, name = 'subjectlength'), subjectdescription =Series(description, name = 'subjectdescription') )).set_index(['subjectid'])

Parsedfasta=Parsedfasta[["subjectlength", "subjectdescription"]]

Parsedfasta.to_csv(('ParsedFasta_%i.txt' % i), sep='\t', index=True)

os.system('cat ParsedFasta_*.txt >'+ outputfile)

os.system('sed -i \'0,/ubjectid/! {/ubjectid/d}\' ' + outputfile)

os.system('rm ParsedFasta_*.txt group_*.fasta')

print "\nOUTPUT INFORMATION"

print "---------------------"

print ('Output file : ' + str(outputfile))

print( '\nProcess took : ' + str(datetime.now()-startTime) + ' h:min:ss')

# Bitscore2 evaluation

From a given blastx alignment sequence this script generates a bitscore2 value based on the maximum value the maximum bitscore an alignment can have.

The script uses only one argument : the extended tabular blast output file:

| Column | NCBI name | Description | |
| --- | --- | --- | --- |
| 1 | qseqid | Query Seq-id (ID of your sequence) | |
| 2 | sseqid | Subject Seq-id (ID of the database hit) | |
| 3 | pident | Percentage of identical matches | |
| 4 | length | Alignment length | |
| 5 | mismatch | Number of mismatches | |
| 6 | gapopen | Number of gap openings | |
| 7 | qstart | Start of alignment in query | |
| 8 | qend | End of alignment in query | |
| 9 | sstart | Start of alignment in subject (database hit) | |
| 10 | send | End of alignment in subject (database hit) | |
| 11 | evalue | Expectation value (E-value) | |
| 12 | bitscore | Bit score | |
| 13 | sallseqid | All subject Seq-id(s), separated by a ';' |  |
| 14 | score | Raw score |  |
| 15 | nident | Number of identical matches |  |
| 16 | positive | Number of positive-scoring matches |  |
| 17 | gaps | Total number of gaps |  |
| 18 | ppos | Percentage of positive-scoring matches |  |
| 19 | qframe | Query frame |  |
| 20 | sframe | Subject frame |  |
| 21 | qseq | Aligned part of query sequence |  |
| 22 | sseq | Aligned part of subject sequence |  |
| 23 | qlen | Query sequence length |  |
| 24 | slen | Subject sequence length |  |

The alignment sequence should be on the 22nd column, the query id on the 2nd

Output has 14 columns: |queryid|subjectid|identity|alignmentlength|mismatches|gapopens|qstart|qend|sstart|send|evalue|blastxbitscore|blastpbitscore|OptimalBitscore|

OUTPUT: prefix-of-file_optbitscore.txt

COMMAND LINE: bash $LINUX_SCRIPTS/bitscore2.sh /FullDirectory/blast_extended_output.tab

--- script (bash) ---

#!/bin/bash

set -e

#Initial check

if [ $# -lt 1 ]; then

echo "Not enough arguments provided (1 needed, you have $#)"

exit 1

fi

if [ $# -gt 1 ]; then

echo "too many arguments provided (1 needed, you have $#)"

exit 1

fi

FILE=$1

if [ -f $FILE ];

then

echo ""

else

echo "Can't find file $FILE"

fi

echo -e "\necho -e "Bash version ${BASH_VERSION}..."Start : $(date)\n"

echo -e ""

echo -e "This script extracts the query alignment sequence that had a hit in a previous blast."

echo -e "It then perform a blastp against itself to obtain an optimal bitscore the the alignment."

echo -e "The script uses only one argument : the extended tabular blast output file."

echo -e "The alignment sequence should be on the 22nd column, the query id on the 2nd."

echo -e "Output has 14 columns: |queryid|subjectid|identity|alignmentlength|mismatches|gapopens|qstart|qend|sstart|send|evalue|blastxbitscore|blastpbitscore|OptimalBitscore|"

echo -e "OUTPUT: prefix-of-file_optbitscore.txt"

echo -e "COMMAND LINE: bash $LINUX_SCRIPTS/bitscore2.sh /FullDirectory/blast_extended_output.tab\n\n\n"

mkdir blastp_bitscore_identity

cd blastp_bitscore_identity

filename=$(basename "$1" | cut -d. -f1)_optbitscore.txt

awk -F'\t' -vOFS='\t' '{ gsub("-", "", $21) ; print }' $1 | awk -F'\t' -vOFS='\t' '{ gsub("-", "", $22) ; print }' | cat -n > input_blast_extended.txt

sed -i 's/^[ ][ ]*//g' input_blast_extended.txt

cut -f1,2,22,23 input_blast_extended.txt | awk -F"\t" '{print $1 "\t" $2 "\t" $3 "\t" $4 "\t" $3$4 }'> querydb_seq_hit.txt

awk -F"\t" '!seen[$5]++' querydb_seq_hit.txt > uniq_querydb_seq_hit.txt

awk -F"\t" '{print $1 "\t" $3 "\n" $1 "\t" $4 }' uniq_querydb_seq_hit.txt > querydb_seq_hit.fasta

sed -i 's/^/>/g' querydb_seq_hit.fasta

sed -i 's/\t/\n/g' querydb_seq_hit.fasta

echo -e "\n-------------------------------"

echo -e "Starting translated query against protein hit\n"

grep -v '^$' querydb_seq_hit.fasta|\

while read A

do

echo "$A" > query.fa

read B

echo "$B" >> query.fa

read C

echo "$C" > prot.fa

read D

echo "$D" >> prot.fa

blastp -query query.fa -subject prot.fa -max_target_seqs 1 -word_size 3 -outfmt "6 qseqid sseqid bitscore" >> _blastp_bitscore_query.txt

done

awk -F"\t" '!seen[$1]++' _blastp_bitscore_query.txt > blastp_bitscore_query.txt

echo -e "Done"

echo -e "-------------------------------\n"

echo -e "\n-------------------------------"

echo -e "Starting blastp protein against itself\n"

cut -f1,3,23 input_blast_extended.txt > proteindb_seq_hit.txt

awk -F"\t" '!seen[$3]++' proteindb_seq_hit.txt > uniq_proteindb_seq_hit.txt

cut -f1,3 uniq_proteindb_seq_hit.txt > proteindb_seq_hit.fasta

sed -i 's/^/>/g' proteindb_seq_hit.fasta

sed -i 's/\t/\n/g' proteindb_seq_hit.fasta

grep -v '^$' proteindb_seq_hit.fasta|\

while read T

do

echo "$T" > prot.fa

read S

echo "$S" >> prot.fa

blastp -query prot.fa -subject prot.fa -max_target_seqs 1 -word_size 3 -outfmt "6 qseqid sseqid bitscore" >> _optimal_bitscore_prot.txt

done

awk -F"\t" '!seen[$1]++' _optimal_bitscore_prot.txt > optimal_bitscore_prot.txt

echo -e "Done"

echo -e "-------------------------------\n"

echo -e "\n-------------------------------"

echo -e "Gathering data\n"

join -1 1 -2 1 -t $'\t' -o 1.1 1.3 2.5 -e ERROR <(sort -t $'\t' -k1,1 blastp_bitscore_query.txt) <(sort -t $'\t' -k1,1 uniq_querydb_seq_hit.txt) > join1_uniq_query_optbit.txt

join -1 5 -2 3 -t $'\t' -o 1.1 2.2 -e ERROR <(sort -t $'\t' -k5,5 querydb_seq_hit.txt) <(sort -t $'\t' -k3,3 join1_uniq_query_optbit.txt) > join2_all_query_optbit.txt

join -1 1 -2 1 -t $'\t' -o 1.1 1.3 2.3 -e ERROR <(sort -t $'\t' -k1,1 optimal_bitscore_prot.txt) <(sort -t $'\t' -k1,1 uniq_proteindb_seq_hit.txt) > join1_uniq_protein_optbit.txt

join -1 3 -2 3 -t $'\t' -o 1.1 2.2 -e ERROR <(sort -t $'\t' -k3,3 proteindb_seq_hit.txt) <(sort -t $'\t' -k3,3 join1_uniq_protein_optbit.txt) > join2_all_protein_optbit.txt

join -1 1 -2 1 -t $'\t' -o 1.1 1.2 2.2 -e ERROR <(sort -t $'\t' -k1,1 join2_all_query_optbit.txt) <(sort -t $'\t' -k1,1 join2_all_protein_optbit.txt) > optimal_bitscore_query_prot.txt

join -1 1 -2 1 -t $'\t' <(sort -t $'\t' -k1,1 input_blast_extended.txt) <(sort -t $'\t' -k1,1 optimal_bitscore_query_prot.txt) | cut -f2-13,26,27 > final_notsorted.txt

sort -k 1,1 -k 12,12rg final_notsorted.txt > $filename

sed -i '1s/^/queryid\tsubjectid\tidentity\talignmentlength\tmismatches\tgapopens\tqstart\tqend\tsstart\tsend\tevalue\tblastxbitscore\tblastpbitscore\tOptimalBitscore\n/' $filename

cp $filename ../

rm query.fa prot.fa

echo -e "Done"

# Bitscore2 parser suite – 1

Parse blast output by extracting highest scores alignement based on optimal bitscore. Only hits with small change (<10) in optimal bitscore between 2 consecutive hits will be kept.

#!/usr/bin/python

import sys

import os

from datetime import datetime

import argparse

import pandas as pd

from pandas import *

import matplotlib

matplotlib.use('Agg')

import matplotlib.pyplot as plt

import re

print "Pandas version : " + pandas.__version__

startTime = datetime.now()

parser = argparse.ArgumentParser(description="Parse blast output by extracting highest scores alignement based on optimal bitscore. Only hits with small change (<10) in optimal bitscore between 2 consecutive hits will be kept. Check _data_detailed_output.txt for detailed data of that process.",\

epilog='Output: prefix_b2parsed.txt or prefix_queries_b2parsed.txt" \

The outputfile will have only one hit per query sequence.\n \

Output columns: |queryid|geneid|querylength|subjectid|subjectlength|identity|evalue|Alignment_vs_Q_SeqLength|Alignment_vs_S_SeqLength|subjectdescription|bitscore|OptimalBitscore|bitscore2|')

parser.add_argument('-i', '--blastoutfile', help='[REQUIRED] 14 column file that is produced by bitscore2.sh:|queryid|subjectid|identity|alignmentlength|mismatches|gapopens|qstart|qend|sstart|send|evalue|blastxbitscore|blastpbitscore|optbit| \n Headers labels are not expected. Remove the first line if you have headers.', dest='inputfile1', action='store', required=True)

parser.add_argument('-q', '--queryfile', help='[REQUIRED] Query parsed fasta file. You should run the script fastaparser.py on your query fasta file to get it. The file should contain 3 column with sequences id, length, and description. Headers are assumed, first row will be skipped. NOTE: If from Trinity, this file should be name (whatever_ContigDescrp.txt) and should have 4 columns. You should remove GC-content column (easy with cut command : cut -f-3 whatever_ContigDescrp.txt > whatever_ContigDescrp_nGCcolumn.txt).',dest='inputfile2', action='store')

parser.add_argument('-s', '--subjectfile', help='[REQUIRED] Subject (or database) parsed fasta file. You should run the script fastaparser.py on your subject fasta file to get it. The file should contain 3 column with sequences id, length, and description. Headers are assumed, first row will be skipped. ',dest='inputfile3', action='store', required=True)

parser.add_argument('-p', '--prefix', help='[REQUIRED] Prefix is a string that will start the default outputfile name. Example : -p Swissprot_vs_Trinity', dest='prefix', action='store', required=True)

parser.add_argument('-t', '--trinity', help='[OPTIONAL] Subject sequences are Trinity subject sequences (e.g comp1_c0_seq1). If -t is applied, two files will be generated: one for isoforms and one for genes.', dest='trinity', default=False, action='store_true')

parser.add_argument('-b', '--bypass', help='[OPTIONAL] Fix bitscore2 > 100 to 100. You have to check that it is just a small bitscore difference, otherwise it could be an error. Run the program once without bypass option, then if the sprogram stops because of bitscore > 100, check the values that are producing the error, and if it is just a small mistake, re-run the program with the option.', dest='bypass', default=False, action='store_true')

args = parser.parse_args()

inputfile1 = args.inputfile1

inputfile2 = args.inputfile2

inputfile3 = args.inputfile3

prefix = args.prefix

Trinity = args.trinity

bypass = args.bypass

data = pd.read_csv(inputfile1, sep='\t',header=None)

try:

len(data.columns) == 14

data.columns = ['queryid', 'subjectid', 'identity', 'alignmentlength', 'mismatches', 'gapopens', 'qstart', 'qend', 'sstart', 'send', 'evalue', 'blastxbitscore', 'blastpbitscore', 'OptimalBitscore']

except:

print "\n\n***************"

print "Error detected!"

print "Problem with :", inputfile1

print "\n\n"

print data.head()

print "\n\n"

print "Make sure that you have 14 columns from bitscore2.sh: qseqid sseqid pident length mismatch gapopen qstart qend sstart send evalue blastxbitscore blastpbitscore OptimalBitscore"

print "***************"

print "\n\n"

sys.exit(1)

if isinstance(data.ix[0, 'blastpbitscore'], float) == True:

pass

else:

if isinstance(data.ix[0, 'blastpbitscore'], int) == True:

pass

else:

print "\n\n***************"

print "Error detected!"

print "Problem with :", inputfile1

print "\n\n"

print data.head()

print "\n\n"

print "Be sure to have no headers"

print "If you need to remove headers, use:"

print "awk 'FNR>1' problematic_file > new_file"

print "***************"

print "\n\n"

sys.exit(1)

Qfasta = pd.read_csv(inputfile2, sep='\t',skiprows=1, header=None)

try:

len(Qfasta.columns) == 3

Qfasta.columns = ['queryid', 'querylength', 'querydescription']

Qfasta = Qfasta.set_index(['queryid'])

except:

print "\n\n***************"

print "Error detected!"

print "Problem with :", inputfile2

print "\n\n"

print Qfasta.head()

print "\n\n"

print "Make sure that you have 3 columns : sequences id, length, and description"

print "If you have a 4th column : GC content, you can remove it by doing :"

print "cut -f-3 old_file > new_file"

print "***************"

print "\n\n"

sys.exit(1)

try:

int(Qfasta.ix[0,'querylength'])

except:

print "\n\n***************"

print "Error detected!"

print "Problem with :", inputfile3

print "\n\n"

print Qfasta.head()

print "\n\n"

print "querylength column : you should have integers."

print "Make sure that you have 3 columns : sequences id, length, and description"

print "If you need to swap the 2nd and 3rd column, use:"

print "awk -F\"\\t\" '{ print $1 \"\\t\" $3 \"\\t\" $2}' problematic_file > new_file"

print "***************"

print "\n\n"

sys.exit(1)

if isinstance(Qfasta.ix[0, 'querydescription'], str) == True:

pass

else:

print "\n\n***************"

print "Error detected!"

print "Problem with :", inputfile3

print "\n\n"

print Qfasta.head()

print "\n\n"

print "querydescription column : you should have strings."

print "Make sure that you have 3 columns : sequences id, length, and description"

print "If you need to swap the 2nd and 3rd column, use:"

print "awk -F\"\\t\" '{ print $1 \"\\t\" $3 \"\\t\" $2}' problematic_file > new_file"

print "***************"

print "\n\n"

sys.exit(1)

Sfasta = pd.read_csv(inputfile3, sep='\t', skiprows=1, header=None)

try:

len(Sfasta.columns) == 3

Sfasta.columns = ['subjectid', 'subjectlength', 'subjectdescription']

Sfasta = Sfasta.set_index(['subjectid'])

except:

print "\n\n***************"

print "Error detected!"

print "Problem with :", inputfile3

print "\n\n"

print Sfasta.head()

print "\n\n"

print "Make sure that you have 3 columns : sequences id, length, and description"

print "If you have a 4th column : GC content, you can remove it by doing :"

print "cut -f-3 old_file > new_file"

print "***************"

print "\n\n"

sys.exit(1)

try:

int(Sfasta.ix[0,'subjectlength'])

except:

print "\n\n***************"

print "Error detected!"

print "Problem with :", inputfile3

print "\n\n"

print Sfasta.head()

print "\n\n"

print "subjectlength column : you should have integers."

print "Make sure that you have 3 columns : sequences id, length, and description"

print "If you need to swap the 2nd and 3rd column, use:"

print "awk -F\"\\t\" '{ print $1 \"\\t\" $3 \"\\t\" $2}' problematic_file > new_file"

print "***************"

print "\n\n"

sys.exit(1)

if isinstance(Sfasta.ix[0, 'subjectdescription'], str) == True:

pass

else:

print "\n\n***************"

print "Error detected!"

print "Problem with :", inputfile3

print "\n\n"

print Sfasta.head()

print "\n\n"

print "subjectdescription column : you should have strings."

print "Make sure that you have 3 columns : sequences id, length, and description"

print "If you need to swap the 2nd and 3rd column, use:"

print "awk -F\"\\t\" '{ print $1 \"\\t\" $3 \"\\t\" $2}' problematic_file > new_file"

print "***************"

print "\n\n"

sys.exit(1)

pattern="PACid"

db = re.search(pattern, str(data.ix[1, 'subjectid'])) == None

if db == False:

print "*************************\nNOTE: problem detected"

print "Problematic protein id :", data.ix[1, 'subjectid']

data['subjectid']=[x.split("|")[0] for x in data.subjectid] #Adding one column for accession number of database

print "Removing unwanted part of annotation."

print "Protein id updated:", data.ix[1, 'subjectid']

print "*************************\n"

print " INPUT SUMMARY "

print "-------------------------------"

print ('Blast inputfile in tabular format : ' + str(inputfile1))

print "Blast inputfile has", "{:,}".format(data.shape[0]), "records"

df_test=data.drop_duplicates('queryid')

print "Blast Inputfile has : ", "{:,}".format(df_test.shape[0]), " unique records"

print ('Query parsed fasta file : ' + str(inputfile2))

print "Query fasta file has", "{:,}".format(Qfasta.shape[0]), "records"

print ('Database parsed fasta file: ' + str(inputfile3))

print "Database fasta file has", "{:,}".format(Sfasta.shape[0]), "records"

print ('Prefix: ' + str(prefix))

print ('Data from trinity ? ' + str(Trinity))

print "-------------------------------\n"

print "Merging query descriptions with input file"

data = data.join(Qfasta, on='queryid', how='inner')#, lsuffix='_a', rsuffix='_b')

data['Q_AlignmentLength']=abs(data['qend']-data['qstart'])+1

data['Alignment_vs_Q_SeqLength']=(data['Q_AlignmentLength'].astype('float64')/data['querylength'].astype('float64'))*100

print "Done. {:,}".format(data.shape[0]), " records left."

df_test=data.drop_duplicates('queryid')

print "{:,}".format(df_test.shape[0]), " unique records left.\n"

print "Merging protein descriptions with input file"

data = data.join(Sfasta, on='subjectid', how='inner')

data['S_AlignmentLength']=abs(data['send']-data['sstart'])+1

data['Alignment_vs_S_SeqLength']=(data['S_AlignmentLength'].astype('float64')/data['subjectlength'].astype('float64'))*100

print "Done. {:,}".format(data.shape[0]), " records left."

df_test=data.drop_duplicates('queryid')

print "{:,}".format(df_test.shape[0]), " unique records left.\n"

#removing useless columns

data=data[['queryid', 'querylength', 'subjectid', 'subjectlength', "identity","evalue", "Alignment_vs_Q_SeqLength", 'S_AlignmentLength', "Alignment_vs_S_SeqLength", 'subjectdescription', "blastxbitscore", "blastpbitscore", "OptimalBitscore"]]

print "Evaluating bitscore filter."

data.loc[:,'Bitscore_identity']=data['blastpbitscore']/data['OptimalBitscore']*100

data.loc[:,'maxBitscore'] = data.groupby('queryid')['blastxbitscore'].transform(lambda x: x.max())

data.loc[:,'Confidence_coef']=data['blastxbitscore']/data['maxBitscore'].astype('float64')

data.loc[:,'bitscore2']=data['Bitscore_identity']*data['Confidence_coef']

maxbitscore_value=data.ix[data['bitscore2'].argmax(),'bitscore2']

print "Maximum bitscore2 value :", maxbitscore_value

if maxbitscore_value > 105.0:

if bypass == True:

data.loc[data.bitscore2 > 100.0, 'bitscore2'] = 100

else:

print "Error on bitscore2: at least one value is higher than 100. Check your protocol"

sys.exit()

data = data.drop(['S_AlignmentLength'],1)

f = lambda x: x.sort_values(by=['bitscore2', 'blastpbitscore', 'identity'], ascending=[False, False, False])

data = data.groupby('queryid').apply(f)

data=data.reset_index(0, drop=True)

#Add group weight column.

data['subindex'] = data.groupby(['queryid']).cumcount() + 1

data['GroupWeight']= data.groupby(['queryid'])['queryid'].transform('count')

#Here we see how values change. We want to separate groups of data that slowly change between them

#I'll subtract values with the one below

data['change'] = np.abs(np.round(data['bitscore2'] - data['bitscore2'].shift(+1)))

#the first row gives a NaN as there is no value above

data.fillna(0)

#Change border values to change2

data.ix[data.subindex == 1,['change']]= 0

print "Done. {:,}".format(data.shape[0]), " records left."

df_test=data.drop_duplicates('queryid')

print "{:,}".format(df_test.shape[0]), " unique records left.\n"

print "Applying bitscore filter."

#Identify rows where the change is equal or higher than 10 (bitscore2),

#and write in 'changelimit' column the number of the minimum subindex of the group

data['changelimit']=data[data.change >= 10].groupby('queryid')['subindex'].transform(lambda x: x.min())

data=data.fillna(0)

#Create a frame with the first row of each 'id' that's greater or equal to 10

rowindex = data['change'] >= 10

greaterThan10 = data[rowindex].groupby('queryid',as_index=False).first()

#Merge() of the original frame and greaterThan10

dfKeep = pd.merge(data,greaterThan10,how='left',on=['queryid'],suffixes=['','_cut'])

#We fix exagerated high values to NaN values. NaN values correspond, at this stage, to values that don't have a cut, which means values that we should be all kept.

dfKeep=dfKeep.fillna(100000000)

#Then filtering the new frame for the 'keepers'

mask = (dfKeep['subindex'] < dfKeep['subindex_cut'])

mask2 = (dfKeep['subindex'] >= dfKeep['subindex_cut'])

datalost2=dfKeep[mask2][['queryid', 'querylength', 'subjectid', 'subjectlength', 'identity', 'evalue', 'Alignment_vs_Q_SeqLength', 'Alignment_vs_S_SeqLength', 'subjectdescription', 'blastxbitscore', 'blastpbitscore', 'OptimalBitscore', 'Bitscore_identity', 'bitscore2','maxBitscore', 'subindex', 'GroupWeight', 'change','Confidence_coef']]

data=dfKeep[mask][['queryid', 'querylength', 'subjectid', 'subjectlength', 'identity', 'evalue', 'Alignment_vs_Q_SeqLength', 'Alignment_vs_S_SeqLength', 'subjectdescription', 'blastxbitscore', 'blastpbitscore', 'OptimalBitscore', 'Bitscore_identity', 'bitscore2','maxBitscore', 'subindex', 'GroupWeight', 'change','Confidence_coef']]

print "Done. {:,}".format(data.shape[0]), " records left."

df_test=data.drop_duplicates('queryid')

print "{:,}".format(df_test.shape[0]), " unique records left.\n"

print "{:,}".format(datalost2.shape[0]), " records removed. \n"

#Identify the range of bitscore that is conserved through the previous operation

data['diffbitscore']=0

data['diffbitscore']=data.groupby('queryid')['blastpbitscore'].transform(lambda x: x.max()-x.min())

data['maxbitscore']=data.groupby('queryid')['blastpbitscore'].transform(lambda x: x.max())

data['diffbitscorepercent']=data['diffbitscore']/data['maxbitscore']*100

data.to_csv('_conserveddata.txt', sep='\t', index=False)

####datalost1.to_csv('_discarded_rows1.txt', sep='\t', index=False)

datalost2.to_csv('_discarded_rows2.txt', sep='\t', index=False)

if Trinity == True:

data['geneid'] = [x.split("_i")[0] for x in data['queryid']]

data = data[['queryid', 'geneid', 'querylength', 'subjectid', 'subjectlength', "identity","evalue", "Alignment_vs_Q_SeqLength", "Alignment_vs_S_SeqLength", 'subjectdescription', "blastxbitscore", 'blastpbitscore', 'OptimalBitscore', "bitscore2"]]

data = data.rename(columns = {'blastxbitscore':'bitscore'})

outputfile = prefix + '_b2parsed.txt'

data.to_csv(outputfile, sep='\t', index=False)

#reset index

print "\n OUTPUT INFORMATION: "

print "-------------------------------"

print ('Output file name : ' + str(outputfile))

print "Output file has : ", "{:,}".format(data.shape[0]), " records"

df_test=data.drop_duplicates('queryid')

print "Output has : ", "{:,}".format(df_test.shape[0]), " unique records"

print( '\nWhole process took : ' + str(datetime.now()-startTime) + ' h:min:ss')

else:

data=data[['queryid', 'querylength', 'subjectid', 'subjectlength', "identity", "evalue", "Alignment_vs_Q_SeqLength", "Alignment_vs_S_SeqLength", 'subjectdescription', "blastxbitscore", 'blastpbitscore', 'OptimalBitscore', "bitscore2"]]

data = data.rename(columns = {'blastxbitscore':'bitscore'})

outputfile = prefix + '_queries_b2parsed.txt'

data.to_csv(outputfile, sep='\t', index=False)

print "\n OUTPUT INFORMATION: "

print "-------------------------------"

print ('Output file name : ' + str(outputfile))

print "Output file has : ", "{:,}".format(data.shape[0]), " records"

df_test=data.drop_duplicates('queryid')

print "Output has : ", "{:,}".format(df_test.shape[0]), " unique records"

print( '\nWhole process took : ' + str(datetime.now()-startTime) + ' h:min:ss')

# Bitscore2 parser suite – 2

Uses as inpufile concatenated files that has already been processed by bitscoreparser_part1.py. It's useful when we gather data and want to extract best blast hits from same sequences, but from different db. Based on the calculated proportion, the program will also extract the best hit for each query sequence by comparing all blast hits, just like bitscoreparser_part1.py. The outputfile will have only one hit per query sequence.

#!/usr/bin/python

import numpy as np

from datetime import datetime

import argparse

import pandas as pd

from pandas import *

import matplotlib

matplotlib.use('Agg')

import matplotlib.pyplot as plt

import locale

print "Pandas version : " + pandas.__version__

startTime = datetime.now()

parser = argparse.ArgumentParser(description="Uses as inpufile concatenated files that has already been processed by bitscoreparser_part1.py. It's useful when we gather data and want to extract best blast hits from same sequences, but from different db.",\

epilog='Based on the calculated proportion, the program will also extract the best hit for each query sequence by comparing all blast hits, just like bitscoreparser_part1.py. The outputfile will have only one hit per query sequence.\

Outputfile: prefix_b2parsed2.txt')

parser.add_argument('-i', '--blastoutfile', help='[REQUIRED] Concatenated output files from bitscoreparser_part1.py. This script expects 15 columns : queryid|geneid|querylength|subjectid|subjectlength|identity|evalue|Alignment_vs_Q_SeqLength|Alignment_vs_S_Seq|Length|subjectdescription|bitscore|blastpbitscore|OptimalBitscore|bitscore2. Headers are assumed, first row will be skipped. Input file example : -b File_vs_poplar_b2parsed.txt', dest='inputfile1', action='store', required=True)

parser.add_argument('-p', '--prefix', help='[REQUIRED] Prefix is a string that will start the default outputfile name. Example : -p Swissprot_vs_Trinity', dest='prefix', action='store', required=True)

parser.add_argument('-t', '--trinity', help='[OPTIONAL] Subject sequences are Trinity subject sequences (e.g comp1_c0_seq1). If -t is applied, two files will be generated: one for isoforms and one for genes.', dest='trinity', default=False, action='store_true')

args = parser.parse_args()

inputfile1 = args.inputfile1

prefix = args.prefix

Trinity = args.trinity

data = pd.read_csv(inputfile1, sep='\t')

print "\n INPUT SUMMARY "

print "-------------------------------"

print ('Blast inputfile in tabular format : ' + str(inputfile1))

print "Blast inputfile has", "{:,}".format(data.shape[0]), "records"

df_test=data.drop_duplicates('queryid')

print "Inputfile has : ", "{:,}".format(df_test.shape[0]), " unique records"

print ('Prefix: ' + str(prefix))

print ('Data from trinity ? ' + str(Trinity))

print "-------------------------------\n"

print "\n-----------------------------------------"

print "Re-evaluation of bitscore2, now that we mix databases together"

data = data.drop('bitscore2',1)

data.loc[:,'Bitscore_identity']=data['blastpbitscore']/data['OptimalBitscore']*100

#I'm correcting some values that are higher than 100 (because of the difference between blastp and blastx scoring that can happen sometimes). If the value is well above 100, it is a mistake that would have been detected already in the bitscore 2 process.

data.loc[data.Bitscore_identity > 100, 'Bitscore_identity'] = 100

#We'll correct the flaws by adding a weight value:

#sequences with low bitscores compared with the highest bitscore will have penalties

data.loc[:,'maxBitscore'] = data.groupby('queryid')['bitscore'].transform(lambda x: x.max())

data.loc[:,'Confidence_coef']=data['bitscore']/data['maxBitscore'].astype('float64')

data.loc[:,'bitscore2']=data['Bitscore_identity']*data['Confidence_coef']

data = data.drop(['maxBitscore', 'blastpbitscore', 'Confidence_coef','Bitscore_identity'],1)

f = lambda x: x.sort_values(by=['bitscore2', 'bitscore', 'identity'], ascending=[False, False, False])

data = data.groupby('queryid').apply(f)

#data.to_csv("_test_line_62.txt", sep='\t', index=False)

print "Done! data have", "{:,}".format(data.shape[0]), "records."

print "-----------------------------------------\n"

print "\n-----------------------------------------"

print "Filter: bitscore2 coverage"

f = lambda x: x.sort_values(by=['bitscore2', 'bitscore', 'identity'], ascending=[False, False, False])

data = data.groupby('queryid').apply(f)

data['max'] = data.groupby('queryid')['bitscore2'].transform(lambda x: x.max())

#Keep rows that have a bitscore2 value identical to ('max' -10)

data = data[data['bitscore2'] >= (data['max']-10)]

data = data.drop('max',1)

data=data.reset_index(0, drop=True)

print "Done! data have", "{:,}".format(data.shape[0]), "records left"

print "-----------------------------------------\n"

if Trinity == True:

data['geneid'] = [x.split("_seq")[0] for x in data['queryid']]

data=data[['queryid', 'geneid', 'querylength', 'subjectid', 'subjectlength', "identity","evalue", "Alignment_vs_Q_SeqLength", "Alignment_vs_S_SeqLength", 'subjectdescription', "bitscore", "bitscore2"]]

outputfile = prefix + '_b2parsed2.txt'

f = lambda x: x.sort_values(by=['bitscore2', 'bitscore', 'identity'], ascending=[False, False, False])

data = data.groupby('queryid').apply(f)

data.to_csv(outputfile, sep='\t', index=False)

print "\n OUTPUT INFORMATION: "

print "-------------------------------"

print ('Output file name : ' + str(outputfile))

print "Output file has : ", "{:,}".format(data.shape[0]), " records"

df_test=data.drop_duplicates('queryid')

print "Output file has : ", "{:,}".format(df_test.shape[0]), " unique records"

print( '\nWhole process took : ' + str(datetime.now()-startTime) + ' h:min:ss')

else:

data=data[['queryid', 'querylength', 'subjectid', 'subjectlength', "identity", "evalue", "Alignment_vs_Q_SeqLength", "Alignment_vs_S_SeqLength", 'subjectdescription', "bitscore", "bitscore2"]]

outputfile = prefix + '_queries_b2parsed2.txt'

data.to_csv(outputfile, sep='\t', index=False)

print "\n OUTPUT INFORMATION: "

print "-------------------------------"

print ('Output file name : ' + str(outputfile))

print "Output file has : ", "{:,}".format(data.shape[0]), " records"

df_test=data.drop_duplicates('queryid')

print "Output file has : ", "{:,}".format(df_test.shape[0]), " unique records"

print( '\nWhole process took : ' + str(datetime.now()-startTime) + ' h:min:ss')

# Bitscore2 parser suite – 3

Uses as inpufile a file that has already been processed by bitscoreparser_part1.py or bitscoreparser_part2.py. You can filter the results with an evalue cutoff, Alignment vs sequence length cutoff , or even forbidden words. This script extracts taxonomy information for each contig based on the associated database annotation. It also outputs pie charts based on total taxonomy.

#!/usr/bin/python

import numpy as np

from scipy import randn

from datetime import datetime

import argparse

import pandas as pd

from pandas import *

import matplotlib

matplotlib.use('Agg')

import matplotlib.pyplot as plt

import locale

import re

import sys

from scipy import stats

from scipy.stats import gaussian_kde

from pandas.tools.plotting import table

import os

import matplotlib.gridspec as gridspec

import matplotlib.cm as cm, matplotlib.font_manager as fm

def myround(x, base):

return int(base * round(float(x)/base))

#remove similar words in a string

def unique_list(l):

ulist = []

[ulist.append(x) for x in l if x not in ulist]

return ulist

#create function to round percentages to base (1, or 5, or 10, or whatever)

def myround(x, base):

return int(base * round(float(x)/base))

def percentile(n):

def percentile_(x):

return np.percentile(x, n)

percentile_.__name__ = 'N%s' % n

return percentile_

def counter(seq):

"""make a freq dict with species as key"""

seq_dict = {}

for n in seq:

if n in seq_dict:

seq_dict[n] += 1

else:

seq_dict[n] = 1

return seq_dict

title_font = fm.FontProperties(family='Bitstream Vera Sans', style='normal', size=15, weight='normal', stretch='normal')

label_font = fm.FontProperties(family='Bitstream Vera Sans', style='normal', size=12, weight='normal', stretch='normal')

ticks_font = fm.FontProperties(family='Bitstream Vera Sans', style='normal', size=10, weight='normal', stretch='normal')

annotation_font = fm.FontProperties(family='Bitstream Vera Sans', style='normal', size=10, weight='normal', stretch='normal')

def gbplot_pie(fractions, #values for the wedges

labels, #labels for the wedges

title = '', #title of the pie chart

savename = './pie_chart.pdf', #name and path when saving the chart

cm_name = 'Pastel1', #name of the matplotlib colormap to use

autopct = '%1.1f%%', #format the value text on each pie wedge

labeldistance = 1.05, #where to place wedge labels in relation to pie wedges

shadow = True, #shadow around the pie

startangle = 90, #rotate 90 degrees to start the top of the data set on the top of the pie

edgecolor = 'w', #color of pie wedge edges

width = 10, #width of the figure in inches

height = 10, #height of the figure in inches

grouping_threshold = None, #group all wedges below this value into one 'all others' wedge

grouping_label = None): #what the label the grouped wedge

if not grouping_threshold==None:

if grouping_label == None:

grouping_label = 'Others'

row_mask = fractions > grouping_threshold

meets_threshold = fractions[row_mask]

all_others = pd.Series(fractions[~row_mask].sum())

all_others.index = [grouping_label]

fractions = meets_threshold.append(all_others)

labels = fractions.index

color_map = cm.get_cmap(cm_name)

num_of_colors = len(fractions)

colors = color_map([x/float(num_of_colors) for x in range(num_of_colors)])

fig, ax = plt.subplots(figsize=[width, height])

small = fractions[len(fractions) / 2:]

angle = 180 + small.astype(float).cumsum()[-1] / fractions.astype(float).cumsum()[-1] * 360

wedges = ax.pie(fractions,

labels = labels,

labeldistance = labeldistance,

autopct = autopct,

colors = colors,

shadow = shadow,

startangle = angle)

for wedge in wedges[0]:

wedge.set_edgecolor(edgecolor)

ax.set_title(title, fontproperties=title_font)

fig.savefig(savename, dpi=350, bbox_inches='tight')

plt.close(fig)

startTime = datetime.now()

parser = argparse.ArgumentParser(description="Uses as inpufile (inputfile_b2parsed2.txt) a file that has already been processed by bitscoreparser_part1.py or bitscoreparser_part2.py. You can filter the results with an evalue cutoff, Alignment vs sequence length cutoff , or even forbidden words.", epilog = "The output file will look like : inputfile_b2parsed3.txt")

parser.add_argument('-i', '--blastoutfile', help='[REQUIRED] File from bitscoreparser_part2.py. This script expects 20 columns : queryid geneid subjectid identity alignmentlength mismatches gapopens qstart qend sstart send evalue bitscore querylength Q_AlignmentLength Alignment_vs_Q_SeqLength subjectdescription subjectlength S_AlignmentLength Alignment_vs_S_SeqLength. Headers are assumed, first row will be skipped. Input file example : -b filename_isoforms_alignpercent.txt', dest='inputfile1', action='store', required=True)

parser.add_argument('-nr', '--nr', help='[OPTIONAL] nr database file location. It should have the following headers: ID|OC|GO|KEGG|KO|Pfam|PANTHER. For example: -nr /storage/db/nr/nr_Sigmund_parsed.dat', dest='nr', default = "", action='store', type=str)

parser.add_argument('-tr', '--trembl', help='[OPTIONAL] Trembl database file location. It should have the following headers: ID|AC|OC|GO|KEGG|KO|Pfam|PANTHER. For example: -tr /storage/db/trembl/trembl_Sigmund_parsed.dat', dest='tr', default = "", action='store', type=str)

parser.add_argument('-sp', '--swissprot', help='[OPTIONAL] swissprot database file location. It should have the following headers: ID|AC|OC|GO|KEGG|KO|Pfam|PANTHER. For example: -tr /storage/db/swissprot/swissprot_Sigmund_parsed.dat', dest='sp', default = "", action='store', type=str)

parser.add_argument('-uniparc', '--uniparc', help='[OPTIONAL] uniparc database file location. It should have the following headers: ID|AC|OC|GO|KEGG|KO|Pfam|PANTHER. For example: -tr /storage/db/uniparc/uniparc_Sigmund_parsed.dat', dest='uniparc', default = "", action='store', type=str)

parser.add_argument('-salix', '--salixpurpurea', help='[OPTIONAL] salix database file location. It should have the following headers: GeneID|transcriptID|proteinID|ProteinLength|description|PFAM|KOG|KEGGec|KEGOrthology|GO|Panther. For example: -salix /storage/db/salix/Spurpurea_Annotation.txt', dest='salix', default = "", action='store', type=str)

parser.add_argument('-poplar', '--populus', help='[OPTIONAL] poplar database file location. It should have the following headers: GeneID|transcriptID|proteinID|ProteinLength|description|PFAM|KOG|KEGGec|KEGOrthology|GO|Panther. For example: -poplar /storage/db/poplar/Ptrichocarpa_Annotation.txt', dest='poplar', default = "", action='store', type=str)

parser.add_argument('-nt', '--nt', help='[OPTIONAL] nt database file location. It should have the following headers: ID|AC|OC. For example: -nt /storage/db/nt/nt_parsed.dat', dest='pyronema', default = "", action='store', type=str)

parser.add_argument('-e', '--evalue', help='[OPTIONAL] Filters input blast file by evalue. Removes any record that has a evalue higher than the filter. Example : -e 1e-7 (or 0.0000001).', dest='evalue', default = 10, action='store', type=float)

parser.add_argument('-q', '--qcutoff', help='[OPTIONAL] Filters inputfile Alignment_vs_Q_SeqLength value. Removes any record that has a percentage lower than the filter. Example : -q 60).', dest='qcutoff', default = 0, action='store', type=float)

parser.add_argument('-s', '--scutoff', help='[OPTIONAL] Filters inputfile Alignment_vs_S_SeqLength value. Removes any record that has a percentage lower than the filter. Example : -s 60).', dest='scutoff', default = 0, action='store', type=float)

parser.add_argument('-id', '--identity', help='[OPTIONAL] Filters inputfile identity blast value. Removes any record that has a percentage lower than the filter. Example : -id 60).', dest='identity', default = 101, action='store', type=float)

parser.add_argument('-qw', '--qwords', help='[OPTIONAL] Filters inputfile queryid. Removes any record that has common word with filter. Example : -qw "MOUSE mouse mus sapiens SAPIENS").', dest='qwords', default = '', action='store', type=str)

parser.add_argument('-sw', '--swords', help='[OPTIONAL] Filters inputfile subjectid and subjectdescription fields. Removes any record that has common word with filter. Example : -sw "MOUSE mouse mus sapiens Sapiens SAPIENS").', dest='swords', default = '', action='store', type=str)

parser.add_argument('-p', '--prefix', help='[OPTIONAL] Prefix is a string that will start the default outputfile name. Example : -p Swissprot_vs_Trinity', dest='prefix', action='store', default = '', type=str)

args = parser.parse_args()

inputfile1 = args.inputfile1

outputfile = inputfile1.split('.txt')[0] + '_b2parsed_parsed.txt'

nr = args.nr

tr = args.tr

sp = args.sp

uniparc = args.uniparc

salix = args.salix

poplar = args.poplar

tetur = args.tetur

tuber = args.tuber

laccaria = args.laccaria

ecoli = args.ecoli

pyronema = args.pyronema

if (nr != "") or (tr != "") or (sp != "") or (uniparc != "") or (salix != "") or (poplar != "") or (tetur != "") or (tuber != "") or (laccaria != "") or (ecoli != "") or (pyronema != ""):

annotation = "yes"

else:

annotation =""

prefix = args.prefix

evalue = args.evalue

qcutoff = args.qcutoff

scutoff = args.scutoff

qwords = args.qwords

swords = args.swords

identity = args.identity

data = pd.read_csv(inputfile1, sep='\t')

print '\n'

print " INPUT SUMMARY "

print "-------------------------------"

print ('Inputfile : ' + str(inputfile1))

print "Inputfile has : ", "{:,}".format(data.shape[0]), " records"

df_test=data.drop_duplicates('queryid')

print "Inputfile has : ", "{:,}".format(df_test.shape[0]), " unique records"

print "Highest evalue : ", data.ix[data['evalue'].argmax(),'evalue']

print "Mean evalue value : ", "{:.0e}".format(data['evalue'].mean())

print "Mean bitscore2 :", data['bitscore2'].mean().astype(int), "%"

print "Lowest bitscore2 :", data.ix[data['bitscore2'].argmin(),'bitscore2'].astype(int), "%"

print "Mean blast alignement identity :", data['identity'].mean().astype(int), "%"

print "Lowest blast alignement identity :", data.ix[data['identity'].argmin(),'identity'].astype(int), "%"

print "Lowest subject sequence vs alignment percentage:", data.ix[data['Alignment_vs_S_SeqLength'].argmin(),'Alignment_vs_S_SeqLength'].astype(int), "%"

print "Maximum subject sequence vs alignment percentage:", data.ix[data['Alignment_vs_S_SeqLength'].argmax(),'Alignment_vs_S_SeqLength'].astype(int), "%"

print "Mean subject sequence vs alignment percentage :", data['Alignment_vs_S_SeqLength'].mean().astype(int), "%"

print "-------------------------------"

if annotation == "":

print "\nAnnotation? : no"

else:

print "\nAnnotation? : yes"

if identity == 101:

pass

else:

data = data[data['identity']>= identity] #Keep rows that have an identity higher or identical to 'identity' value

data = data.reset_index(drop=True) #reset index

if qcutoff == 0:

pass

else:

print ('Query sequence vs alignment percentage filter : '), qcutoff

if scutoff == 0:

pass

else:

print ('Subject sequence vs alignment percentage filter : '), scutoff

if qwords == "":

pass

else:

print ('Query description filter(s) : '), qwords

if swords == "":

pass

else:

print ('Subject description filter(s) : '), swords

if qcutoff == 0:

pass

else:

data = data[data['Alignment_vs_Q_SeqLength']>=qcutoff] #Keep rows that have query sequence vs alignment percentage higher or identical to 'qcutoff' value

data = data.reset_index(drop=True) #reset index

if scutoff == 0:

pass

else:

data = data[data['Alignment_vs_S_SeqLength']>=scutoff] #Keep rows that have query sequence vs alignment percentage higher or identical to 'qcutoff' value

data = data.reset_index(drop=True) #reset index

if qwords == "":

pass

else:

qlwords = qwords.split()

pattern = '|'.join(qlwords)

#print pattern

data['qwords'] = data.queryid.str.contains(pattern)

#

data = data[data['qwords']==False]

data = data.drop('qwords',1)

data = data.reset_index(drop=True)

if swords == "":

pass

else:

slwords = swords.split()

pattern = '|'.join(slwords)

data['swords'] = data.subjectid.str.contains(pattern)

#

data = data[data['swords']==False]

data = data.drop('swords',1)

data = data.reset_index(drop=True)

data['swords'] = data.subjectdescription.str.contains(pattern)

#

data = data[data['swords']==False]

data = data.drop('swords',1)

data = data.reset_index(drop=True)

print "\n PARSED FILE SUMMARY: "

print "-------------------------------"

#print ('Parsed blast file name : ' + str(outputfile))

#print "Parsed blast file has : ", "{:,}".format(data.shape[0]), " records"

if data.shape[0] >= 1:

print "Now, inputfile has : ", "{:,}".format(data.shape[0]), " records"

df_test=data.drop_duplicates('queryid')

print "Inputfile has : ", "{:,}".format(df_test.shape[0]), " unique records"

print "Mean blast alignement identity :", data['identity'].mean().astype(int), "%"

print "Lowest blast alignement identity :", data.ix[data['identity'].argmin(),'identity'].astype(int), "%"

print "Lowest subject sequence vs alignment percentage:", data.ix[data['Alignment_vs_S_SeqLength'].argmin(),'Alignment_vs_S_SeqLength'].astype(int), "%"

print "Maximum subject sequence vs alignment percentage:", data.ix[data['Alignment_vs_S_SeqLength'].argmax(),'Alignment_vs_S_SeqLength'].astype(int), "%"

print "Mean subject sequence vs alignment percentage :", data['Alignment_vs_S_SeqLength'].mean().astype(int), "%"

else:

print "No more data in file. You should change the parameters"

sys.exit(1)

#************************************************************************************************************

if annotation == "":

pass

else:

#change outputfile name

outputfile = inputfile1.split('_b2parsed2.txt')[0] + '_b2parsed3.txt'

data['subjectid'].replace(to_replace="Potri", value="Potri|", inplace=True,regex=True)

data['subjectid'].replace(to_replace="SapurV", value="Sapur|V", inplace=True,regex=True)

data.loc[:,'DataBase']=[x.split("|")[0] for x in data['subjectid'].astype(str)]

data['subjectid'].replace(to_replace="Potri\|", value="Potri", inplace=True, regex=True)

data['subjectid'].replace(to_replace="Sapur\|", value="Sapur", inplace=True, regex=True)

data = data.reset_index(drop=True)

grouped = data.groupby('DataBase')

data2 = pd.DataFrame(columns=['queryid', 'geneid', 'identity', 'evalue', 'bitscore', 'bitscore2', 'querylength', 'Alignment_vs_Q_SeqLength', 'subjectid', 'ID', 'subjectlength', 'Alignment_vs_S_SeqLength', 'subjectdescription', 'GO', 'PFAM', 'Panther', 'KEGG', 'KEGGec', 'KO', 'KOGAnnotation', 'Taxonomy'])

nr_size = 0

tr_size = 0

sp_size = 0

uniparc_size = 0

sapur_size = 0

Potri_size = 0

for name, group in grouped:

if name == "gi":

print "\n\n------------------------"

print "Starting", name, "analysis."

datanr=grouped.get_group("gi")

datanr.columns = data.columns

datanr.loc[:,'ID']=[x.split("|")[1] for x in datanr['subjectid']]

if nr == "" :

print "\nYou should add nr to the input databases files."

print "Check if the file exists"

sys.exit(1)

inputnr=read_csv(nr,sep='\t', dtype={0:str, 1:str, 2:str, 3:str, 4:str, 5:str, 6:str, 7:str})

print "Database file is: ", nr

datanr = merge(datanr, inputnr, on='ID')

inputnr=[]

datanr = datanr.drop(['DataBase'],1)

datanr=datanr.rename(columns = {'OC':'Taxonomy'})

datanr.loc[:,'KEGGec']="0"

datanr.loc[:,'KOGAnnotation']="0"

datanr=datanr.rename(columns = {'Pfam':'PFAM'})

datanr=datanr.rename(columns = {'PANTHER':'Panther'})

datanr=datanr[['queryid', 'geneid', 'identity', 'evalue', 'bitscore', 'bitscore2', 'querylength', 'Alignment_vs_Q_SeqLength', 'subjectid', 'ID', 'subjectlength', 'Alignment_vs_S_SeqLength', 'subjectdescription', 'GO', 'PFAM', 'Panther', 'KEGG', 'KEGGec', 'KO', 'KOGAnnotation', 'Taxonomy']]

nr_size = len(datanr)

data2= datanr.append(data2).reset_index(drop=True)

#print data2

print "Done!"

print "------------------------\n\n"

if name == "tr":

print "\n\n------------------------"

print "Starting", name, "analysis."

datatr=grouped.get_group("tr")

datatr.columns = data.columns

datatr.loc[:,'AC']=[x.split("|")[2] for x in datatr['subjectid']]

if tr == "" :

print "\nYou should add trembl to the input databases files."

print "Check if the file exists"

sys.exit(1)

inputtr = pd.read_csv(tr, sep='\t', dtype={0:str, 1:str, 2:str, 3:str, 4:str, 5:str, 6:str, 7:str, 8:str})

print "Database file is: ", tr

datatr = merge(datatr, inputtr, on='AC')

inputtr=[]

datatr = datatr.drop(['DataBase', 'AC'],1)

datatr=datatr.rename(columns = {'OC':'Taxonomy'})

datatr.loc[:,'KEGGec']="0"

datatr.loc[:,'KOGAnnotation']="0"

datatr=datatr.rename(columns = {'Pfam':'PFAM'})

datatr=datatr.rename(columns = {'PANTHER':'Panther'})

datatr=datatr[['queryid', 'geneid', 'identity', 'evalue', 'bitscore', 'bitscore2', 'querylength', 'Alignment_vs_Q_SeqLength', 'subjectid', 'ID', 'subjectlength', 'Alignment_vs_S_SeqLength', 'subjectdescription', 'GO', 'PFAM', 'Panther', 'KEGG', 'KEGGec', 'KO', 'KOGAnnotation', 'Taxonomy']]

tr_size = len(datatr)

data2= datatr.append(data2).reset_index(drop=True)

print "Done!"

print "------------------------\n\n"

if name == "sp":

print "\n\n------------------------"

print "Starting", name, "analysis."

datasp=grouped.get_group("sp")

datasp.columns = data.columns

datasp.loc[:,'AC']=[x.split("|")[2] for x in datasp['subjectid']] #ex: extract CH10C_ARATH for sp|O65282|CH10C_ARATH

if sp == "" :

print "\nYou should add swissprot to the input databases files."

print "Check if the file exists"

sys.exit(1)

inputsp = pd.read_csv(sp, sep='\t')

print "Database file is: ", sp

datasp = merge(datasp, inputsp, on='AC')

inputsp=[]

#datasp.to_csv("_datasP_465.txt", sep='\t', index=False, header=True)

datasp = datasp.drop(['DataBase', 'AC'],1)

datasp=datasp.rename(columns = {'OC':'Taxonomy'})

#print datasp.head(n=2)

datasp.loc[:,'KEGGec']="0"

datasp.loc[:,'KOGAnnotation']="0"

datasp=datasp.rename(columns = {'Pfam':'PFAM'})

datasp=datasp.rename(columns = {'PANTHER':'Panther'})

datasp=datasp[['queryid', 'geneid', 'identity', 'evalue', 'bitscore', 'bitscore2', 'querylength', 'Alignment_vs_Q_SeqLength', 'subjectid', 'ID', 'subjectlength', 'Alignment_vs_S_SeqLength', 'subjectdescription', 'GO', 'PFAM', 'Panther', 'KEGG', 'KEGGec', 'KO', 'KOGAnnotation', 'Taxonomy']]

sp_size = len(datasp)

data2= datasp.append(data2).reset_index(drop=True)

print "Done!"

print "------------------------\n\n"

if name == "uniparc":

print "\n\n------------------------"

print "Starting", name, "analysis."

datauniparc=grouped.get_group("uniparc")

datauniparc.columns = data.columns

datauniparc.loc[:,'ID']=[x.split("|")[-1] for x in datauniparc['subjectid']]

#check if the file is in the provided folder

if uniparc == "" :

print "\nYou should add uniparc to the input databases files."

print "Check if the file exists"

sys.exit(1)

inputuniparc = pd.read_csv(uniparc, sep='\t')

print "Database file is: ", uniparc

datauniparc = merge(datauniparc, inputuniparc, on='ID')

inputuniparc=[]

datauniparc = datauniparc.drop(['DataBase', 'AC'],1)

datauniparc=datauniparc.rename(columns = {'OC':'Taxonomy'})

datauniparc.loc[:,'KEGGec']="0"

datauniparc.loc[:,'KOGAnnotation']="0"

datauniparc=datauniparc.rename(columns = {'Pfam':'PFAM'})

datauniparc=datauniparc.rename(columns = {'PANTHER':'Panther'})

datauniparc=datauniparc[['queryid', 'geneid', 'identity', 'evalue', 'bitscore', 'bitscore2', 'querylength', 'Alignment_vs_Q_SeqLength', 'subjectid', 'ID', 'subjectlength', 'Alignment_vs_S_SeqLength', 'subjectdescription', 'GO', 'PFAM', 'Panther', 'KEGG', 'KEGGec', 'KO', 'KOGAnnotation', 'Taxonomy']]

uniparc_size = len(datauniparc)

data2= datauniparc.append(data2).reset_index(drop=True)

print "Done!"

print "------------------------\n\n"

if name == "Sapur":

print "\n\n------------------------"

print "Starting", name, "analysis."

datasapur=grouped.get_group("Sapur")

datasapur.columns = data.columns

#check if the file is in the provided folder

if salix == "" :

print "\nYou should add salix to the input databases files."

print "Check if the file exists"

sys.exit(1)

inputsapur = pd.read_csv(salix, sep='\t')

print "Database file is: ", salix

datasapur = merge(datasapur, inputsapur, left_on='subjectid', right_on='proteinID')

inputsapur=[]

datasapur = datasapur.drop(['proteinID', 'ProteinLength', 'description', 'DataBase', 'GeneID', 'transcriptID'],1)

datasapur.loc[:,'ID']="0"

datasapur.loc[:,'KEGG']="0"

datasapur.loc[:,'Taxonomy']="Eukaryota;Viridiplantae;Streptophyta;Streptophytina;Embryophyta;Tracheophyta;Euphyllophyta;Spermatophyta;Magnoliophyta;Mesangiospermae;eudicotyledons;Gunneridae;Pentapetalae;rosids;fabi ds;Malpighiales;Salicaceae;Saliceae;Salix;Salix purpurea"

datasapur=datasapur.rename(columns = {'KOG':'KOGAnnotation'})

datasapur=datasapur.rename(columns = {'KEGGOrthology':'KO'})

datasapur =datasapur[['queryid', 'geneid', 'identity', 'evalue', 'bitscore', 'bitscore2', 'querylength', 'Alignment_vs_Q_SeqLength', 'subjectid', 'ID', 'subjectlength', 'Alignment_vs_S_SeqLength', 'subjectdescription', 'GO', 'PFAM', 'Panther', 'KEGG', 'KEGGec', 'KO', 'KOGAnnotation', 'Taxonomy']]

sapur_size = len(datasapur)

data2= datasapur.append(data2).reset_index(drop=True)

print "Done!"

print "------------------------\n\n"

if name == "Potri":

print "\n\n------------------------"

print "Starting", name, "analysis."

dataPotri=grouped.get_group("Potri")

dataPotri.columns = data.columns

#check if the file is in the provided folder

if poplar == "" :

print "\nYou should add poplar to the input databases files."

print "Check if the file exists"

sys.exit(1)

inputPotri = pd.read_csv(poplar, sep='\t')

print "Database file is: ", poplar

dataPotri = merge(dataPotri, inputPotri, left_on='subjectid', right_on='proteinID')

inputPotri=[]

dataPotri = dataPotri.drop(['proteinID', 'ProteinLength', 'description', 'DataBase', 'GeneID', 'transcriptID'],1)

dataPotri.loc[:,'ID']="0"

dataPotri.loc[:,'KEGG']="0"

dataPotri.loc[:,'Taxonomy']="Eukaryota;Viridiplantae;Streptophyta;Streptophytina;Embryophyta;Tracheophyta;Euphyllophyta;Spermatophyta;Magnoliophyta;Mesangiospermae;Eudicotyledons;Gunneridae;Pentapetalae;Rosids;Fabi ds;Malpighiales;Salicaceae;Saliceae;Populus;Populus trichocarpa"

dataPotri=dataPotri.rename(columns = {'KOG':'KOGAnnotation'})

dataPotri=dataPotri.rename(columns = {'KEGGOrthology':'KO'})

dataPotri =dataPotri[['queryid', 'geneid', 'identity', 'evalue', 'bitscore', 'bitscore2', 'querylength', 'Alignment_vs_Q_SeqLength', 'subjectid', 'ID', 'subjectlength', 'Alignment_vs_S_SeqLength', 'subjectdescription', 'GO', 'PFAM', 'Panther', 'KEGG', 'KEGGec', 'KO', 'KOGAnnotation', 'Taxonomy']]

Potri_size = len(dataPotri)

data2= dataPotri.append(data2).reset_index(drop=True)

print "Done!"

print "------------------------\n\n"

taxa1 = data2['Taxonomy'].apply(lambda x: pd.Series(x.split(';',3)))

cols = taxa1.columns.tolist()

cols = cols[0:3]

taxa1 = taxa1[cols]

taxa1.columns = ['taxon1', 'taxon2', 'taxon3']

taxa2 = data2['Taxonomy'].apply(lambda x: pd.Series(x.rsplit(';',3)))

cols = taxa2.columns.tolist()

cols = cols[1:4]

taxa2 = taxa2[cols]

taxa2.columns = ['taxon4', 'taxon5', 'taxon6']

#removing unwanted spaces

taxa2['taxon4'] = taxa2.taxon4.str.replace('\n', '')

taxa2['taxon5'] = taxa2.taxon5.str.replace('\n', '')

taxa2['taxon6'] = taxa2.taxon6.str.replace('\n', '')

taxa=taxa1.join(taxa2, how='outer')

#Change 0 or no value with Unknown

taxa.taxon1.ix[taxa.taxon1 == '0'] = 'Unknown'

taxa.taxon2.ix[taxa.taxon2 == '0'] = 'Unknown'

taxa.taxon3.ix[taxa.taxon3 == '0'] = 'Unknown'

taxa.taxon4.ix[taxa.taxon4 == '0'] = 'Unknown'

taxa.taxon5.ix[taxa.taxon5 == '0'] = 'Unknown'

taxa.taxon6.ix[taxa.taxon6 == '0'] = 'Unknown'

taxa.taxon1.ix[taxa.taxon1 == ''] = 'Unknown'

taxa.taxon2.ix[taxa.taxon2 == ''] = 'Unknown'

taxa.taxon3.ix[taxa.taxon3 == ''] = 'Unknown'

taxa.taxon4.ix[taxa.taxon4 == ''] = 'Unknown'

taxa.taxon5.ix[taxa.taxon5 == ''] = 'Unknown'

taxa.taxon6.ix[taxa.taxon6 == ''] = 'Unknown'

taxa.fillna('Unknown', inplace=True)

data2=data2.join(taxa, how='outer')

firstax = taxa[['taxon1']].drop_duplicates()

sectax = taxa[['taxon2']].drop_duplicates()

thirdtax = taxa[['taxon3']].drop_duplicates()

lasttax = taxa[['taxon6']].drop_duplicates()

tax1= list(taxa.taxon1.values)

tax2= list(taxa.taxon2.values)

tax3= list(taxa.taxon3.values)

tax6= list(taxa.taxon6.values)

tax1_dict = counter(tax1)

tax2_dict = counter(tax2)

tax3_dict = counter(tax3)

tax6_dict = counter(tax6)

#Creating formating-correct tables to build an histogram or a pie chart

hist_data1 = DataFrame([tax1_dict[key] for key in tax1_dict],columns = ['Frequency'],index=tax1_dict.keys()).sort(columns='Frequency', ascending=False)

hist_data2 = DataFrame([tax2_dict[key] for key in tax2_dict],columns = ['Frequency'],index=tax2_dict.keys()).sort(columns='Frequency', ascending=False)

hist_data3 = DataFrame([tax3_dict[key] for key in tax3_dict],columns = ['Frequency'],index=tax3_dict.keys()).sort(columns='Frequency', ascending=False)

hist_data6 = DataFrame([tax6_dict[key] for key in tax6_dict],columns = ['Frequency'],index=tax6_dict.keys()).sort(columns='Frequency', ascending=False)

datatotal=len(data2)

temp1_1 = ((hist_data1.Frequency).astype(float) / datatotal* 100)

temp1_2 = temp1_1.ix[(temp1_1 >= 1.0 ) ]

temp1_3 = temp1_1.ix[(temp1_1 < 1.0 ) ]

others = Series([temp1_3.sum()], index=['Others'])

final1 = temp1_2.append(others)

final1 = final1.map(lambda x: '%2.1f' % x)

final1= final1[final1 != "0.0"]

final1 = final1.astype(float)

gbplot_pie(fractions = final1,

labels = final1.index,

title = 'Domain diversity (%i transcripts)'% datatotal,

savename = "%s_Domain.pdf" % outputfile)

temp2_1 = ((hist_data2.Frequency).astype(float) / datatotal* 100)

temp2_2 = temp2_1.ix[(temp2_1 >= 1.0 ) ]

temp2_3 = temp2_1.ix[(temp2_1 < 1.0 ) ]

others = Series([temp2_3.sum()], index=['Others'])

final2 = temp2_2.append(others)

final2 = final2.map(lambda x: '%2.1f' % x)

final2= final2[final2 != "0.0"]

gbplot_pie(fractions = final2,

labels = final2.index,

title = 'Kingdom diversity (%i transcripts)'% datatotal,

savename = "%s_Kingdom.pdf" % outputfile)

temp3_1 = ((hist_data3.Frequency).astype(float) / datatotal* 100)

temp3_2 = temp3_1.ix[(temp3_1 >= 1.0 ) ]

temp3_3 = temp3_1.ix[(temp3_1 < 1.0 ) ]

others = Series([temp3_3.sum()], index=['Others'])

final3 = temp3_2.append(others)

final3 = final3.map(lambda x: '%2.1f' % x)

final3= final3[final3 != "0.0"]

gbplot_pie(fractions = final3,

labels = final3.index,

title = 'Phylum diversity (%i transcripts)'% datatotal,

savename = "%s_Phylum.pdf" % outputfile)

temp6_1 = ((hist_data6.Frequency).astype(float) / datatotal* 100)

temp6_2 = temp6_1.ix[(temp6_1 >= 1.0 ) ]

temp6_3 = temp6_1.ix[(temp6_1 < 1.0 ) ]

others = Series([temp6_3.sum()], index=['Others'])

final6 = temp6_2.append(others)

final6 = final6.map(lambda x: '%2.1f' % x)

final6= final6[final6 != "0.0"]

gbplot_pie(fractions = final6,

labels = final6.index,

title = 'Species diversity (%i transcripts)'% datatotal,

savename = "%s_Species.pdf" % outputfile)

data2 = data2[['queryid', 'geneid', 'identity', 'evalue', 'bitscore', 'bitscore2', 'querylength', 'Alignment_vs_Q_SeqLength', 'subjectid', 'ID', 'subjectlength', 'Alignment_vs_S_SeqLength', 'subjectdescription', 'GO', 'PFAM', 'Panther', 'KEGG', 'KEGGec', 'KO', 'KOGAnnotation', 'taxon1', 'taxon2', 'taxon3', 'taxon4', 'taxon5', 'taxon6', 'Taxonomy']]

data=data2

print "\n Annotation SUMMARY: "

print "-------------------------------"

print "Number of nr hits : ", nr_size

print "Number of trembl hits : ", tr_size

print "Number of swissprot hits : ", sp_size

print "Number of uniparc hits : ", uniparc_size

print "Number of salix purpurea hits : ", sapur_size

print "Number of populus trichocarpa hits : ", Potri_size

if evalue == 10 and identity == 101 and qcutoff == 0 and scutoff == 0 and qwords == "" and swords == "" and annotation == "":

pass

else:

data= data.sort(columns=['geneid', 'queryid', 'bitscore2'], ascending=[True, True, False]).reset_index(drop=True)

data['evalue']= data['evalue'].astype(str)

print "Writing output file to disk."

print "Outputfile has : ", "{:,}".format(data.shape[0]), " records"

df_test=data.drop_duplicates('queryid')

print "Outputfile has : ", "{:,}".format(df_test.shape[0]), " unique records"

print "Output file name : ", outputfile

data.to_csv(outputfile, sep='\t', index=False)

print "done."

# Bitscore2 parser suite – 4

Uses as inpufile a file that has already been processed by bitscoreparser_part3.py. This script will choose the best annotation per contig good annotation hits and will keep the rejected hits as a secondary annotation information. The output of this script is the final output in our article.

#!/usr/bin/python

import numpy as np

from scipy import randn

from datetime import datetime

import argparse

import pandas as pd

from pandas import *

import matplotlib

matplotlib.use('Agg')

import matplotlib.pyplot as plt

import locale

import re

import sys

from scipy import stats

from scipy.stats import gaussian_kde

from pandas.tools.plotting import table

import os

import matplotlib.gridspec as gridspec

import matplotlib.cm as cm, matplotlib.font_manager as fm

import operator

print "Pandas version : " + pandas.__version__

print "Matplotlib version : " + matplotlib.__version__

RowCollapse = lambda x:";".join(x.astype(str))

def myround(x, base):

return int(base * round(float(x)/base))

def mostcommonwordsinstring(query):

word_counter = {}

for word in query.split(" "): # split in every space.

if len(word) > 2 and word != '\r\n': #we don't want the noise words like letters, of, at, etc...

if word not in word_counter: # if 'word' not in word_counter, add it, and set value to 1

word_counter[word] = 1

else:

word_counter[word] += 1 # if 'word' already in word_counter, increment it by 1

wordranking = sorted(word_counter.items(), key=operator.itemgetter(1),reverse=True)

wordranking = list(wordranking[:5])

return wordranking

startTime = datetime.now()

parser = argparse.ArgumentParser(description="Uses as inpufile (inputfile_b2parsed3.txt) a file that has already been processed by bitscoreparser_part3.py. ", epilog = "The output file will look like : inputfile_prefix_isoforms_annotated.txt")

parser.add_argument('-i', '--blastoutfile', help='[REQUIRED] File from bitscoreparser_part3.py. This script expects 27 columns : queryid|geneid|identity|evalue|bitscore|bitscore2|querylength|Alignment_vs_Q_SeqLength|subjectid|ID|subjectlength|Alignment_vs_S_SeqLength|subjectdescription|GO|PFAM|Panther|KEGG|KEGGec|KO|KOGAnnotation|taxon1|taxon2|taxon3|taxon4|taxon5|taxon6|Taxonomy. Headers are assumed, first row will be skipped. Input file example : -b filename_isoforms_alignpercent.txt', dest='inputfile1', action='store', required=True)

parser.add_argument('-t', '--trinity', help='[OPTIONAL] Subject sequences are Trinity subject sequences (e.g comp1_c0_seq1). If -t is applied, two files will be generated: one for isoforms and one for genes.', dest='trinity', default=False, action='store_true')

args = parser.parse_args()

inputfile1 = args.inputfile1

Trinity = args.trinity

data = pd.read_csv(inputfile1, sep='\t')

#rounding bitscore2

data['bitscore2'] = data['bitscore2'].map(lambda x: myround(x,1))

data['bitscore'] = data['bitscore'].map(lambda x: myround(x,1))

print '\n'

print " INPUT SUMMARY "

print "-------------------------------"

print ('Inputfile : ' + str(inputfile1))

print "Inputfile has : ", "{:,}".format(data.shape[0]), " records"

print "Highest evalue : ", data.ix[data['evalue'].argmax(),'evalue']

print "Mean evalue value : ", "{:.0e}".format(data['evalue'].mean())

print "Mean bitscore2 :", data['bitscore2'].mean().astype(int), "%"

print "Lowest bitscore2 :", data.ix[data['bitscore2'].argmin(),'bitscore2'].astype(int), "%"

print "Mean blast alignement identity :", data['identity'].mean().astype(int), "%"

print "Lowest blast alignement identity :", data.ix[data['identity'].argmin(),'identity'].astype(int), "%"

print "Lowest subject sequence vs alignment percentage:", data.ix[data['Alignment_vs_S_SeqLength'].argmin(),'Alignment_vs_S_SeqLength'].astype(int), "%"

print "Maximum subject sequence vs alignment percentage:", data.ix[data['Alignment_vs_S_SeqLength'].argmax(),'Alignment_vs_S_SeqLength'].astype(int), "%"

print "Mean subject sequence vs alignment percentage :", data['Alignment_vs_S_SeqLength'].mean().astype(int), "%"

print "-------------------------------"

print "\n-----------------------------------------"

print "STEP 1: Order blast hits depending on 3 factors: bitscore2, species priority, and annotation quality."

print "Right now, data have", "{:,}".format(data.shape[0]), "records."

print "\n First factor: bitscore2."

#No need to do anything here, bitscore2 has been recalculated in bitscoreparser_part2.py

print "\n Second factor: species priority."

data['subjectid'].replace(to_replace="Potri", value="Potri|", inplace=True,regex=True)

data['subjectid'].replace(to_replace="SapurV", value="Sapur|V", inplace=True,regex=True)

data['subjectid'].replace(to_replace="tetur", value="tetur|", inplace=True,regex=True)

#Now that all highest evalues were selected, we will prioritize salix, poplar, swissprot, and then trembl. nr will thus be our lowest priority.

data['DataBase']=[x.split("|")[0] for x in data['subjectid'].astype(str)]

data['subjectid'].replace(to_replace="Potri\|", value="Potri", inplace=True, regex=True)

data['subjectid'].replace(to_replace="Sapur\|", value="Sapur", inplace=True, regex=True)

data['subjectid'].replace(to_replace="tetur\|", value="tetur", inplace=True, regex=True)

#Assigning rank value for swissrot and trembl output.

data['rank']=0

data.ix[data.DataBase == 'Sapur', ['rank']] =100000

data.ix[data.DataBase == 'Potri', ['rank']] =100

data.ix[data.DataBase == 'tetur', ['rank']] =100

data.ix[data.DataBase == 'laccaria', ['rank']] =100

data.ix[data.DataBase == 'pyronema', ['rank']] =100

data.ix[data.DataBase == 'ecoli', ['rank']] =100

data.ix[data.DataBase == 'tuber', ['rank']] =100

data.ix[data.DataBase == 'sp', ['rank']] =100

data.ix[data.DataBase == 'tr', ['rank']] =100

data.ix[data.DataBase == 'uniparc', ['rank']] =100

data.ix[data.DataBase == 'gi', ['rank']] =100

data = data.drop('DataBase',1)

print( 'So far : ' + str(datetime.now()-startTime) + ' h:min:ss')

print "\n Third factor: Annotation quality."

pattern = 'NoDescription|Uncharacterized|uncharacterized|Unknown|unknown|hypothetical|Hypothetical'

data['20points_1'] = data.subjectdescription.str.contains(pattern)

data['20points']=0

data.loc[data['20points_1'] == True, '20points'] = -20

data = data.drop('20points_1',1)

#pattern = "putative|Putative"

#data['5points_1'] = data.subjectdescription.str.contains(pattern)

#data['5points']=0

#data.loc[data['5points_1'] == True, '5points'] = -5

#data = data.drop('5points_1',1)

data['GOpoints']=5

data.loc[:,'GO']=data.loc[:,'GO'].astype(str)

data.loc[data['GO'] == "0", 'GOpoints'] = 0

data['PFAMpoints']=5

data.loc[:,'PFAM']=data.loc[:,'PFAM'].astype(str)

data.loc[data['PFAM'] == "0", 'PFAMpoints'] = 0

data['Pantherpoints']=5

data.loc[:,'Panther']=data.loc[:,'Panther'].astype(str)

data.loc[data['Panther'] == "0", 'Pantherpoints'] = 0

data['KEGGpoints']=5

data.loc[:,'KEGG']=data.loc[:,'KEGG'].astype(str)

data.loc[data['KEGG'] == "0", 'KEGGpoints'] = 0

data['KEGGecpoints']=5

data.loc[:,'KEGGec']=data.loc[:,'KEGGec'].astype(str)

data.loc[data['KEGGec'] == "0", 'KEGGecpoints'] = 0

data['KOpoints']=5

data.loc[:,'KO']=data.loc[:,'KO'].astype(str)

data.loc[data['KO'] == "0", 'KOpoints'] = 0

data['KOGAnnotationpoints']=5

data.loc[:,'KOGAnnotation']=data.loc[:,'KOGAnnotation'].astype(str)

data.loc[data['KOGAnnotation'] == "0", 'KOGAnnotationpoints'] = 0

data["totalpoints"]=0

data["totalpoints"]=20+data['20points']+data['GOpoints']+data['PFAMpoints']+\

data['Pantherpoints']+data['KEGGpoints']+data['KEGGecpoints']+data['KOpoints']+data['KOGAnnotationpoints']

data = data.drop(['20points','GOpoints','PFAMpoints','Pantherpoints','KEGGpoints','KEGGecpoints','KOpoints','KOGAnnotationpoints'], 1)

print( 'So far : ' + str(datetime.now()-startTime) + ' h:min:ss')

print "\n Sorting data by the 3 factors"

mysort = lambda x: x.sort_values(by=['bitscore2', 'rank', 'totalpoints'], ascending=[False, False, False])

data = data.groupby('queryid').apply(mysort)

print( 'So far : ' + str(datetime.now()-startTime) + ' h:min:ss')

print "\n Keeping max 10 records per hit"

def f(df):

return df.iloc[:10]

data = data.groupby('queryid', group_keys=False).apply(f)

print( 'So far : ' + str(datetime.now()-startTime) + ' h:min:ss')

print "Done! data have", "{:,}".format(data.shape[0]), "records left"

print "-----------------------------------------\n"

print "\n-----------------------------------------"

print "STEP 2: Secondary annotation. Extract all remaining blast hits information i.e.", "{:,}".format(data.shape[0]), "records"

data['subjectdescription'] = data['subjectdescription'].map(lambda x: x.split(' OS=')[0])

print( '\n Remove OS in definition. So far : ' + str(datetime.now()-startTime) + ' h:min:ss')

data2=data.groupby("queryid").agg({"subjectid":RowCollapse,"taxon6":RowCollapse,"bitscore":RowCollapse,"bitscore2":RowCollapse,"subjectdescription":RowCollapse,

"GO":RowCollapse,"PFAM":RowCollapse,"Panther":RowCollapse,

"KEGG":RowCollapse,"KEGGec":RowCollapse,"KO":RowCollapse,

"KOGAnnotation":RowCollapse})

print( '\n Create secondary annotation. So far : ' + str(datetime.now()-startTime) + ' h:min:ss')

data2['keywords']="None" #prepare the keywords column

data2=data2[["subjectid", "taxon6", "bitscore", "bitscore2", "keywords", "subjectdescription","GO","PFAM","Panther",

"KEGG","KEGGec","KO","KOGAnnotation"]]

data2 = data2.replace({'\n': ''}, regex=True)

data2 = data2.replace({'; ': ';'}, regex=True)

data2 = data2.replace({';': ' ; '}, regex=True)

data2.columns=data2.columns+"_2"

data2=data2.reset_index(drop=False)

print( '\n Rearranging annotation. So far : ' + str(datetime.now()-startTime) + ' h:min:ss')

i=0

for line in data2.subjectdescription_2:

query = data2.ix[i,'subjectdescription_2'].replace('protein', '')

word_counter = {}

for word in query.split(" "): # split in every space.

if len(word) > 2 and word != '\r\n': #we don't want the noise words like letters, of, at, etc...

if word not in word_counter: # if 'word' not in word_counter, add it, and set value to 1

word_counter[word] = 1

else:

word_counter[word] += 1 # if 'word' already in word_counter, increment it by 1

wordranking = sorted(word_counter.items(), key=operator.itemgetter(1),reverse=True) #sort by highest value of tuple

wordranking = list(wordranking[:5])

data2.loc[i,'keywords_2'] = wordranking

i=i+1

print( '\n keywords_2. So far : ' + str(datetime.now()-startTime) + ' h:min:ss')

i=0

for line in data2.subjectdescription_2:

seen = []

words = line.strip().split(';')

for word in words:

if not word in seen:

seen.append(word)

seen = ';'.join(seen)

data2.loc[i,'subjectdescription_2'] = seen

i=i+1

print( '\n subjectdescription_2. So far : ' + str(datetime.now()-startTime) + ' h:min:ss')

i=0

for line in data2.GO_2:

seen = []

words = line.strip().split(';')

for word in words:

if not word in seen:

seen.append(word)

seen = ';'.join(seen)

data2.loc[i,'GO_2'] = seen

i=i+1

print( '\n GO_2. So far : ' + str(datetime.now()-startTime) + ' h:min:ss')

i=0

for line in data2.PFAM_2:

seen = []

words = line.strip().split(';')

for word in words:

if not word in seen:

seen.append(word)

seen = ';'.join(seen)

data2.loc[i,'PFAM_2'] = seen

i=i+1

print( '\n PFAM_2. So far : ' + str(datetime.now()-startTime) + ' h:min:ss')

i=0

for line in data2.Panther_2:

seen = []

words = line.strip().split(';')

for word in words:

if not word in seen:

seen.append(word)

seen = ';'.join(seen)

data2.loc[i,'Panther_2'] = seen

i=i+1

print( '\n Panther_2. So far : ' + str(datetime.now()-startTime) + ' h:min:ss')

i=0

for line in data2.KEGG_2:

seen = []

words = line.strip().split(';')

for word in words:

if not word in seen:

seen.append(word)

seen = ';'.join(seen)

data2.loc[i,'KEGG_2'] = seen

i=i+1

print( '\n KEGG_2. So far : ' + str(datetime.now()-startTime) + ' h:min:ss')

i=0

for line in data2.KEGGec_2:

seen = []

words = line.strip().split(';')

for word in words:

if not word in seen:

seen.append(word)

seen = ';'.join(seen)

data2.loc[i,'KEGGec_2'] = seen

i=i+1

print( '\n KEGGec_2. So far : ' + str(datetime.now()-startTime) + ' h:min:ss')

i=0

for line in data2.KO_2:

seen = []

words = line.strip().split(';')

for word in words:

if not word in seen:

seen.append(word)

seen = ';'.join(seen)

data2.loc[i,'KO_2'] = seen

i=i+1

print( '\n KO_2. So far : ' + str(datetime.now()-startTime) + ' h:min:ss')

i=0

for line in data2.KOGAnnotation_2:

seen = []

words = line.strip().split(';')

for word in words:

if not word in seen:

seen.append(word)

seen = ';'.join(seen)

data2.loc[i,'KOGAnnotation_2'] = seen

i=i+1

print( '\n KOGAnnotation_2. So far : ' + str(datetime.now()-startTime) + ' h:min:ss')

data2 = data2.replace({' ; ': ';'}, regex=True)

data2 = data2.replace({';;': ';'}, regex=True)

data2 = data2.replace({'^0;': ''}, regex=True)

data2 = data2.replace({';0$': ''}, regex=True)

print "Done! Data have", "{:,}".format(data2.shape[0]), "distinct records"

print "-----------------------------------------\n"

print "\n-----------------------------------------"

print "STEP 3: Best hit per contig"

print "\n Sorting data by the 3 factors define in step 1"

mysort = lambda x: x.sort_values(by=['bitscore2', 'rank', 'totalpoints'], ascending=[False, False, False])

data = data.groupby('queryid').apply(mysort)

print "\n Keeping only the best record per contig."

def f(df):

return df.iloc[:1]

data = data.groupby('queryid', group_keys=False).apply(f)

data = data.drop(['rank','totalpoints'], 1)

print( 'So far : ' + str(datetime.now()-startTime) + ' h:min:ss')

print "Done! data have", "{:,}".format(data.shape[0]), "records left"

print "-----------------------------------------\n"

print "\n-----------------------------------------"

print "Append all information previously gathered to this data"

data=data.merge(data2, on='queryid', how='left')

data2 = data[["geneid","subjectid","taxon6_2" , "bitscore_2", "bitscore2_2","keywords_2", "subjectdescription_2","GO_2","PFAM_2","Panther_2", "KEGG_2","KEGGec_2","KO_2","KOGAnnotation_2"]]

print( 'So far : ' + str(datetime.now()-startTime) + ' h:min:ss')

print "Done! data have", "{:,}".format(data.shape[0]), "records left"

print "-----------------------------------------\n"

if Trinity == True:

outputfileiso = inputfile1.split('_b2parsed3.txt')[0] + "_b2parsed4_isoforms.txt"

shapeiso=data.shape[0]

data.to_csv(outputfileiso, sep='\t', index=False)

print "Now working for genes."

print "\n-----------------------------------------"

print "Extracting all information from blast hits that passed quality filters i.e.", "{:,}".format(data.shape[0]), "records"

#remove contigs

data = data.drop('queryid',1)

#rename geneid as queryid. This is just to reuse the code above. I'll rename that column geneid at the end of the code.

data=data.rename(columns = {'geneid':'queryid'})

data['queryid'] = [x.split("_i")[0] for x in data['queryid']]

print "\n-----------------------------------------"

print "STEP 1: Order blast hits depending on 3 factors: bitscore2, species priority, and annotation quality."

print "\n Right now, data have", "{:,}".format(data.shape[0]), "records."

print "\n First factor: bitscore2."

#No need to do anything here, bitscore2 has been recalculated in bitscoreparser_part2.py

print "\n Second factor: species priority."

data['subjectid'].replace(to_replace="Potri", value="Potri|", inplace=True,regex=True)

data['subjectid'].replace(to_replace="SapurV", value="Sapur|V", inplace=True,regex=True)

data['subjectid'].replace(to_replace="tetur", value="tetur|", inplace=True,regex=True)

#Now that all highest evalues were selected, we will prioritize salix, poplar, swissprot, and then trembl. nr will thus be our lowest priority.

data['DataBase']=[x.split("|")[0] for x in data['subjectid'].astype(str)]

data['subjectid'].replace(to_replace="Potri\|", value="Potri", inplace=True, regex=True)

data['subjectid'].replace(to_replace="Sapur\|", value="Sapur", inplace=True, regex=True)

data['subjectid'].replace(to_replace="tetur\|", value="tetur", inplace=True, regex=True)

#Assigning rank value for swissrot and trembl output.

data['rank']=0

data.ix[data.DataBase == 'Sapur', ['rank']] =100000

data.ix[data.DataBase == 'Potri', ['rank']] =100

data.ix[data.DataBase == 'tetur', ['rank']] =100

data.ix[data.DataBase == 'laccaria', ['rank']] =100

data.ix[data.DataBase == 'pyronema', ['rank']] =100

data.ix[data.DataBase == 'ecoli', ['rank']] =100

data.ix[data.DataBase == 'tuber', ['rank']] =100

data.ix[data.DataBase == 'sp', ['rank']] =100

data.ix[data.DataBase == 'tr', ['rank']] =100

data.ix[data.DataBase == 'uniparc', ['rank']] =100

data.ix[data.DataBase == 'gi', ['rank']] =100

data = data.drop('DataBase',1)

print( '\n So far : ' + str(datetime.now()-startTime) + ' h:min:ss')

print "\n Third factor: Annotation quality."

pattern = 'NoDescription|Uncharacterized|uncharacterized|Unknown|unknown|hypothetical|Hypothetical'

data['20points_1'] = data.subjectdescription.str.contains(pattern)

data['20points']=0

data.loc[data['20points_1'] == True, '20points'] = -20

data = data.drop('20points_1',1)

#pattern = "putative|Putative"

#data['5points_1'] = data.subjectdescription.str.contains(pattern)

#data['5points']=0

#data.loc[data['5points_1'] == True, '5points'] = -5

#data = data.drop('5points_1',1)

data['GOpoints']=5

data.loc[data['GO'] == "0", 'GOpoints'] = 0

data['PFAMpoints']=5

data.loc[data['PFAM'] == "0", 'PFAMpoints'] = 0

data['Pantherpoints']=5

data.loc[data['Panther'] == "0", 'Pantherpoints'] = 0

data['KEGGpoints']=5

data.loc[data['KEGG'] == "0", 'KEGGpoints'] = 0

data['KEGGecpoints']=5

data.loc[data['KEGGec'] == "0", 'KEGGecpoints'] = 0

data['KOpoints']=5

data.loc[data['KO'] == "0", 'KOpoints'] = 0

data['KOGAnnotationpoints']=5

data.loc[data['KOGAnnotation'] == "0", 'KOGAnnotationpoints'] = 0

data["totalpoints"]=0

data["totalpoints"]=20+data['20points']+data['GOpoints']+data['PFAMpoints']+\

data['Pantherpoints']+data['KEGGpoints']+data['KEGGecpoints']+data['KOpoints']+data['KOGAnnotationpoints']

data = data.drop(['20points','GOpoints','PFAMpoints','Pantherpoints','KEGGpoints','KEGGecpoints','KOpoints','KOGAnnotationpoints'], 1)

print( '\n So far : ' + str(datetime.now()-startTime) + ' h:min:ss')

print "\n Sorting data by the 3 factors"

mysort = lambda x: x.sort_values(by=['bitscore2', 'rank', 'totalpoints'], ascending=[False, False, False])

data = data.groupby('queryid').apply(mysort)

print( '\n So far : ' + str(datetime.now()-startTime) + ' h:min:ss')

print "\n Done! data have", "{:,}".format(data.shape[0]), "records left"

print "-----------------------------------------\n"

print "\n-----------------------------------------"

print "STEP 2: Secondary annotation. Extract all remaining blast hits information i.e.", "{:,}".format(data.shape[0]), "records"

data2=data.groupby("queryid").agg({"subjectid":RowCollapse,"taxon6":RowCollapse,"bitscore":RowCollapse,

"bitscore2":RowCollapse,"subjectdescription":RowCollapse,

"GO":RowCollapse,"PFAM":RowCollapse,"Panther":RowCollapse,

"KEGG":RowCollapse,"KEGGec":RowCollapse,"KO":RowCollapse,

"KOGAnnotation":RowCollapse})

print( '\n Create secondary annotation. So far : ' + str(datetime.now()-startTime) + ' h:min:ss')

data2['keywords']="None" #prepare the keywords column

data2=data2[["subjectid", "taxon6", "bitscore", "bitscore2", "keywords", "subjectdescription","GO","PFAM","Panther",

"KEGG","KEGGec","KO","KOGAnnotation"]]

data2 = data2.replace({'\n': ''}, regex=True)

data2 = data2.replace({'; ': ';'}, regex=True)

data2 = data2.replace({';': ' ; '}, regex=True)

data2.columns=data2.columns+"_2"

data2=data2.reset_index(drop=False)

print( '\n Rearranging annotation. So far : ' + str(datetime.now()-startTime) + ' h:min:ss')

#keywords: we will rank the number of occurence of a word and extract the five most occurent.

i=0

for line in data2.subjectdescription_2:

query = data2.ix[i,'subjectdescription_2'].replace('protein', '')

word_counter = {}

for word in query.split(" "): # split in every space.

if len(word) > 2 and word != '\r\n': #we don't want the noise words like letters, of, at, etc...

if word not in word_counter: # if 'word' not in word_counter, add it, and set value to 1

word_counter[word] = 1

else:

word_counter[word] += 1 # if 'word' already in word_counter, increment it by 1

wordranking = sorted(word_counter.items(), key=operator.itemgetter(1),reverse=True) #sort by highest value of tuple

wordranking = list(wordranking[:5])

data2.loc[i,'keywords_2'] = wordranking

i=i+1

print( '\n keywords_2. So far : ' + str(datetime.now()-startTime) + ' h:min:ss')

i=0

for line in data2.subjectdescription_2:

seen = []

words = line.strip().split(';')

for word in words:

if not word in seen:

seen.append(word)

seen = ';'.join(seen)

data2.loc[i,'subjectdescription_2'] = seen

i=i+1

print( '\n subjectdescription_2. So far : ' + str(datetime.now()-startTime) + ' h:min:ss')

i=0

for line in data2.GO_2:

seen = []

words = line.strip().split(';')

for word in words:

if not word in seen:

seen.append(word)

seen = ';'.join(seen)

data2.loc[i,'GO_2'] = seen

i=i+1

print( '\n GO_2. So far : ' + str(datetime.now()-startTime) + ' h:min:ss')

i=0

for line in data2.PFAM_2:

seen = []

words = line.strip().split(';')

for word in words:

if not word in seen:

seen.append(word)

seen = ';'.join(seen)

data2.loc[i,'PFAM_2'] = seen

i=i+1

print( '\n PFAM_2. So far : ' + str(datetime.now()-startTime) + ' h:min:ss')

i=0

for line in data2.Panther_2:

seen = []

words = line.strip().split(';')

for word in words:

if not word in seen:

seen.append(word)

seen = ';'.join(seen)

data2.loc[i,'Panther_2'] = seen

i=i+1

print( '\n Panther_2. So far : ' + str(datetime.now()-startTime) + ' h:min:ss')

i=0

for line in data2.KEGG_2:

seen = []

words = line.strip().split(';')

for word in words:

if not word in seen:

seen.append(word)

seen = ';'.join(seen)

data2.loc[i,'KEGG_2'] = seen

i=i+1

print( '\n KEGG_2. So far : ' + str(datetime.now()-startTime) + ' h:min:ss')

i=0

for line in data2.KEGGec_2:

seen = []

words = line.strip().split(';')

for word in words:

if not word in seen:

seen.append(word)

seen = ';'.join(seen)

data2.loc[i,'KEGGec_2'] = seen

i=i+1

print( '\n KEGGec_2. So far : ' + str(datetime.now()-startTime) + ' h:min:ss')

i=0

for line in data2.KO_2:

seen = []

words = line.strip().split(';')

for word in words:

if not word in seen:

seen.append(word)

seen = ';'.join(seen)

data2.loc[i,'KO_2'] = seen

i=i+1

print( '\n KO_2. So far : ' + str(datetime.now()-startTime) + ' h:min:ss')

i=0

for line in data2.KOGAnnotation_2:

seen = []

words = line.strip().split(';')

for word in words:

if not word in seen:

seen.append(word)

seen = ';'.join(seen)

data2.loc[i,'KOGAnnotation_2'] = seen

i=i+1

print( '\n KOGAnnotation_2. So far : ' + str(datetime.now()-startTime) + ' h:min:ss')

data2 = data2.replace({' ; ': ';'}, regex=True)

data2 = data2.replace({';;': ';'}, regex=True)

data2 = data2.replace({'^0;': ''}, regex=True)

data2 = data2.replace({';0$': ''}, regex=True)

print "\n Done! Data have", "{:,}".format(data2.shape[0]), "distinct records"

print "-----------------------------------------\n"

print "\n-----------------------------------------"

print "STEP 3: Best hit per contig"

print "\n Sorting data by the 3 factors define in step 1"

mysort = lambda x: x.sort_values(by=['bitscore2', 'rank', 'totalpoints'], ascending=[False, False, False])

data = data.groupby('queryid').apply(mysort)

print "\n Keeping only the best record per contig."

def f(df):

return df.iloc[:1]

data = data.groupby('queryid', group_keys=False).apply(f)

data = data.drop(['rank','totalpoints'], 1)

print( '\n So far : ' + str(datetime.now()-startTime) + ' h:min:ss')

print "\n Done! data have", "{:,}".format(data.shape[0]), "records left"

print "-----------------------------------------\n"

print "\n-----------------------------------------"

print "Append all information previously gathered to this data"

data=data.merge(data2, on='queryid', how='left')

print( 'So far : ' + str(datetime.now()-startTime) + ' h:min:ss')

print "Done! data have", "{:,}".format(data.shape[0]), "records left"

print "-----------------------------------------\n"

data=data.rename(columns = {'queryid':'geneid'})

outputfile = inputfile1.split('_b2parsed3.txt')[0] + '_b2parsed4_genes.txt'

data.to_csv(outputfile, sep='\t', index=False)

print "\n OUTPUT INFORMATION: "

print "-------------------------------"

print ('Isoform output file name : ' + str(outputfileiso))

print "Isoform output file has : ", "{:,}".format(shapeiso), " records"

print ('Gene output file name : ' + str(outputfile))

print "Gene output file has : ", "{:,}".format(data.shape[0]), " records"

print( '\nWhole process took : ' + str(datetime.now()-startTime) + ' h:min:ss')

else:

outputfile = inputfile1.split('_b2parsed3.txt')[0] +"_b2parsed4.txt"

data.to_csv(outputfile, sep='\t', index=False)

print "\n OUTPUT INFORMATION: "

print "-------------------------------"

print ('Output file name : ' + str(outputfile))

print "Output file has : ", "{:,}".format(data.shape[0]), " records"

print( '\nWhole process took : ' + str(datetime.now()-startTime) + ' h:min:ss')

print "-------------------------------"

print "Nicely done!"

print( '\nWhole process took : ' + str(datetime.now()-startTime) + ' h:min:ss')
